# Prehospital cardiac arrest resuscitation practices differ around the globe

**DOI:** 10.1016/j.resplu.2025.101017

**Published:** 2025-06-24

**Authors:** Jeannett Kjær, Louise Milling, Anne Craveiro Brøchner, Freddy Lippert, Stig Nikolaj Blomberg, Helle Collatz Christensen, Robyn Holgate, Laurie J. Morrison, Abdullah Bakhsh, Søren Mikkelsen, The Global Prehospital Resuscitation Network, Loui K. Alsulimani, Stanislav Popela, Jana Kruba Vidunová, David Peran, Roman Gregor, René Papousek, Anđela Simić, Andrés Cairol, Vicente Sánchez-Brunete Ingelmo, Hjalti Már Björnsson, Pascal Stammet, Raffo Escalante-Kanashiro, Olympia Nikolaidou, Vlasios Karageorgos, Theodoros Aslanidis, Bernd Wallner, Martin Rief, Michael Eichinger, Siddha S.C. Chakra Rao, Baljit SinghMD, Alexei A. Birkun, Pablo Aguilera, Jan Bakker, Muhammad Sultan Zaher, Sultan Ali Alwajeeh, Seizan Tanabe, Taku Iwami, Shunsuke Saito, Juan-Manuel Fraga-Sastrias, June Eva Kittivo, Kephas Ochieng Achiro, Caroline Ndinda, Koen Monsieurs, Erwin Snijders, Naseef Abdullah, David Stanton, John Thomas Meyer, Steven John Crawford, Dorra Loghmari, Hela ben Turkia, Wiem Barbaria, Nilmini Wijesuriya, Rathnayake Mudiyanselage Dilruk Indika Rathnayake, Nathan Woltman, Faith Joan Mesa-Gaerlan, Pauline Convocar, Bernadett Pua Velasco, Hajriz Alihodžić, Ahmad Alrawashdeh, Mahmoud T. Alwidyan, Miguel Soares-Oliveira, Mauro Mota, Yu Cao, Peng Yao, Rex Pui Kin Lam, Arthur Chi-Kin Cheung, Bence Bogár, Peter Temesvari, Róbert Gebei, Pelin Karaaslan, Turhan Sofuoglu, Liviu Ciocan, Déborah Jaeger, Chih-Wei Sung, Chi-Hsin Chen, Mikael Gellerfors, Rebecka Rubenson Wahlin, Carl Otto Schell, Fergus Gardiner, Martin Nichols, Sam Perillo, David Reid, Stian Mohrsen, Alasdair R. Corfield, Marc Allen, Stefano Falcetta, Maurizio Menarini, Marius Rehn, Jo Kramer-Johansen, Per P. Bredmose, Theresa Mariero Olasveengen, Oddvar Uleberg, Thomas Wilson, Thomas W. Lindner, Lars Jacobsen, Amund Formo, Trond Elden, Mari Stokstad Olsen, Marcin Kowalski, Tomasz Derkowski, Peter Martin Hansen, Troels Martin Hansen, Leif Rognås, Jacob Steinmetz, Morten Thingemann Bøtker, Thomas Bech Lunen, Holger Wemmelund, Pierre-Nicolas Carron, Laurent Suppan, Robb Anthony DeVries, Elizabeth Froelich, Timothy Thornton, Christine Brent, Marvin Wayne, Michael R. Sayre, Gerard Job, Jeffrey D. Ferguson, Debra Perina, Charles J. Lane, Ken Miller, Christie Fritz, Jeremy DeWall, Joshua Stilley, Angela P. Cornelius, Jeffrey L. Jarvis, Eric Wu, Noah Bernhardson, Randy Katz, Joshua G. Corsa, Douglas C. Gruzd, Jessica Wentling, Jeffrey M. Goodloe, Alexander Zozula, Joseph E. Holley, Stephen J. Vetrano, Matthew LoConte, Damon Darsey, Aurora Lybeck, Todd Chassee, Johannes Björkman, Jouni Nurmi, Timo Iirola, Helena Jäntti, Jouni Kurola, Florian Reifferscheid, Marcus Rudolph, Peter Hilbert-Carius, Stephan Katzenschlager, Dennis G. Barten, Edward C.T.H. Tan, Ingvar Thore Benno Berg, Geert-Jan van Geffen, Jeroen Seesink, Gert-Jan van der Ploeg, Natalie Anderson, Andrew H. Swain, Andrew Fu Wah Ho, Chua Si Yong Ivan, Johannes von Vopelius-Feldt, Russell D. MacDonald, Josephine Ssewanyana, Agim Krasniqi, Dusabimana Simon, Unisa Kanu, Russell Bonnici Farrugia, Jacqueline Eleonora Ek, Mihaela Budimski Soldat, George Michagin, Vagn Bach, Nicolai Lohse, Daniela Aparecida Morais, Guillaume Alinier, Ricardo M. Romero, Ki-Ok Ahn, Matej Strnad, Marko Noc, Vesna Borovnik Lesjak, Andrej Markota, Pariwat Phungoen, Korakot Apiratwarakul, Siobhan Masterson, Cathal O’Donnell, Jahlelawati Zul, Jose Maria Naval Potro Pascual, Clint Jean Louis, Glenn Burket

**Affiliations:** fn Emergency Medicine, King Abdulaziz University, Jeddah, Saudi Arabia; fp Al-Malae’b St, Jeddah 22254, Saudi Arabia; fl EMS South Moravian region, Czech Republic; l EMS of Pilsen Region, Czech Republic; m Emergency Medical Services of Karlovy Vary Region, Czech Republic Department of Anaesthesia and Intensive, Care Medicine, Charles University, Third Faculty of Medicine and FNKV University Hospital in Prague, Czech Republic; n EMS of Moravian-Silesian Region, Czech Republic; o Medical Rescue Service of the South Bohemian Region p.o., Czech Republic; p Educational Institute of Emergency Medicine of Varazdin County, Croatia; q UNIBE COSTA RICA - Universidad de Iberoamerica, Costa Rica; r Yfirlæknir bráðaþjónustu utan sjúkrahúsa, Department of Emergency Medicine Landspitali University Hospital, Reykjavík, Iceland; s Department of Emergency Medicine Landspitali - The National University Hospital of Iceland, University of Iceland, Iceland; t Centre Hospitalier de Luxembourg, University of Luxembourg, Esch-sur-Alzette, Luxembourg; u Instituto Nacional de Salud del Niño Universidad Peruana de Ciencias Aplicadas, Peru; v National Center for Emergency Care (EMS Thessaloniki), Greece; w Cardiopulmonary Resuscitation Laboratory, School of Medicine, University of Crete, Heraklion, Crete, Greece; fm Department of Anesthesiology, Onassis Cardiac Surgery Center, Athens, Greece; x Intensive Care Unit & Anesthesiology Department Agios Pavlos General Hospital Thessaloniki, Greece; y Department of Anaesthesiology and General Intensive Care Medicine, Medizinische Universität Innsbruck, Austria; z Department of Anaesthesiology and Intensive Care Medicine, Medical University of Graz, Austria; aa Chairman Indian Resuscitation Council Federation, India; ab Tantia University, Sri Ganganagar (Rajasthan), India; ac Department of General Surgery, Anaesthesiology, Resuscitation and Emergency Medicine, Medical Institute named after S.I. Georgievsky of V.I. Vernadsky Crimean Federal University, Simferopol, Russian Federation, Russia; ad Escuela de Medicina Pontificia, Universidad Catolica de Chile, Chile; ae Pontificia Universidad Católica de Chile, Department of Intensive, Care Diagonal Paraguay Santiago, Chile; af Kau University Hospital, Saudi Arabia; ag Ministry of Health, Saudi Arabia; ah Foundation for Ambulance Service Development, Emergency Life-Saving Technique Academy of Tokyo, Japan; ai Kyoto University, Japan; aj Kokushikan University Graduate School of Emergency Medical System, Japan; ak Asesores en Emergencias, Mexico; al Emergency medical organizations in Kenya, Kenya; am County Government of Turkana, Kenya; an Kenya Methodist University. Nairobi County, Kenya; ao University hospital of Antwerp, Belgium; ap Western Cape Government Emergency Medical Services, South Africa; aq Netcare. And Resuscitation Council of Southern Africa, South Africa; ar Cape Peninsula University of Technology, Department of Emergency Medical Sciences, Cape Town, South Africa; as University of Doha for Science and Technology, South Africa; at Emergency Médical Service 03, Sahloul University Hospital, Sousse, Tunisia; au Emergency Department of Regional Hospital of Ben Arous, Tunisia Faculty of Medecine of Tunis, Tunis El Manar university, Tunisia; av Service de Pédiatrie et de Néonatologie Hopital Habib Bougatfa de Bizerte- Tunisie, Tunisia; aw Resuscitation Council Sri Lanka, Sri Lanka; ax Sri Lanka Army, Sri Lanka; ay Abu Dhabi Department of Health, United Arab Emirates; az Vice-chair, Department of Emergency Medicine at Southern Philippines Medical Centre, Philippines; ba Resuscitation Committee, Asian Society for Emergency Medicine Manila Doctors Hospital, Philippines; bb Department of Health, Philippines; bc Emergency Medical Service, Public Institution Health Centre ‘Dr. Mustafa Šehović’ and Faculty of Medicine, University of Tuzla, Bosnia and Herzegovina; bd Department of Allied Medical Sciences, Faculty of Applied Medical Sciences, Jordan University of Science and Technology, Irbid, Jordan; be Instituto Nacional Emergência Médica (INEM), Portugal; bf Health School of the Polytechnic Institute of Viseu, Health Sciences Research Unit: Nursing (UICISA: E), Nursing School of Coimbra (ESEnfC), Portugal; bg Department of Emergency Medicine, West China Hospital, Sichuan University, China; bh Department of Emergency Medicine, School of Clinical Medicine, Li Ka Shing Faculty of Medicine, The University of Hong Kong, Hong Kong; bi University of Pécs, Hungary; bj Hungarian Air Ambulance Ltd., Hungary; bk Istanbul Medipol University Anesthesiology and Reanimation Department, Turkey; bl Emergency Disaster Ambulance Physicians Association (AAHD), Turkey; bm Centre Hospitalier de Maubeuge, France; bn Centre Hospitalier Universitaire de Nancy, France; bo Department of Emergency Medicine, College of Medicine, National Taiwan University, Taipei, Taiwan; bp Department of Emergency Medicine, National Taiwan University Hospital Hsinchu Branch, Hsinchu, Taiwan; bq Department of Physiology and Pharmacology, Karolinska Institutet, Stockholm, Department of Perioperative Medicine and Intensive Care, Karolinska University Hospital, Stockholm, Swedish Air Ambulance (SLA), Mora, Rapid Response Car, Capio, Stockholm, Sweden; br Division of Anesthesia and Intensive care, Department of Clinical Science and Education, Karolinska Institutet, Sweden; bs Health Systems & Policy, Department of Global Public Health, Karolinska Institutet, Centre for Clinical Research Sörmland, Uppsala University Department of Medicine, Nyköping Hospital, Sweden; bt Royal Flying Doctor Service of Australia, Australia; bu NSW Ambulance, Australia; bv Australian Capital Territory Ambulance Service, Australia; bw Edith Cowan University, St John Ambulance WA, Australia; bx Hampshire Hospitals NHS Foundation Trust, United Kingdom; by ScotSTAR / Univeristy of Glasgow, Scotland, United Kingdom; bz United Kingdom; ca A.O.U.della Marche Ancona, Italy; cb AUSL della Romagna, Ravenna, Italy; cc Oslo University Hospital, Oslo, Norwegian Air Ambulance, University of Oslo, Oslo, Norway; cd St. Olavs University Hospital, Department of Emergency Medicine and Pre-hospital Services, Trondheim, Norway; ce Ambulance Helicopter Kirkenes, Finnmark Health Trust, Operations- and Intensive Care Department, University Hospital of Northern Norway, Tromsø, Norway; cf RAKOS Stavnger Health Trust Stavanger Norway, Norway; fk Conrad Arnfinn Bjørshol, Senior Researcher, The Regional Centre for Emergency Medical Research and Development (RAKOS), Anaesthesiologist Stavanger University Hospital, Norway; cg Sorlandet Hospital HF/ University of Oslo, Norway; ch Innlandet Hospital, Norway; ci Nordland County General Hospital, Norway; cj Arendal Hospital, Norway; ck Polish Medical Air Rescue, Poland; cl Szpital Czerniakowski Anaesthetic Department, Poland; cm Dept. of Anesthesiology and Intensive Care Medicine, Odense University Hospital Svendborg, Denmark; cn Danish Air Ambulance, Denmark; fo The Prehospital Research Unit, Region of Southern Denmark, Odense University Hospital, Kildemosevej 15, 5000 Odense, Denmark; co Emergency Medical Services, Central Denmark Region, Denmark; cp The Emergenc Debra Perinay standby, The Capital of Denmark, Denmark; cq Regionshospitalet Gødstrup, Denmark; cr Emergency Department Lausanne University Hospital and Lausanne University, Switzerland; cs Division of Emergency Medicine, Department of Anesthesiology, Clinical Pharmacology, Intensive Care and Emergency Medicine, University of Geneva Hospitals and Faculty of Medicine, Geneva, Switzerland; ct Canton Township Fire Department, MI, United States; cu University of Illinois Chicago, United States, Illinios; cv Providence St. Joseph Medical Center, Intermountins, United States; cw University of Michigan, MI, United States; cx Whatcom Couty WA, Dept. of Emergency Medicine, University of Washington, WA, United States; cy Miami Dade Fire Rescue, Core EM Faculty, Jackson Memorial Hospital Emergency Care Center, Faculty of Surgery, University of Miami Miller School of Medicine, South East Florida, United States; cz University of Virginia, United States; da Santa Clara County (CA) Emergency Medical Services Agency, CA, United States; db Beth Israel Deaconess Medical Center Boston, MA, United States; dc Office of the Medical Director Pikes Peak, Southern, & Southeastern Colorado Regions, CO, United States; dd University of Missouri, Central Missouri, United States; de Metropolitan Area EMS Authority, Medstar Mobile, Mobile Healthcare, Fort Worth EM Residency John Peter Smith Hospital, Tarrant county, United States; df Colorado Springs FD, Colorado Springs, United States; dg EMSOA/Lincoln Fire, Lincoln, NE, United States; dh EMS and Beach Safety, City of Hollywood, Florida, United States; di San Juan County EMS, United States; dj Crook County Rire & Rescue, 4 Volunteer Ambulance Agencies in Wilderness Central Oregon, Central Oregon, United States; dk Air Evac Lifeteam, South Texas, United States; dl Department of Emergency Medicine, University of Oklahoma, School of Community Medicine, EMS System for Metropolitan Oklahoma City and Tulsa, Tulsa, OK, United States; dm Alton Memorial EMS System, Alton, Southwest IL, IL, United States; dn University of Tennessee, Health science Center at Memphis Emergency Medicine, Memphis Fire Department, United States; do Township of Neptune New Jersey, NJ, United States; dp UMass Memorial Healthcare, NJ, United States; dq Mississippi Department or Public Safety, United States; dr Madison Emergency Physicians, WI, United States; ds Emergency Medicine Michigan State University College of Human Medicine, KCEMS, MI, United States; dt Emergency Medicine Services, Helsinki University Hospital, and Department of Emergency Medicine, University of Helsinki, Finland; du Emergency Medical Services, Turku University Hospital and University of Turku, Turku, Finland; dv University of Eastern Finland, Centre for prehospital emergency care, Kuopio University Hospital, Kuopio, Finland; dw Medical Director, DRF Stiftung Luftrettung gAG, Filderstadt, Germany; dx DRF Station Halle, Landsberg, Germany; dy Medical Faculty Heidelberg, Department of Anesthesiology, Heidelberg University, Germany; dz Department of Emergency Medicine, VieCuri Medical Center, Venlo, Netherlands; ea Radboud University Medical Center, Netherlands; eb Department of Emergency Medicine, Haaglanden Medical Centre, The Hague, Netherlands; ec Radboud University Medical Centre Nijmegen, Netherlands; ed Department of Anaesthesiology, Erasmus MC University Medical Centre, Rotterdam, Netherlands; ee Ambulance Service Noord, Holland Noord Alkmaar, Netherlands; ef University of Auckland / Auckland Emergency Department, New Zealand; eg Wellington Free Ambulance, AUT University, Auckland, New Zealand; eh Department of Emergency Medicine, Singapore General Hospital, Pre-hospital & Emergency Research Centre, Duke-National University of Singapore Medical School, Centre for Population Health Research and Implementation, SingHealth Regional Health System, Saw Swee Hock School of Public Health, National University of Singapore, Singapore; ei Department of Emergency Medicine, Singapore General Hospital, Singapore; ej Emergency Department, St. Michael’s Hospital Toronto, Ornge, Ontario, Canada; ek Toronto Paramedic Services and Toronto Central Ambulance Communication Centre, Faculty of Medicine, University of Toronto, Canada; el Queen’s University Belfast, Uganda; em University for Business and Technology, Center for Family Medicine - Lipjan, Kosovo; en King Faisal Hospital Rwanda, Rwanda; eo Partners In Health (PIH), Sierra Leone; ep Emergency Department, Mater Dei Hospital, Malta; eq Emergency Department, Mater Dei Hospital, Malta; er Serbian Resuscitation Council, Serbia; es Droning Ingrids hospital, Nuuk, Greenland; et Department of Operations and Intensiv Care Queen Ingrids Hospital, Nuuk, Greenland; eu Rigshospitalet, Capital Region of Denmark, Greenland; ev Mobile Emergency Care Service in Belo Horizonte - Minas Gerais, Brazil; ew Hamad Medical Corporation Ambulance Service Doha, Weill Cornell Medicine-Qatar, Doha, Qatar; ex Philippine Society of Emergency Medical Technicians (PSEMT), Qatar; ey Department of Emergency Medicine, Myongji Hospital Hanyang University College of Medicine, South Korea; ez Emergency Department, Community Healthcare Centre, Emergency Medical Services, Prehospital unit, University of Maribor, Medical faculty, Maribor, Slovenia; fa Center for Intensive Internal Medicine, University Medical Center, Ljubljana, Slovenia; fb Emergency Medical Services, Prehospital Unit, Community Health Center Maribor, Emergency Medical Dispatch Services University Medical Center Ljubljana, Slovenia; fc Medical Intensive Care Unit, University Medical Centre Maribor, Slovenia; fd Department of Emergency Medicine, Faculty of Medicine, Khon Kaen University, Khon Kaen, Thailand; fe HSE National Ambulance Service, University College Cork, University of Galway, Ireland; ff National Ambulance Service Ireland, Ireland; fg Fellow in Prehospital and Disaster Medicine, Head of Prehospital Unit, Emergency and Trauma Department, Hospital Raja Permaisuri Bainun, Ipoh, Malaysia; fh SUMMA 112 Madrid, Spain; fi Prehospital Emergency Services, Navarra Health Services, Founding Partner ABC Saves Lives, Spain; fj Mesa County EMS, CO, United States; a The Prehospital Research Unit, Region of Southern Denmark, Odense University Hospital, Kildemosevej 15, 5000 Odense, Denmark; b Department of Regional Health Research, Region of Southern Denmark, Campusvej 55, 5230 Odense, Denmark; c Department of Anesthesiology and Intensive Care Medicine, University Hospital Kolding, Sygehusvej 24, 6000 Kolding, Denmark; d Falck Denmark, Sydhavnsgade 18, 2450 København SV, Denmark; e Department of Clinical Medicine, University of Copenhagen, Blegdamsvej 3, 2200 Copenhagen, Denmark; f Prehospital Emergency Medical Services, Region Zealand, Ringstedgade 61, 4700 Næstved, Denmark; g ER24, Emergency Management Services, 25 Du Toit Street, Stellenbosch 7600, South Africa; h Division of Emergency Medicine, Department of Medicine, University of Toronto, C. David Naylor Building, 6 Queen’s Park Crescent West, Third Floor, Toronto, ON M5S 3H2, Canada; i Emergency Services, Sunnybrook Health Sciences Center, 2075 Bayview Ave, M4N 3M5 Toronto, Ontario, Canada; j Emergency Medicine, King Abdulaziz University, Jeddah, Saudi Arabia; k Al-Malae’b St, Jeddah 22254, Saudi Arabia

**Keywords:** Health system capacity, Termination of resuscitation (TOR), Emergency care disparities, Health policies

## Abstract

**Background:**

Out-of-hospital cardiac arrest (OHCA) is a major public health problem. This study aims to describe the international variations in the practices related to the initiation, termination, and refraining from resuscitation of adult patients (≥18 years) with a non-traumatic OHCA.

**Methods:**

An exploratory descriptive study was conducted using a cross-sectional online survey. The respondents were recruited using snowball sampling technique. Framework analysis was used to identify key themes in responses, with descriptive statistics summarising data trends.

**Results:**

The study collected responses from 59 countries. Our findings reveal that respondents from 59.3% of countries reported that they initiate resuscitation in all cases where the patients do not show obvious signs of irreversible death or do not have confirmed advance directives. Respondents from 15.3% of countries reported that once started, prehospital resuscitation attempts are not terminated. Prehospitally respondents from 20.3% of the countries reported that they rely exclusively on specific criteria to decide when to terminate resuscitation efforts while in 45.8%, these decisions are made at the discretion of the provider. Respondents from most countries (91.5%) reported that they refrain from resuscitation in the presence of obvious signs of irreversible death. Respondents from 57.6% of countries, reported that they refrained from resuscitation if the patient had a confirmed do-not-attempt-cardiopulmonaryresuscitation (DNACPR), while 15.3% mentioned staff safety as a reason to abstain from attempting resuscitation.

**Conclusion:**

This study reveals global variation in EMS resuscitation practices, reflecting disparities in resources, healthcare infrastructure, EMS system design, community acceptability given cultural and societal norms, and legislation.

## Highlights

• This study reveals extensive global variation in EMS resuscitation practices for OHCA.
• Differences reflect disparities in resources, infrastructure, EMS system design, culture, and legislation.
• Findings highlight the need for context-sensitive, standardised guidelines for initiating and terminating resuscitation.
• Tailored interventions, including bystander and basic life support training, could help improve outcomes.
• Responses from countries without a universal national EMS system may not fully represent nationwide practices, limiting generalizability.

## Introduction

Out-of-hospital cardiac arrest (OHCA) is a global public health concern, affecting an estimated 3.8 million people annually.^1^ Despite efforts to improve outcomes, survival rates of hospital discharge remain low, averaging 9.9% worldwide between 2010 and 2019, with marked regional disparities: Oceania (16.2%), Europe (11.7%), North America (7.7%), and Asia (4.5%).^2^

In 2017, survival rates for hospital discharge showed a 3.6-fold difference across 15 international registries.^3^ The variability in the survival rates probably cannot be attributed solely to the Utstein characteristics.^4^ The numerator and denominator of all estimates for EMS assessed and treated OHCA is inconsistent between registries which obviously may lead to differences in the reported outcomes.^4^ Moreover, as previous studies have highlighted, many system-level, organisational, or contextual factors are not routinely measured or accounted for, yet likely influence survival.^4^, ^5^, ^6^ These may include differences in initiating, terminating, or refraining from resuscitation. Thus, this study aims to describe the international variations in the practices related to the initiation, termination, and refraining from resuscitation of adult patients (≥18 years) with a non-traumatic OHCA.

## Methods

### Study design

This is an exploratory descriptive study that analysed data from a cross-sectional online survey.

### Study setting and population

The study population included members of the European Prehospital Research Alliance (EUPHOREA), the International Liaison Committee of Resuscitation (ILCOR), the European Resuscitation Council (ERC), the National Association of EMS Physicians (NAEMSP), the African Federation for Emergency Medicine (AFEM), the Essential Emergency and Critical Care (EECC) Network, the European Association of EMS (EMS Europe), and the Global Resuscitation Alliance (GRA). See the list of collaborative authors.

### Questionnaire

The questionnaire was developed based on a review of published literature and feedback from members of the EUPHOREA (supplementary file 1). The survey was designed following the CROSS checklist.^7^

To counter response bias a rigorous questionnaire validation process was undertaken. Eight prehospital experts from seven countries acting within the European Prehospital Research Alliance (EUPHOREA) participated in a pre-pilot test, identifying unclear or ambiguous questions, leading to revisions. A modified Delphi process with a panel of ten national prehospital experts was conducted to reach a consensus on the survey's readability.^8^ The final pilot test included 40 international participants recruited by snowball sampling. These responses were used to establish face and criterion validity.^9^ The questionnaire was accessible through REDCap or SurveyXact. To minimize language bias the questionnaire was available in English, French, and Portuguese.

### Data collection

The data collection was conducted from January 2023 to September 2024. The respondents were recruited using snowball sampling of the membership of the collaborating organisations where all respondents were encouraged to recruit others within and external to their organisation.^10^ Participants at the ERC Congress in Barcelona in November 2023 were similarly recruited and encouraged to access and spread the survey link using a QR code.

### Data analysis

REDCap data storage and SurveyXact were used to store data. Descriptive statistics served as the primary method for analysing and presenting the data obtained from the online survey. If there were contradictions between tick-box answers and written responses the written answers were favoured. The free-text responses were analysed using framework analysis. The framework analysis consists of five stages: data familiarisation, framework identification, indexing, charting, and mapping and interpretation.^11^

An inductive and deductive approach allowed various themes to emerge from participants' narratives while drawing insights from the relevant literature.^12^ Through in-depth discussions, the authors critically reviewed the participants' responses, reflected on outliers, and reached a consensus on recurring themes. Once all data had been coded according to the framework analysis the results were summarised into a thematic matrix.

### Ethical considerations

The Danish Health and Medicines Agency (Ref. No. 3-3013-3088/1) and the Research Ethics Committee of the University of Southern Denmark (Ref. No. 23/35945) have approved this project. The Survey complied with all data safety regulations and was approved by the Region of Southern Denmark (Ref. No. 21/23002).

## Results

### The characteristics of the participating countries

**Fig. 1:**
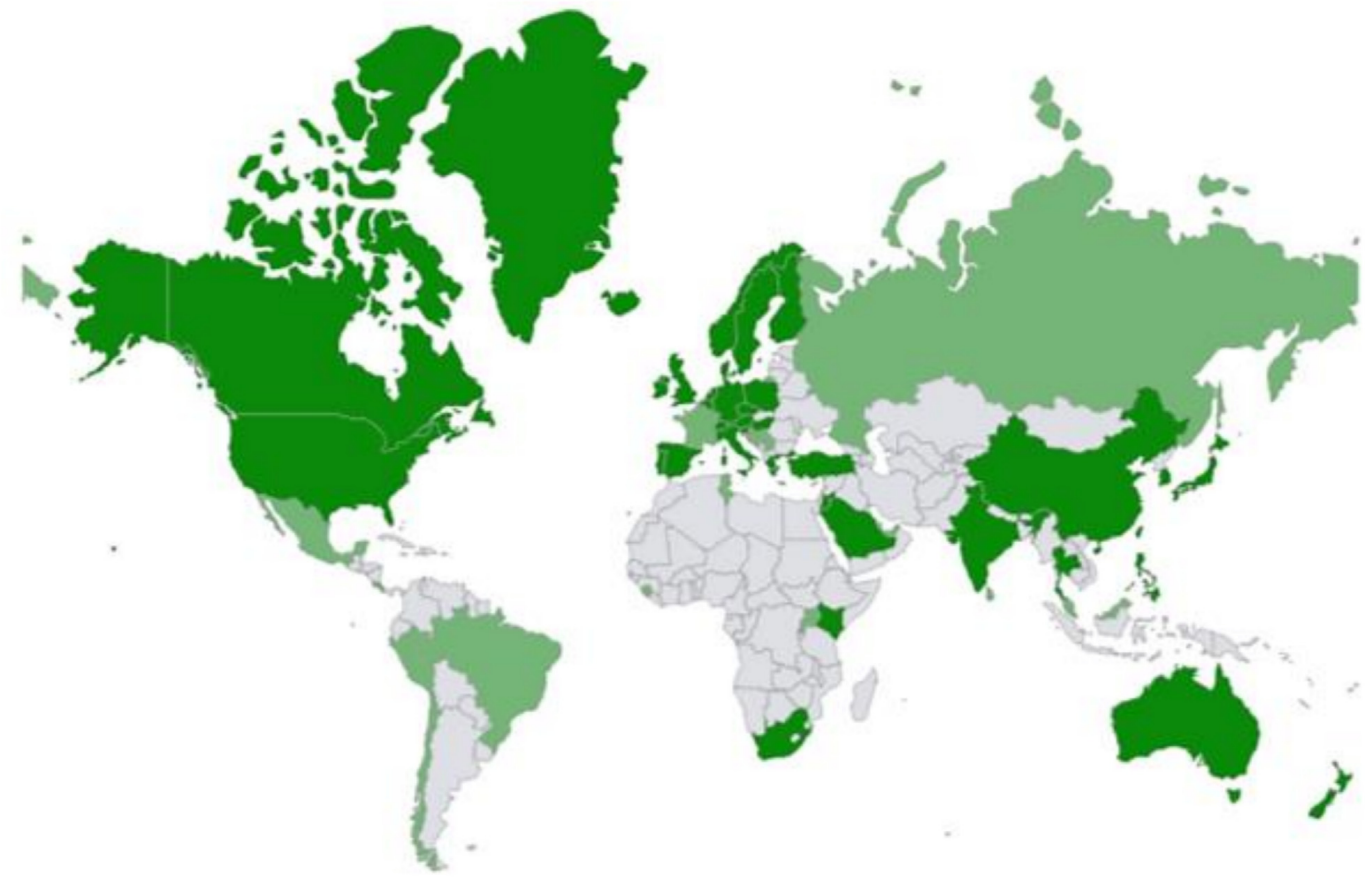
World map of the participating countries.

The study collected 199 complete responses and 124 partial responses from 59 countries. The world map depicting the participating countries is shown in Fig. 1. The snowball sampling method, while useful for reaching a wide audience does not allow the calculation of a response rate. The characteristics of the participating countries are shown in supplementary file 2. In most countries, respondents reported that their EMS system has prehospital physicians available on scene (78%) and/or available by phone (91.5%). In some countries, prehospital physicians are only available on scene and/or by phone in some parts of the country. For example Greenland, at most a nurse or an emergency medical technician (EMT) is available. In Uganda and Rwanda, a nurse is the most competent provider on scene. In Jordan, Qatar, Singapore, the United Arab Emirates, and Taiwan, paramedics are the most competent providers at the scene. In countries, such as Sri Lanka and South Korea, EMTs or firefighters provide on-site care.

Respondents from 5.1% of the countries (Greenland, Sierra Leone, and Uganda) reported that basic life support (BLS) is used, which involves responders providing basic emergency care. This approach is also common in much of South Korea, India, and parts of Africa and China. Prehospital Advanced Life Support (ALS) is more often seen in countries with high resources where highly trained personnel are available to provide a more comprehensive emergency response. In addition, respondents from just over half of the countries (61%) have a program for first responders who are the first to arrive at the emergency scene to provide first aid and care to the patient.

### Initiation of resuscitation

In the framework analysis, three themes concerning the initiation of resuscitation attempts were identified:

• Initiation of resuscitation attempts for all patients
• Initiation of resuscitation attempts of all patients without obvious signs of irreversible death
• Initiation of resuscitation attempts of all patients without a confirmed advance directive

For details of the thematic matrix, see the supplementary file 3 (Initiation of resuscitation).

Respondents from more than half of the countries (59.3%) initiate resuscitation attempts for all patients without obvious signs of irreversible death or when a confirmed advance directive is not at hand. Respondents from almost a third of the countries (30.5%), including nine European countries, six Asian countries, and three African countries, only use obvious signs of irreversible death as a criterion for *not* initiating resuscitation. Kosovo, India, Rwanda, and Peru initiate resuscitation attempts for all patients in cardiac arrest by default. For the division into continents, see Table 1.

**Table 1:** Initiation of resuscitation.

	**Initiation of resuscitation attempts for all patients n (%)**	**Initiation of resuscitation attempts of all patients without obvious signs of irreversible death *n* (%)**	**Initiation of resuscitation attempts of all patients without a confirmed advance directive *n* (%)**
**Africa *n* = 6**	1 (16·7)	5 (83·3)	2 (33·3)
**Asia *n* = 15**	2 (13·3)	13 (86·7)	8 (53·3)
**Central America *n* = 1**	0 (0)	1 (100)	1 (100)
**Europe *n* = 29**	1 (3·5)	28 (96·6)	17 (58·6)
**North America *n* = 6**	0 (0)	6 (100)	6 (100)
**Oceania *n* = 2**	0 (0)	2 (100)	2 (100)
**South America *n* = 3**	1 (33·3)	2 (66·7)	2 (66·7)

Variations in the practice regarding prehospital initiation of resuscitation within individual countries are listed in supplementary file 4. Respondents from nearly half of the countries (49.2%) describe variations in different subpopulations and/or different geographical areas.

### Termination of resuscitation

Three themes concerning the practices regarding termination of resuscitation were identified:

• No termination of resuscitation at scene
• Specific criteria/guidelines (e.g. time specific, guideline, DNACPR)
• Discretion of the care provider

See the thematic matrix in supplementary file 5 (Termination of resuscitation). Respondents from 15.3% of the countries responded that they do not terminate prehospital resuscitation attempts. In contrast, 20.3% report that specific criteria are available to decide when to terminate resuscitation efforts. A larger proportion, 45.8%, report the use of the discretion of the provider for making these decisions. Additionally, respondents from 8.5% of countries apply a combination of both specific criteria and the discretion of the provider, depending on the qualifications and roles of the prehospital personnel involved. Respondents from North America and Oceania reported that they use specific criteria for termination of resuscitation. Respondents from New Zealand reported that paramedics or physicians may remotely make this decision over the phone based on discretion. Respondents from South America, Europe, and Africa reported that they mainly use discretion. Respondents from 13.8% (Greece, Iceland, Ireland, and Italy) of the European countries reported that they, only use specific criteria. In Africa, respondents from one country out of six reported that they use specific criteria. In Asia, the respondents from 53.3% of the participating countries reported that they do not terminate resuscitation at the scene, 26.7% use the discretion of the provider and 20% may terminate resuscitation attempts at the scene following specific criteria. Respondents indicated that in approximately 70% of the countries where specific criteria are used to terminate resuscitation, physicians are present on scene. In contrast, nearly 93% of the countries that rely on provider discretion for termination decisions have physicians on-site. The remaining responding countries using the discretion of the care provider have physicians available by phone, except in Uganda. For the reporting by continent, see Table 2.

**Table 2:** Termination of resuscitation.

	**No termination of resuscitation at scene** ***n* (%)**	**Specific criteria/guidelines (e.g. time specific, guideline, DNACPR)** ***n* (%)**	**Discretion of the provider** ***n* (%)**
**Africa *n* = 6**	1 (16·7)	2 (33·3)	4 (66·7)
**Asia *n* = 15**	8 (53·3)	3 (20)	4 (26·7)
**Central America *n* = 1**	0 (0)	0 (0)	1 (100)
**Europe *n* = 29**	0 (0)	7 (24·1)	22 (75·9)
**North America *n* = 6**	0 (0)	1 (16·7)	6 (100)
**Oceania *n* = 2**	0 (0)	2 (100)	1 (50)
**South America *n* = 3**	0 (0)	0 (0)	2 (66·7)

Variations in the practice of prehospital termination of resuscitation within individual countries are outlined in supplementary file 6. Respondents from most countries (59.3%) report differences across specific subpopulations and/or geographical regions.

### Refraining from resuscitation

Five themes concerning the practices of refraining from resuscitation were identified:

• Patients with obvious signs of irreversible death
• Patients with confirmed DNACPR
• Performing CPR would endanger the lives or safety of the EMS personnel
• Discretion of the care provider
• No refraining from resuscitation attempts in the prehospital setting – all patients are uniformly resuscitated without exception.

See the thematic matrix in supplementary file 7 (Refraining from resuscitation). Table 3 shows the practice regarding refraining from resuscitation in the different countries. Respondents from most countries (91.5%) reported that they refrain from resuscitation in the presence of obvious signs of irreversible death. Respondents from approximately 76% of the countries mention: livor mortis, rigor mortis, decomposition, injuries incompatible with life, and the patient being burned beyond recognition as obvious signs of irreversible death. Respondents from 57.6% of countries reported that they refrained from resuscitation if the patient had a confirmed DNACPR yet only 15.3% mentioned staff safety as a reason to abstain from attempting resuscitation. For the division into countries, see supplementary file 8.

**Table 3:** Refraining from resuscitation.

	**Patients with obvious signs of irreversible death** **n (%)**	**Patients with confirmed DNACPR** **n (%)**	**Performing CPR would endanger the lives or safety of the EMS personnel** **n (%)**	**Discretion of the provider** **n (%)**	**No refraining from resuscitation attempts in the prehospital setting – all patients are uniformly resuscitated without exception** **n (%)**
**Africa *n* = 6**	5 (83·3)	2 (66·7)	2 (66·7)	0 (0)	1 (16·7)
**Asia *n* = 15**	13 (86·7)	8 (53·3)	3 (20)	0 (0)	1 (6·7)
**Central America *n* = 1**	1 (100)	1 (100)	0 (0)	0 (0)	0 (0)
**Europe *n* = 29**	28 (96·6)	17 (58·6)	3 (10·3)	0 (0)	1 (3·4)
**North America *n* = 6**	6 (100)	6 (100)	0 (0)	0 (0)	0 (0)
**Oceania *n* = 2**	2 (100)	2 (100)	1 (50)	0 (0)	0 (0)
**South America *n* = 3**	2 (66·7)	2 (66·7)	0 (0)	2 (66·7)	0 (0)

## Discussion

This study reveals global variation in EMS practices regarding the initiation, termination, and refraining from resuscitation of OHCA. While some countries have prehospital physicians on scene, others rely on paramedics, nurses, firefighters, EMTs, or trained ambulance drivers. BLS is more common in low- and middle-resource systems, whereas ALS is typically used in high-resource settings. Respondents from most countries reported initiating resuscitation unless there were obvious signs of irreversible death or a confirmed advance directive, but approaches vary widely. In some regions, provider safety concerns also influence the decision to refrain from resuscitation. A confirmed advance directive, when considered legally valid or trustworthy, was inconsistently reported to influence termination decisions.

### Comparison with current studies

Studies suggest that EMS-attempted resuscitation rates vary from 40.1% to 66.9% of OHCAs across North America, Europe, and Oceania.^13^, ^14^, ^15^ These variations are attributed to differences in EMS systems, the use of advance directives, and regional cultural practices.^13^, ^14^, ^15^ Similarly, our observations reveal significant regional differences in EMS practices, shaped by system structure, prehospital physician availability, and cultural or regulatory factors. While some countries attempt resuscitation universally, others apply specific criteria or rely on the care provideŕs discretion. These results suggest that EMS practices vary due to resource availability, geographic constraints, and healthcare system development. Low-resource settings, where only nurses or EMTs are available, face other challenges than high-resource settings with on-site or on-call physicians.

There are notable differences between countries that initiate resuscitation in all patients by default (e.g., India, Kosovo) and those that apply specific criteria, such as obvious signs of irreversible death or a confirmed advance directive. These variations may reflect how cultural, ethical, and legal conditions influence decisions on resuscitation initiation. In some countries, the concept of “advance directives” is less prevalent, leading to reliance on discretion by the prehospital care provider or the presence of obvious signs of irreversible death.

The Pan Asian Resuscitation Outcomes Study (PAROS) reported that all OHCA cases were transported to the hospital, unless there were obvious signs of irreversible death, except in Malaysia, where the EMS practices termination of resuscitation in the field.^16^ This corresponds to our findings, where respondents from most Asian countries reported that they do not terminate resuscitation at the scene.

These observed differences may influence survival rates. Studies show that this variability is due to inconsistencies in the definition of the denominator in the calculation of survival rates.^4^, ^5^, ^6^ Our study confirms these variations, which could contribute to substantial global variability in reported outcomes following out-of-hospital cardiac arrest. This study highlights several options for defining a common denominator, such as standardising benchmarks and excluding futile cases. Another possible solution is to report survival rates per 100,000 population instead of percentages, which could better standardise comparisons across populations.

Adjusted for patient and cardiac arrest-related factors, the overall survival to discharge and the likelihood of favourable neurological outcomes are higher in cases where resuscitation was initiated by first responders, largely due to the earlier initiation of CPR.^17^ Our study shows that respondents from most countries in Europe and Oceania reported that they have established citizen first responder programs, whereas only a minority of countries in Asia and Africa have similar programs. This lack of early intervention may contribute to the lower survival rates observed in Asia. A respondent from Hong Kong also mentioned that the low rate of bystander-initiated CPR likely contributes to the limited chances of survival following OHCA. Civil first responder programs could be critical in countries with limited access to professional EMS personnel, where empowering local communities might improve survival outcomes.

### Limitations

Reaching certain parts of the world has been particularly challenging. Regions such as South America, the Baltic countries, and Africa have been underrepresented, with the Western world showing the strongest participation. A previous survey indicated that only about 9% of the African population had access to an EMS system in 2012.^18^ This limited EMS infrastructure likely contributes to the low response rate from Africa. Current geopolitical tensions may also have influenced response rates.

The objective was to obtain at least two responses from each country to ensure data reliability. However, for 15 countries this was not possible, which may impact the data quality for these locations.

Some countries have diverse cultures and medical practices, which could lead to inconsistencies in the responses. Our results may be inaccurate in countries where the couple agencies that are described do not match the vast variation from other agencies across the country. This diversity may introduce variability the study may not fully address. Such variations could impact the generalisability of the findings.

### Global disparities and resource-driven adaptations in EMS organisations

Geographical and subpopulation-specific practices significantly shape EMS organisations. About half of the countries reported regional or population-based differences in initiation and termination of resuscitation, often driven by EMS-system organisation, resource availability, rural–urban divides, or unequal healthcare access. In Uganda, the capital contains over half of the country's healthcare facilities, while rural areas suffer from delayed EMS access due to poor infrastructure, especially in adverse weather. Similarly, Mexico reports stark urban–rural differences with urban areas staffed by paramedics and better healthcare facilities, while rural areas rely primarily on volunteers and basic EMTs. In Thailand, the EMS system reflects geographical variations via categorisation into Advanced and Basic Units. Advanced teams, composed of doctors, nurses, and EMTs, cover urban areas, ensuring higher-level emergency care. In contrast, Basic units, consisting only of EMTs, have a more limited scope of practice. They cover rural and suburban areas, where access to advanced medical resources is inconsistent.

Understanding local contexts is essential for tailoring Emergency Medical Services (EMS) strategies in rural or underserved areas, where limited resources necessitate alternative approaches compared to urban centres with robust healthcare infrastructures. The European Resuscitation Council’s 2021 guidelines highlight the need for a list of essential resuscitation resources adapted to low-resource settings.^19^ The standard of care in high-resource environments is often unattainable in low-resource areas due to financial constraints, limited infrastructure, logistical challenges, and a shortage of qualified personnel.^20^ Despite these realities, international progress in addressing resuscitation disparities has been slow.^20^ Acknowledging these differences could stimulate the development of localised guidelines and promote the involvement of representatives from low-resource settings in shaping global standards. Our findings support this by providing insight into systemic variability. ILCOR’s new concept of the Chainmail of Survival introduces a flexible framework for resuscitation systems, care, and research that can be adapted to low-resource settings. Its first consensus statement on low-resource resuscitation is a step forward.^20^ There are no universal solutions to improve OHCA survival; interventions should be adapted to local, regional, or even national practices. The insights offered in this study can inform guidelines that, may assist in ensuring broader access to quality resuscitation care. In addition, the World Health Organization’s Acute Care Action Network (WHO ACAN) provides a platform for collaboration and knowledge sharing to strengthen emergency and acute care systems globally, particularly in low-resource settings.^21^ By integrating the Chainmail of Survival framework with the principles promoted by WHO ACAN, there is an opportunity to enhance the development of context-appropriate strategies. WHO ACAN’s initiatives further align with efforts to create equitable access to life-saving interventions, supporting the global agenda for improving outcomes in acute care settings.^21^

This study underscores the importance of cross-country comparisons and shared learning. By examining EMS variation, countries can identify modifiable targets for improvement. For instance, expanding first responder programs in regions with limited access to professional EMS personnel or remote physician decision-making could lead to improved survival rates following OHCA. Understanding these variations and their underlying causes provides critical insights into how global EMS systems can be optimised to enhance patient outcomes.

## Conclusion

This study reveals global variation in EMS resuscitation practices, reflecting disparities in resources, healthcare infrastructure, EMS system design, community acceptability given cultural and societal norms, and legislation. The findings emphasise the complexity and diversity of pre-hospital resuscitation worldwide, pointing to the need for tailored interventions. Efforts such as enhancing bystander and basic life support training and establishing clear, standardised guidelines for initiating and discontinuing resuscitation −adapted to cultural contexts − could improve outcomes. Standardising these practices at the national or regional level, while respecting community norms, may ultimately enhance the effectiveness of EMS systems, and improve patient survival rates.

## CRediT authorship contribution statement

**Jeannett Kjær:** Writing – original draft, Visualization, Project administration, Methodology, Investigation, Formal analysis. **Louise Milling:** Writing – review & editing, Methodology, Investigation. **Anne Craveiro Brøchner:** Writing – review & editing, Validation, Supervision, Investigation. **Freddy Lippert:** Writing – review & editing, Validation, Investigation. **Stig Nikolaj Blomberg:** Writing – review & editing, Validation, Investigation. **Helle Collatz Christensen:** Writing – review & editing, Validation, Supervision, Investigation. **Robyn Holgate:** Writing – review & editing, Validation, Investigation. **Laurie J. Morrison:** Writing – review & editing, Validation, Supervision, Investigation. **Abdullah Bakhsh:** Writing – review & editing, Validation, Investigation. **Søren Mikkelsen:** Writing – review & editing, Validation, Supervision, Project administration, Methodology, Investigation, Formal analysis.

## Declaration of competing interest

The authors declare the following financial interests/personal relationships which may be considered as potential competing interests: One of the authors serves in a leadership role as a member of the Board of Directors for the European Association of EMS (unpaid) and is a member of the Executive Board of the Global Resuscitation Alliance (unpaid). Additionally, one of the authors participates in Data Safety Monitoring Boards and advisory boards, including protocol review committees for various trials and networks funded by the NIH, as well as a DSMB for a European trial. All of these roles are unrelated to the scope of this work.
