# Supplementary material for: Prehospital cardiac arrest resuscitation practices differ around the globe
Source: Resusc Plus. 2025 Jun 24;25:101017. doi: 10.1016/j.resplu.2025.101017 (PMC12329125; doi:10.1016/j.resplu.2025.101017)
Supplement: Supplementary Data 1 [file mmc1.docx]

**Supplementary Materials**

| Supplementary file 1 – Questionnaire |
| --- |
| Supplementary file 2 – Characteristics of the participating countries |
| Supplementary file 3 – Framework matrix (initiation of resuscitation) |
| Supplementary file 4 – Table - initiation of resuscitation |
| Supplementary file 5 – Framework matrix (termination of resuscitation) |
| Supplementary file 6 – Table - termination of resuscitation |
| Supplementary file 7 – Framework matrix (refraining from resuscitation) |
| Supplementary file 8 – Table - refraining from resuscitation |

**Supplementary file 1 – Questionnaire**

**Do you agree to be listed as a collaborator in all publications arising from this survey?
You can always withdraw your consent**

(1)  Yes

(0)  No

**May we contact you by email if we have further questions about your answers?**

(1)  Yes

(0)  No

**Email**

_____

**Please provide full name, email, and affiliation.
(Your answers will be anonymized in the article).**

________________________________________
________________________________________
________________________________________
________________________________________
________________________________________
________________________________________

General information

**Which country do your answers refer to?**

(1)  Afghanistan

(2)  Albania

(3)  Algeria

(4)  American Samoa

(5)  Andorra

(6)  Angola

(7)  Anguilla

(8)  Antarctica

(9)  Antigua and Barbuda

(10)  Argentina

(11)  Armenia

(12)  Aruba

(13)  Australia

(14)  Austria

(15)  Azerbaijan

(16)  Bahamas

(17)  Bahrain

(18)  Bangladesh

(19)  Barbados

(20)  Belarus

(21)  Belgium

(22)  Belize

(23)  Benin

(24)  Bermuda

(25)  Bhutan

(26)  Bolivia

(27)  Bosnia and Herzegovina

(28)  Botswana

(29)  Bouvet Island

(30)  Brazil

(31)  British Indian Ocean Territory

(32)  British Virgin Islands

(33)  Brunei

(34)  Bulgaria

(35)  Burkina Faso

(36)  Burma

(37)  Burundi

(38)  Cambodia

(39)  Cameroon

(40)  Canada

(41)  Cape Verde

(42)  Cayman Islands

(43)  Central African Republic

(44)  Chad

(45)  Chile

(46)  China

(47)  Christmas Island

(48)  Cocos (Keeling) Islands

(49)  Colombia

(50)  Comoros

(51)  Congo

(52)  Cook Islands

(53)  Costa Rica

(54)  Cote d'Ivoire

(55)  Croatia

(56)  Cuba

(57)  Curacao

(58)  Cyprus

(59)  Czech Republic

(60)  Denmark

(61)  Djibouti

(62)  Dominica

(63)  Dominican Republic

(64)  Ecuador

(65)  Egypt

(66)  El Salvador

(67)  Equatorial Guinea

(68)  Eritrea

(69)  Estonia

(70)  Ethiopia

(71)  Falkland Islands (Islas Malvinas)

(72)  Faroe Islands

(73)  Fiji

(74)  Finland

(75)  France

(77)  French Guiana

(78)  French Polynesia

(79)  French Southern and Antarctic Lands

(80)  Gabon

(81)  Gambia

(82)  Gaza Strip

(83)  Georgia

(84)  Germany

(85)  Ghana

(86)  Gibraltar

(87)  Greece

(88)  Greenland

(89)  Grenada

(90)  Guadeloupe

(91)  Guam

(92)  Guatemala

(93)  Guernsey

(94)  Guinea

(95)  Guinea-Bissau

(96)  Guyana

(97)  Haiti

(98)  Heard Island and McDonald Islands

(99)  Holy See (Vatican City)

(100)  Honduras

(101)  Hong Kong (SAR China)

(102)  Hungary

(103)  Iceland

(104)  India

(105)  Indonesia

(106)  Iran

(107)  Iraq

(108)  Ireland

(109)  Isle of Man

(110)  Israel

(111)  Italy

(112)  Jamaica

(113)  Japan

(114)  Jersey

(115)  Jordan

(116)  Kazakhstan

(117)  Kenya

(118)  Kiribati

(119)  Kosovo

(120)  Kuwait

(121)  Kyrgyzstan

(122)  Laos

(123)  Latvia

(124)  Lebanon

(125)  Lesotho

(126)  Liberia

(127)  Libya

(128)  Liechtenstein

(129)  Lithuania

(130)  Luxembourg

(131)  Macau (SAR China)

(132)  Macedonia

(133)  Madagascar

(134)  Malawi

(135)  Malaysia

(136)  Maldives

(137)  Mali

(138)  Malta

(139)  Marshall Islands

(140)  Martinique

(141)  Mauritania

(142)  Mauritius

(143)  Mayotte

(144)  Mexico

(145)  Micronesia Federated States of

(146)  Moldova

(147)  Monaco

(148)  Mongolia

(149)  Montenegro

(150)  Montserrat

(151)  Morocco

(152)  Mozambique

(153)  Namibia

(154)  Nauru

(155)  Nepal

(156)  Netherlands

(157)  New Caledonia

(158)  New Zealand

(159)  Nicaragua

(160)  Niger

(161)  Nigeria

(162)  Niue

(163)  Norfolk Island

(164)  Northern Mariana Islands

(165)  Norway

(166)  Oman

(167)  Pakistan

(168)  Palau

(169)  Panama

(170)  Papua New Guinea

(171)  Paraguay

(172)  Peru

(173)  Philippines

(174)  Pitcairn Islands

(175)  Poland

(176)  Portugal

(177)  Puerto Rico

(178)  Qatar

(179)  Reunion

(180)  Romania

(181)  Russia

(182)  Rwanda

(183)  Saint Barthelemy

(184)  Saint Helena and Ascension and Tristan da Cunha

(185)  Saint Kitts and Nevis

(186)  Saint Lucia

(187)  Saint Martin

(188)  Saint Pierre and Miquelon

(189)  Saint Vincent and the Grenadines

(190)  Samoa

(191)  San Marino

(192)  Sao Tome and Principe

(193)  Saudi Arabia

(194)  Senegal

(195)  Serbia

(196)  Seychelles

(197)  Sierra Leone

(198)  Singapore

(199)  Sint Maarten

(200)  Slovakia

(201)  Slovenia

(202)  Solomon Islands

(203)  Somalia

(204)  South Africa

(205)  South Georgia and the Islands

(248)  South Korea

(206)  South Sudan

(207)  Spain

(208)  Sri Lanka

(209)  Sudan

(210)  Suriname

(211)  Svalbard

(212)  Swaziland

(213)  Sweden

(214)  Switzerland

(215)  Syria

(216)  Taiwan

(217)  Tajikistan

(218)  Tanzania

(219)  Thailand

(220)  Timor-Leste

(221)  Togo

(222)  Tokelau

(223)  Tonga

(224)  Trinidad and Tobago

(225)  Tunisia

(226)  Turkey

(227)  Turkmenistan

(228)  Turks and Caicos Islands

(229)  Tuvalu

(230)  Uganda

(231)  Ukraine

(232)  United Arab Emirates

(233)  United Kingdom

(234)  United States

(235)  United States Minor Outlying Islands

(236)  Uruguay

(237)  Uzbekistan

(238)  Vanuatu

(239)  Venezuela

(240)  Vietnam

(241)  Virgin Islands

(242)  Wallis and Futuna

(243)  West Bank

(244)  Western Sahara

(245)  Yemen

(246)  Zambia

(247)  Zimbabwe

**Do your answers refer to a specific region in your country or to the entire country?
If a specific region, please specify.**

(2)  The entire country

(1)  A specific region in my country _____

**What is your position in your EMS system?**

(1)  Medical Director

(2)  Administrative personnel

(3)  Paramedic

(4)  Prehospital physician

(99)  Other, state _____

**Please choose the population size your EMS system cover:**

(1)  0-499,999

(2)  500,000-999,999

(3)  1,000,000-1,999,999

(4)  2,000,000-2,999,999

(5)  3,000,000-4,999,999

(6)  5,000,000-10,000,000

(7)  More than 10,000,000

(8)  Don't know

**What is the approximate number of cardiac arrests handled by your EMS system annually?**

(1)  0-499

(2)  500-999

(3)  1000-1999

(4)  2000-2999

(5)  3000-3999

(6)  4000-4999

(7)  5000-10,000

(8)  >10,000

(9)  Don't know

**If possible, please state the incidence of cardiac arrests per 100,000 inhabitants handled by your EMS system annually.**

(1)  0-24 per 100,000 inhabitants

(2)  25-49 per 100,000 inhabitants

(3)  50-74 per 100,000 inhabitants

(4)  75-99 per 100,000 inhabitants

(5)  100-150 per 100,000 inhabitants

(6)  More than 150 per 100,000 inhabitants

(7)  Don't know

**What prehospital healthcare professionals are available in your EMS system?
Feel free to choose more than one option.**

(1)  Volunteers

(2)  Firemen

(3)  Emergency medical technician

(4)  Paramedic

(5)  Nurse

(6)  Physician (on-scene)

(7)  Physician (avaliable by phone)

(99)  Other, state _____

(8)  Don't know

**What does your EMS system provide?
Feel free to choose more than one option.**

(1)  Basic life support (BLS)

(2)  Advanced life support (ALS)

(3)  A citizen first responder program for cardiac arrest

(4)  Don't know

**Who is the highest-ranking prehospital healthcare professional on-scene in your EMS system?**

(1)  Physician (specialist)

(2)  Physician

(3)  Paramedic

(4)  Emergency medical technician

(5)  Nurse

(6)  Police- or firemen

(99)  Other, state _____

(7)  Don't know

**Who is the highest-ranking prehospital healthcare professional available by phone in your EMS system?**

(8)  No personnel available by phone

(1)  Physician (specialist)

(2)  Physician

(3)  Paramedic

(4)  Emergency medical technician

(5)  Nurse

(6)  Police- or firemen

(99)  Other

(9)  Don't know

Prehospital initiation of resuscitation

**What determines the practice regarding prehospital initiation of resuscitation in your EMS system?
Feel free to choose more than one option.**

(1)  Standardized protocols

(2)  Rules

(3)  Guidelines

(4)  Medical directives

(5)  Online medical control

(6)  Decision-making without the use of guidelines or protocols

(7)  On-site medical control

(99)  Other, state _____

(8)  Don't know

**Please describe the practice regarding prehospital initiation of resuscitation in your EMS system.**

________________________________________
________________________________________
________________________________________
________________________________________
________________________________________
________________________________________

**If possible, please provide documentation about the practice regarding the initiation of resuscitation in your EMS system.
E.g. guidance document, an accessible reference, law text, or website.
You can upload a pdf-file below.**

________________________________________
________________________________________
________________________________________
________________________________________
________________________________________
________________________________________

**You can upload a document as a pdf-file.**



**Are there variations in the practice regarding prehospital initiation of resuscitation in different geographical areas of your EMS system?**

(1)  Yes

(2)  No

(3)  Don't know

**What do you think is the reason for these geographical variations in the practice regarding prehospital initiation of resuscitation in your EMS system?
Feel free to choose more than one option.**

(1)  EMS-system organization

(2)  Response time

(3)  Differences in work-flow

(4)  Differences in treatment facilities

(99)  Other, state _____

(6)  Don't know

**Please describe the geographical variations in the practice regarding prehospital initiation of resuscitation in your EMS system.**

________________________________________
________________________________________
________________________________________
________________________________________
________________________________________
________________________________________

Prehospital termination of resuscitation

**What determines the practice regarding prehospital termination of resuscitation in your EMS system?
Feel free to choose more than one option.**

(1)  Standardized protocols

(2)  Rules

(3)  Guidelines

(4)  Medical directives

(5)  Online medical control

(6)  Decision-making without the use of guidelines or protocols

(7)  On-site medical control

(8)  We don't terminate resuscitation in the prehospital setting

(99)  Other, state _____

(9)  Don't know

**Please describe the practice regarding prehospital termination of resuscitation in your EMS system.**

________________________________________
________________________________________
________________________________________
________________________________________
________________________________________
________________________________________

**If possible, please provide documentation about the practice regarding the termination of resuscitation in your EMS system.
E.g. guidance document, an accessible reference, law text, or website.
You can upload a pdf-file below.**

________________________________________
________________________________________
________________________________________
________________________________________
________________________________________
________________________________________

**You can upload a document as a pdf-file.**



**Who has the authority to decide to terminate prehospital resuscitation in your EMS system?
Feel free to choose more than one option.**

(1)  Physician (specialist) on scene

(2)  Physician (specialist) via telephone

(3)  Physician on scene

(4)  Physician at a hospital

(5)  Paramedic

(6)  Emergency medical technician

(7)  Nurse

(8)  Police- or firemen

(9)  Laymen

(10)  We don't terminate resuscitation in the prehospital setting

(99)  Other, state _____

(11)  Don't know

**Are there variations in the practice regarding prehospital termination of resuscitation in different geographical areas of your EMS system?**

(1)  Yes

(2)  No

(3)  Don't know

**What do you think is the reason for these geographical variations in the practice regarding prehospital termination of resuscitation in your EMS system?
Feel free to choose more than one option.**

(1)  EMS-system organization

(2)  Response time

(3)  Differences in work-flow

(4)  Differences in treatment facilities

(99)  Other, state _____

(6)  Don't know

**Please describe the geographical variations in the practice regarding prehospital termination of resuscitation in your EMS system.**

________________________________________
________________________________________
________________________________________
________________________________________
________________________________________
________________________________________

**Are there variations in prehospital termination of resuscitation criteria for subpopulations in your EMS system?
Feel free to choose more than one option.**

(6)  There are no variations

(1)  In patients eligible for organ donation

(2)  In patients eligible for extracorporeal cardiopulmonary resuscitation (eCPR)

(3)  In patients from nursing homes

(4)  In patients from different geographical locations

(5)  For specific age groups

(99)  Other, state _____

(7)  Don't know

**Please describe the variations in termination of resuscitation criteria in the subpopulation in your EMS system.**

________________________________________
________________________________________
________________________________________
________________________________________
________________________________________
________________________________________

Prehospital refraining from resuscitation

**What determines the practice regarding prehospital refraining from resuscitation in your EMS system?
Feel free to choose more than one option.**

(1)  Standardized protocols

(2)  Rules

(3)  Guidelines

(4)  Medical directives

(5)  Online medical control

(6)  Decision-making without the use of guidelines or protocols

(7)  On-site medical control

(8)  We don't refrain from resuscitation in the prehospital setting

(99)  Other, state _____

(9)  Don't know

**Please describe the practice regarding prehospital refraining from resuscitation in your EMS system.**

________________________________________
________________________________________
________________________________________
________________________________________
________________________________________
________________________________________

**If possible, please provide documentation about the practice regarding the refraining from resuscitation in your EMS system.
E.g. guidance document, an accessible reference, law text, or website.
You can upload a pdf-file below.**

________________________________________
________________________________________
________________________________________
________________________________________
________________________________________
________________________________________

**You can upload a document as a pdf-file.**



**Who has the authority to decide to refrain from prehospital resuscitation in your EMS system?
Feel free to choose more than one option.**

(1)  Physician (specialist) on scene

(2)  Physician (specialist) via telephone

(3)  Physician on scene

(4)  Physician at a hospital

(5)  Paramedic

(6)  Emergency medical technician

(7)  Nurse

(8)  Police- or firemen

(9)  Laymen

(10)  We don't refrain from resuscitation in the prehospital setting

(99)  Other, state _____

(11)  Don't know

**Do do-not-attempt-cardiopulmonary-resuscitation (DNACPR) orders or similar exist in your EMS system?**

(1)  Yes

(2)  No

(3)  Don't know

**Are prehospital healthcare professionals legally obliged to adhere to a do-not-attempt-cardiopulmonary-resuscitation (DNACPR) in your EMS system?**

(1)  Yes

(0)  No

(2)  Don't know

**Can prehospital healthcare professionals legally refrain from resuscitation if obvious clinical signs of irreversible death are present?**

(1)  Yes

(2)  No

(3)  Don't know

**Please choose the obvious clinical signs of irreversible death in your EMS system.
Feel free to choose more than one option.**

(1)  Livor Mortis: lividity or pooling of blood in dependent parts of the body

(2)  Rigor Mortis: hardening of the muscles or rigidity

(3)  Decomposition: cadaverositas

(4)  Injuries incompatible with life: these include decapitation or transection

(5)  Burned beyond recognition

(99)  Other, state _____

(6)  Don't know

**Any final comments or suggestions?**

________________________________________
________________________________________
________________________________________
________________________________________

**Supplementary file 2 – Characteristics of the participating countries**

| Country | n | Available healthcare professionals | Level of prehospital care | The most competent provider on-scene | The most competent provider available by phone |
| --- | --- | --- | --- | --- | --- |
| Africa | | | | | |
| Kenya | 3 |  |  |  | Physician (specialist) |
|  |  | Volunteers, | ALS, | Physician* |  |
|  |  | Firemen, | First responder program | Paramedic/nurse |  |
|  |  | Emergency medical technician, |  |  |  |
|  |  | Paramedic, |  |  |  |
|  |  | Nurse, |  |  |  |
|  |  | Physician (on-scene), |  |  |  |
| Rwanda | 1 | Physician (available by phone) | ALS | Nurse | Physician |
|  |  | Volunteers, |  |  |  |
|  |  | Firemen, |  |  |  |
|  |  | Emergency medical technician, |  |  |  |
|  |  | Paramedic, |  |  |  |
|  |  | Nurse, |  |  |  |
| Sierra Leone | 1 | Physician (available by phone) | BLS | Physician (specialist) | Physician (specialist) |
|  |  | Volunteers, |  |  |  |
|  |  | Paramedic, |  |  |  |
|  |  | Nurse, |  |  |  |
|  |  | Physician (on-scene) |  |  |  |
| South Africa | 4 | Physician (available by phone) |  | Physician (specialist) | Physician (specialist) |
|  |  | Emergency Care Practitioner, | ALS, |  |  |
|  |  | Volunteers, | First responder program |  |  |
|  |  | Firemen, |  |  |  |
|  |  | Emergency medical technician, |  |  |  |
|  |  | Paramedic, |  |  |  |
|  |  | Nurse*, |  |  |  |
|  |  | Physician (on-scene), |  |  |  |
| Tunisia | 4 | Physician (available by phone) |  | Physician (specialist) | Physician (specialist) |
|  |  | Firemen, | ALS |  |  |
|  |  | Emergency medical technician, | First responder program* |  |  |
|  |  | Paramedic, |  |  |  |
|  |  | Nurse, |  |  |  |
|  |  | Physician (on-scene), |  |  |  |
| Uganda | 1 | Physician (available by phone) | BLS | Nurse | Don't know |
|  |  | Volunteers, |  |  |  |
|  |  | Paramedic, |  |  |  |
|  |  | Nurse |  |  |  |
| Asia | | | | | |
| China | 4 |  |  |  |  |
|  |  | Volunteers*, | BLS, | Physician (specialist) – in some parts of the country | Physician (specialist) - in some parts of the country, |
|  |  | Firemen, | ALS* | Paramedic | Paramedic |
|  |  | Emergency medical technician*, |  |  |  |
|  |  | Paramedic, |  |  |  |
|  |  | Nurse, |  |  |  |
|  |  | Physician (on-scene), |  |  |  |
| India | 2 | Physician (available by phone)* |  |  | Paramedic* |
|  |  | Volunteers*, | BLS, | Physician – in some parts of the country |  |
|  |  | Emergency medical technician*, | ALS* | Paramedic |  |
|  |  | Paramedic, |  |  |  |
|  |  | Nurse*, |  |  |  |
| Japan | 3 | Physician (on-scene) – in some parts of the country |  |  | Physician (specialist) |
|  |  | Volunteers*, | ALS, | Physician (specialist) – in some parts of the country |  |
|  |  | Firemen, | First responder program | Paramedic |  |
|  |  | Emergency medical technician, |  |  |  |
|  |  | Paramedic, |  |  |  |
|  |  | Nurse*, |  |  |  |
|  |  | Physician (on-scene), |  |  |  |
| Jordan | 2 | Physician (available by phone) | ALS | Paramedic |  |
|  |  | Emergency medical technician, |  |  | No personnel available by phone*, |
|  |  | Paramedic, |  |  | Physician - in some parts of the country |
|  |  | Nurse* |  |  |  |
| Malaysia | 1 | Physician (available by phone) – in some parts of the country | ALS | Physician (specialist) | Physician (specialist) |
|  |  | Volunteers, |  |  |  |
|  |  | Firemen, |  |  |  |
|  |  | Paramedic, |  |  |  |
|  |  | Nurse, |  |  |  |
|  |  | Physician (on-scene), |  |  |  |
| Philippines | 3 | Physician (available by phone) |  | Physician - in some parts of the country | Physician (specialist) |
|  |  | Volunteers, | ALS, |  |  |
|  |  | Firemen, | First responder program* |  |  |
|  |  | Emergency medical technician, |  |  |  |
|  |  | Paramedic*, |  |  |  |
|  |  | Nurse, |  |  |  |
|  |  | Physician (on-scene), |  |  |  |
| Qatar | 2 | Physician (available by phone) | ALS | Critical Care Paramedic | Physician (specialist) |
|  |  | Emergency medical technician, |  |  |  |
|  |  | Paramedic, |  |  |  |
|  |  | Critical care paramedic, |  |  |  |
|  |  | Nurse, |  |  |  |
| Saudi Arabia | 5 | Physician (available by phone) |  |  | Physician (specialist) |
|  |  | Volunteers, | ALS, | Physician (specialist) – in some parts of the country |  |
|  |  | Firemen*, | First responder program | Paramedic |  |
|  |  | Emergency medical technician, |  |  |  |
|  |  | Paramedic, |  |  |  |
|  |  | Nurse*, |  |  |  |
|  |  | Physician (on-scene), |  |  |  |
| Singapore | 2 | Physician (available by phone) |  | Paramedic | Physician (specialist) |
|  |  | Firemen, | ALS, |  |  |
|  |  | Emergency medical technician, | First responder program |  |  |
|  |  | Paramedic, |  |  |  |
|  |  | Nurse*, |  |  |  |
| South Korea | 2 | Physician (available by phone) |  |  | Physician (specialist) |
|  |  | Firemen, | ALS* | Physician (on-scene) - in some parts of the country, |  |
|  |  | Emergency medical technician, | BLS | Emergency medical technician |  |
|  |  | Nurse, | First responder program* |  |  |
|  |  | Physician (on-scene) - in some parts of the country, |  |  |  |
| Sri Lanka | 2 | Physician (available by phone) |  | Emergency medical technician | Physician (specialist) |
|  |  | Emergency medical technician | ALS* |  |  |
| Taiwan | 2 | Physician (available by phone) - in some parts of the country | BLS  ALS | Paramedic | Physician (specialist)* |
|  |  | Volunteers, |  |  |  |
|  |  | Firemen, |  |  |  |
|  |  | Emergency medical technician, |  |  |  |
|  |  | Paramedic, |  |  |  |
|  |  | Nurse*, |  |  |  |
| Thailand | 2 | Physician (available by phone) |  |  | Physician (specialist) |
|  |  | Volunteers*, | ALS, | Physician (specialist)* |  |
|  |  | Emergency medical technician, | First responder program* | Physician* |  |
|  |  | Paramedic*, |  |  |  |
|  |  | Nurse, |  |  |  |
|  |  | Physician (on-scene), |  |  |  |
| Turkey | 2 | Physician (available by phone) | ALS | Physician | Physician |
|  |  | Emergency medical technician, |  |  |  |
|  |  | Paramedic, |  |  |  |
|  |  | Nurse*, |  |  |  |
|  |  | Physician (on-scene) |  |  |  |
| United Arab Emirates | 1 | Physician (available by phone) | ALS | Paramedic | Physician |
|  |  | Volunteers, |  |  |  |
|  |  | Emergency medical technician, |  |  |  |
|  |  | Paramedic, |  |  |  |
|  |  | Nurse, |  |  |  |
|  |  | Physician (available by phone) |  |  |  |
| Central America | | | | | |
| Costa Rica | 1 |  | ALS | Paramedic | Physician (specialist) |
|  |  | Volunteers, |  |  |  |
|  |  | Emergency medical technician, |  |  |  |
|  |  | Paramedic, |  |  |  |
|  |  | Physician (available by phone) |  |  |  |
| Europa | | | | | |
| Austria | 3 |  |  | Physician (specialist) | Physician (specialist)* |
|  |  | Volunteers, | ALS, |  |  |
|  |  | Firemen, | First responder program |  |  |
|  |  | Emergency medical technician, |  |  |  |
|  |  | Paramedic, |  |  |  |
|  |  | Physician (on-scene), |  |  |  |
| Belgium | 2 | Physician (available by phone) |  | Physician (specialist) | Physician (specialist) |
|  |  | Volunteers, | ALS, |  |  |
|  |  | Firemen, | First responder program |  |  |
|  |  | Emergency medical technician, |  |  |  |
|  |  | Paramedic, |  |  |  |
|  |  | Nurse, |  |  |  |
|  |  | Physician (on-scene), |  |  |  |
| Bosnia and Herzegovina | 1 | Physician (available by phone) |  | Physician (specialist) | Physician (specialist) |
|  |  | Emergency medical technician, | ALS, |  |  |
|  |  | Nurse, | First responder program |  |  |
|  |  | Physician (on-scene), |  |  |  |
| Croatia | 1 | Physician (available by phone) | ALS | Physician (specialist) | Physician (specialist) |
|  |  | EMS educated drivers, |  |  |  |
|  |  | Emergency medical technician, |  |  |  |
|  |  | Physician (on-scene) |  |  |  |
| Czech Republic | 6 | Physician (available by phone) |  | Physician (specialist) | Physician (specialist) |
|  |  | Volunteers, | ALS, |  |  |
|  |  | Firemen*, | First responder program |  |  |
|  |  | Emergency medical technician, |  |  |  |
|  |  | Paramedic, |  |  |  |
|  |  | Nurse, |  |  |  |
|  |  | Physician (on-scene), |  |  |  |
| Denmark | 9 | Physician (available by phone) |  | Physician (specialist) | Physician (specialist) |
|  |  | Volunteers, | ALS, |  |  |
|  |  | Firemen, | First responder program |  |  |
|  |  | Emergency medical technician, |  |  |  |
|  |  | Paramedic, |  |  |  |
|  |  | Physician (on-scene), |  |  |  |
| Finland | 5 | Physician (available by phone) |  | Physician (specialist) | Physician (specialist) |
|  |  | Volunteers, | ALS, |  |  |
|  |  | Firemen, | First responder program* |  |  |
|  |  | Emergency medical technician*, |  |  |  |
|  |  | Paramedic*, |  |  |  |
|  |  | Nurse, |  |  |  |
|  |  | Physician (on-scene), |  |  |  |
| France | 2 | Physician (available by phone)* |  | Physician (specialist) | Physician (specialist) |
|  |  | Firemen, | ALS |  |  |
|  |  | Emergency medical technician, | First responder program* |  |  |
|  |  | Paramedic, |  |  |  |
|  |  | Nurse, |  |  |  |
|  |  | Physician (on-scene), |  |  |  |
| Germany | 4 | Physician (available by phone) |  | Physician (specialist) | Physician (specialist) |
|  |  | Firemen*, | ALS, |  |  |
|  |  | Emergency medical technician, | First responder program |  |  |
|  |  | Paramedic, |  |  |  |
|  |  | Physician (on-scene), |  |  |  |
| Greece | 3 | Physician (available by phone) |  | Physician (specialist) | Physician (specialist) |
|  |  | Emergency medical technician, | ALS, |  |  |
|  |  | Paramedic*, | First responder program* |  |  |
|  |  | Nurse*, |  |  |  |
|  |  | Physician (on-scene), |  |  |  |
| Greenland | 3 | Physician (available by phone) |  |  | Physician |
|  |  | Hospital porter*, | BLS, | Emergency medical technician*, |  |
|  |  | Volunteers*, | First responder program* | Nurse* |  |
|  |  | Firemen, |  |  |  |
|  |  | Emergency medical technician*, |  |  |  |
|  |  | Nurse*, |  |  |  |
| Hungary | 3 | Physician (available by phone)* |  | Physician (specialist) | Physician (specialist) |
|  |  | Volunteers*, | ALS, |  |  |
|  |  | Firemen, | First responder program* |  |  |
|  |  | Emergency medical technician, |  |  |  |
|  |  | Paramedic, |  |  |  |
|  |  | Nurse*, |  |  |  |
|  |  | Physician (on-scene), |  |  |  |
| Iceland | 2 | Physician (available by phone) |  |  | Physician (specialist) |
|  |  | Volunteers, | ALS, | Physician (specialist)*, |  |
|  |  | Firemen, | First responder program* | Physician* |  |
|  |  | Emergency medical technician, |  |  |  |
|  |  | Paramedic, |  |  |  |
|  |  | Nurse*, |  |  |  |
|  |  | Physician (on-scene), |  |  |  |
| Ireland | 2 | Physician (available by phone)* |  |  | Physician (specialist) |
|  |  | Volunteers, | ALS, | Physician (in some parts of the country), |  |
|  |  | Firemen, | First responder program | Paramedic (in some parts of the country) |  |
|  |  | Emergency medical technician, |  |  |  |
|  |  | Paramedic, |  |  |  |
|  |  | Physician (on-scene)*, |  |  |  |
| Italy | 2 | Physician (available by phone)* |  | Physician (specialist) | Physician (specialist) |
|  |  | Volunteers*, | ALS, |  |  |
|  |  | Nurse, | First responder program* |  |  |
|  |  | Physician (on-scene), |  |  |  |
| Kosovo | 1 | Physician (available by phone)* | ALS | Physician | Nurse |
|  |  | Nurse, |  |  |  |
| Luxembourg | 2 | Physician (on-scene) | ALS | Physician (specialist) | Nurse |
|  |  | Volunteers, |  |  |  |
|  |  | Firemen, |  |  |  |
|  |  | Emergency medical technician*, |  |  |  |
|  |  | Nurse, |  |  |  |
|  |  | Physician (on-scene), |  |  |  |
| Malta | 2 | Physician (available by phone)* |  | Physician (specialist) |  |
|  |  | Ambulance Emergency Responders, | ALS, |  | Physician (specialist)*, |
|  |  | Civil Protection Department, | First responder program* |  | Nurse |
|  |  | Firemen*, |  |  |  |
|  |  | Nurse, |  |  |  |
|  |  | Physician (on-scene), |  |  |  |
| Netherland | 6 | Physician (available by phone) |  | Physician (specialist) | Physician (specialist) |
|  |  | Volunteers, | ALS, |  |  |
|  |  | Firemen, | First responder program |  |  |
|  |  | Emergency medical technician*, |  |  |  |
|  |  | Paramedic, |  |  |  |
|  |  | Nurse, |  |  |  |
|  |  | Physician (on-scene), |  |  |  |
| Norway | 14 | Physician (available by phone) |  | Physician (specialist) | Physician (specialist) |
|  |  | Volunteers, | ALS, |  |  |
|  |  | Firemen, | First responder program |  |  |
|  |  | Emergency medical technician, |  |  |  |
|  |  | Paramedic, |  |  |  |
|  |  | Nurse, |  |  |  |
|  |  | Physician (on-scene), |  |  |  |
| Poland | 2 | Physician (available by phone) | ALS | Physician (specialist) |  |
|  |  | Paramedic, |  |  | Nurse*, |
|  |  | Nurse, |  |  | No personnel available by phone* |
| Portugal | 2 | Physician (on-scene) | ALS | Physician (specialist) | Physician (specialist) |
|  |  | Volunteers*, |  |  |  |
|  |  | Firemen, |  |  |  |
|  |  | Emergency medical technician, |  |  |  |
|  |  | Nurse, |  |  |  |
|  |  | Physician (on-scene), |  |  |  |
| Russia | 1 | Physician (available by phone) | ALS | Physician (specialist) | Physician (specialist) |
|  |  | Feldsher (mid-level EMS practitioner on-scene/by phone), |  |  |  |
|  |  | Nurse, |  |  |  |
|  |  | Physician (on-scene), |  |  |  |
| Serbia | 1 | Physician (available by phone) | ALS | Physician (specialist) | Emergency medical technician |
|  |  | Emergency medical technician |  |  |  |
| Slovenia | 4 | Physician (on-scene) |  |  | Physician (specialist) |
|  |  | Firemen | ALS, | Physician (specialist), |  |
|  |  | Emergency medical technician | First responder program* | Physician* |  |
|  |  | Paramedic* |  |  |  |
|  |  | Nurse |  |  |  |
|  |  | Physician (on-scene), |  |  |  |
| Spain | 2 | Physician (available by phone) |  | Physician (specialist) |  |
|  |  | Volunteers*, | ALS, |  | Physician (specialist)*, |
|  |  | Firemen*, | First responder program* |  | Physician* |
|  |  | Emergency medical technician, |  |  |  |
|  |  | Nurse*, |  |  |  |
|  |  | Physician (on-scene), |  |  |  |
| Sweden | 2 | Physician (available by phone) |  | Physician (specialist) | Physician (specialist) |
|  |  | Volunteers*, | ALS, |  |  |
|  |  | Firemen, | First responder program* |  |  |
|  |  | Policemen*, |  |  |  |
|  |  | Emergency medical technician, |  |  |  |
|  |  | Paramedic*, |  |  |  |
|  |  | Nurse, |  |  |  |
|  |  | Physician (on-scene), |  |  |  |
| Switzerland | 2 | Physician (available by phone) |  | Physician (specialist) | Physician (specialist) |
|  |  | Volunteers, | ALS, |  |  |
|  |  | Emergency medical technician, | First responder program |  |  |
|  |  | Paramedic, |  |  |  |
|  |  | Physician (on-scene), |  |  |  |
| United Kingdom | 4 | Physician (available by phone) |  | Physician (specialist) | Physician (specialist) |
|  |  | Volunteers, | ALS, |  |  |
|  |  | Firemen*, | First responder program |  |  |
|  |  | Emergency medical technician, |  |  |  |
|  |  | Paramedic, |  |  |  |
|  |  | Nurse, |  |  |  |
|  |  | Physician (on-scene), |  |  |  |
|  |  | Physician (available by phone) |  |  |  |
| North America | | | | | |
| Canada | 2 |  | ALS | Paramedic | Physician (specialist) |
|  |  | Firemen, |  |  |  |
|  |  | Emergency medical technician*, |  |  |  |
|  |  | Paramedic, |  |  |  |
|  |  | Nurse*, |  |  |  |
| Mexico | 1 | Physician (available by phone) | ALS | Paramedic | Physician (specialist) |
|  |  | Volunteers, |  |  |  |
|  |  | Firemen*, |  |  |  |
|  |  | Emergency medical technician, |  |  |  |
|  |  | Paramedic, |  |  |  |
| United States (Midwest) | 12 | Physician (available by phone) |  |  | Physician (specialist) |
|  |  | Volunteers, | ALS, | Physician (in some parts of the region), |  |
|  |  | Firemen, | First responder program (in some parts of the region) | Paramedic (in some parts of the region) |  |
|  |  | Emergency medical technician, |  |  |  |
|  |  | Paramedic, |  |  |  |
|  |  | Nurse, |  |  |  |
|  |  | Physician (on-scene) - in some parts of the region, |  |  |  |
| United States (Northeast) | 2 | Physician (available by phone) | ALS | Paramedic | Physician (specialist) |
|  |  | Volunteers*, |  |  |  |
|  |  | Firemen, |  |  |  |
|  |  | Emergency medical technician, |  |  |  |
|  |  | Paramedic, |  |  |  |
|  |  | Physician (on-scene) - in some parts of the region, |  |  |  |
| United States (South) | 15 | Physician (available by phone) |  |  | Physician (specialist) |
|  |  | Volunteers, | ALS, | Physician (in some parts of the region), |  |
|  |  | Firemen, | First responder program (in some parts of the region) | Paramedic (in some parts of the region) |  |
|  |  | Emergency medical technician, |  |  |  |
|  |  | Paramedic, |  |  |  |
|  |  | Nurse - in some parts of the region, |  |  |  |
|  |  | Physician (on-scene) - in some parts of the region, |  |  |  |
| United States (West) | 6 | Physician (available by phone) |  |  | Physician (specialist) |
|  |  | Volunteers, | ALS, | Physician (in some parts of the region), |  |
|  |  | Firemen, | First responder program* | Paramedic (in some parts of the region) |  |
|  |  | Emergency medical technician, |  |  |  |
|  |  | Paramedic, |  |  |  |
|  |  | Nurse*, |  |  |  |
|  |  | Physician (on-scene)*, |  |  |  |
|  |  | Physician (available by phone) |  |  |  |
| Oceania | | | | | |
| Australia | 4 |  |  |  | Physician (specialist) |
|  |  | Volunteers, | ALS, | Physician (specialist) – in some parts of the country |  |
|  |  | Emergency medical technician*, | First responder program | Paramedic |  |
|  |  | Paramedic, |  |  |  |
|  |  | Nurse, |  |  |  |
|  |  | Physician (on-scene) - in some parts of the country, |  |  |  |
| New Zealand | 2 | Physician (available by phone)* |  | Paramedic | Physician (specialist) |
|  |  | Volunteers, | ALS, |  |  |
|  |  | Firemen, | First responder program |  |  |
|  |  | Emergency medical technician, |  |  |  |
|  |  | Paramedic, |  |  |  |
|  |  | Physician (available by phone) |  |  |  |
| South America | | | | | |
| Brazil | 1 |  | ALS | Physician (specialist) | Physician |
|  |  | Nurse, |  |  |  |
|  |  | Physician (on-scene), |  |  |  |
| Chile | 1 | Physician (available by phone) | ALS | Physician (specialist) | Physician (specialist) |
|  |  | Firemen, |  |  |  |
|  |  | Paramedic, |  |  |  |
|  |  | Nurse, |  |  |  |
|  |  | Physician (on-scene), |  |  |  |
| Peru | 1 | Physician (available by phone) | ALS | Physician (specialist) | Physician (specialist) |
|  |  | Volunteers, |  |  |  |
|  |  | Firemen, |  |  |  |
|  |  | Emergency medical technician, |  |  |  |
|  |  | Paramedic, |  |  |  |
|  |  | Nurse, |  |  |  |
|  |  | Physician (on-scene), |  |  |  |

**Supplementary file 3 - Initiation of resuscitation (matrix)**

|  | **Initiation of resuscitation** | | |
| --- | --- | --- | --- |
|  | **All patients** | **All patients without obvious clinical signs of irreversible death** | **All patients without acceptable advanced directives** |
| **Africa** |  |  |  |
| Kenya |  | Initiation of resuscitation begins from the dispatch centre (if indicated ie no pulse, not breathing unless there are obvious signs of death such as decomposition, rigor mortis and decapitation) where medical instructions are given to the person on the ground to begin hands-only CPR. | It is also terminated when evidence of a DNR is presented. |
| Rwanda | in Rwanda we do not prioritize and even refrain during resuscitation in EMS system. |  |  |
| Sierra Leone |  | Prehospital healthcare professionals can refrain from resuscitation if obvious clinical signs of irreversible death are present. |  |
| South Africa |  | Prehospital resuscitation measures are initiated in all emergency situations where no clear loss of life (based on set criteria) has been confirmed. BLS providers always start CPR unless obviously dead. |  |
| Tunisia |  | Prehospital healthcare professionals can refrain from resuscitation if obvious clinical signs of irreversible death are present |  |
| Uganda |  | Prehospital healthcare professionals can refrain from resuscitation if obvious clinical signs of irreversible death are present | Prehospital healthcare professionals are legally obliged to adhere to a DNACPR |
| **Asia** |  |  |  |
| China |  | Unless the conditions fit the criteria of obvoius death, our EMS will perform BLS to all cardiac arrest cases, until they reach public hospital AEDs |  |
| India | Following the initial treatment, the patient is then transported to the emergency room of the nearest government hospital. After ascertaining the state of cardiac arrest, chest compression is started as per the standard protocol and arrangement is made to shift the patient to the nearby hospital |  |  |
| Japan |  | All cardiac arrests except apparent death (decapitation, trunk amputation, carbonization, brain prolapse, rigor mortis, and livor mortis) |  |
| Jordan |  | Prehospital healthcare professionals can refrain from resuscitation if obvious clinical signs of irreversible death are present |  |
| Malaysia |  | It is appropriate to withhold CPR intervention in the following situation: a. OHCA victim with signs of Clinical irreversible death | valid advanced directive indication resuscitation is not desired or also known as do not attempt resuscitation (DNAR) |
| Philippines |  | Withhold initiation if patient has signs of lividity and rigor mortis, obviously fatal injury. | If advanced directives are presented. |
| Qatar |  | when there is obvious signs of death like: > decomposition > rigor mortis > dependant lividity > decapitation > a valid DNR order > pre-term infant <20 weeks gestation > fetal maceration > evisceration of major organs > incineration | Prehospital healthcare professionals are legally obliged to adhere to a DNACPR |
| Saudi Arabia |  | Prehospital healthcare professionals can refrain from resuscitation if obvious clinical signs of irreversible death are present | Prehospital healthcare professionals are legally obliged to adhere to a DNACPR |
| Singapore |  | Only the patients with decomposed body, decapitation etc with no signs of life will not be resuscitated. | Prehospital healthcare professionals are legally obliged to adhere to a DNACPR |
| South Korea |  | When there are definitive clinical signs of death - Post-mortem rigidities (check 2 or more joints) - Rigor mortis Decapitation (cutting off the head) - Trunk amputation (cutting of the torso) - Brain parenchymal herniation due to trauma - Corruption (smelling, discoloration, deformation) | A medical indication that you do not want to perform CPR or do not attempt resuscitation (DNAR: Do Not Attempt Resuscitation) When a legal representative expresses his/her intention to refuse resuscitation Patients for whom a decision has been made to discontinue life-sustaining treatment in accordance with the Life-Sustaining Treatment Decision Act. |
| Sri Lanka | When they called for the scene, EMTs are starting the resuscitation and contact physician over the phone for further assistance. |  |  |
| Taiwan |  | If the patient’s head is not connected to the body or obvious rigor mortis, etc. | If the patient’s is in DNR status |
| Thailand |  | We start resuscitation in all case if the patients not met this criterion (Criteria for Not Starting CPR in All OHCA)  Signs of Irreversible Death: Resuscitation is not initiated if there are obvious clinical signs of irreversible death, such as rigor mortis, dependent lividity, decapitation, transection, or decomposition. | We start resuscitation in all case if the patients not met this criterion (Criteria for Not Starting CPR in All OHCA) Advance Directives: A valid, signed, and dated advance directive or a Do Not Attempt Resuscitation (DNAR) order that indicates the patient does not desire resuscitation must be respected. |
| Turkey |  | Prehospital healthcare professionals can refrain from resuscitation if obvious clinical signs of irreversible death are present |  |
| United Arab Emirates |  | All cases of non obvious death are resuscitated and transported |  |
| **Central America** |  |  |  |
| Costa Rica |  | If the staff considers that the patient has been in that state for a long time or that she shows signs of being dead for hours or days, they make medical contact by phone to request authorization not to perform CPR. | Prehospital healthcare professionals are legally obliged to adhere to a DNACPR |
| **Europa** |  |  |  |
| Austria |  | Only by prehospital physician ( or in the case of certain signs of death (e.g. decapitation, lividity or rigor mortis) also by EMT or paramedic that a CPR is not started |  |
| Belgium |  | If certain signs of death paramedics will not start resuscitation. First line ambulances with 2 EMTs (or with a nurse) would always start BLS except in case of obvious dead (rigor mortis, livedo, decapitation) or except when an advance directive is available. | First line ambulances with 2 EMTs (or with a nurse) would always start BLS except in case of a DNR order. |
| Bosnia and Herzegovina |  | Prehospital healthcare professionals can refrain from resuscitation if obvious clinical signs of irreversible death are present. |  |
| Croatia |  | EMS professionals initialize resuscitation in case of SCA if there are no certain signs of death. EMS team initiate resuscitation if there is no illness or injury not capable with life. |  |
| Czech Republic |  | Paramedics and nurses must start CPR when signs of death, advanced directive or terminal state are not present.  Initiation of resuscitation - whenever it is not present - injury incompatible with life, presence of certain signs of death. Development of post-mortem stains | Paramedics and nurses must start CPR when advanced directive or terminal state are not present.  Initiation of resuscitation - whenever it is not present - terminal stage of incurable disease..., a previously expressed wish (don´t resuscitation), |
| Denmark |  | EMTs / paramedics must initiate CPR unless the patient shows late signs of life extinct. Onsite determined or described (phone/radio) irreversible signs of death. | Confirmed written DNAR statement. |
| Finland |  | Resuscitaiton initiated in not clear secondary signs of death. | If there is no DNR, resuscitation is usually initiated. If unclear for DNR cotanct to HEMS. |
| France |  | Firefighters or emergency technician are obligated to start CPR unless there are obvious sings of death (decapitation, rigor mortis) | Prior written decision of the patient |
| Germany |  | Prehospital healthcare professionals can refrain from resuscitation if obvious clinical signs of irreversible death are present | If a living will is available or if the underlying disease is hopeless. DNR order. |
| Greece |  | Resuscitation is terminated pre-hospitaly incompatible with life injuries | Resuscitation is terminated pre-hospitaly for verified end stage illness , geriatric population. |
| Greenland |  | cardiac arrest, no definite signs of death (livores, rigor etc) |  |
| Hungary |  | Normal procedure is to initiate resuscitation if there are no obvious sings of death. | Prehospital healthcare professionals are legally obliged to adhere to a DNACPR |
| Iceland |  | Rigor/Livor mortis Decapitation or other obvious non-survivable injuries. | Prehospital healthcare professionals are legally obliged to adhere to a DNACPR |
| Ireland |  | Resuscitation is started in the presence of indicators of cardiac arrest but in the absence of Indicators of death | Resuscitation not commenced if DNACPR documentation is available to the attending crew |
| Italy |  | The practice is based on International CPR life support guidelines that healthcare workers apply in every case of cardiac arrest without clear signs of death. In this case the practice is based on international guidelines and presence of evident signs of death. | In this case the practice is based on international guidelines and presence of terminal illness. |
| Kosovo | There is no prehospital refraining! |  |  |
| Luxembourg |  | All attending EMS members are advised to start CPR (unless obvious signs of death, like rigor, lesions incompatible with life,...) until the EMS doctor arrives on scene and decides how to further proceed. |  |
| Malta |  | Emergency nurses initiate resuscitation on everyone unless obvious sigs of futility |  |
| Netherlands |  | The resuscitation is initated, unless there are clear reasons not to do so (clearly dead on arrival, etc).It will not start if:- Symptoms of biological death | The resuscitation is initated, unless there are clear reasons not to do so (Do not resuscitate, etc).It will not start if:- Advance directive or pre-registered do not resuscitate statement.It will also be discontinued if it becomes known that a patient has an advance directive or do not resuscitate statement. |
| Norway |  | You always start unless there is obvious signs that the patient is dead (rigor mortis, obvious cold without possibility of accidential hypothermia and long time since last observation, trauma not compatible with life) or valid DNR order. Practice is to start of continue resuscitation unless there are obvious signs of death (i.e. rigor mortis). No obvius signe of dead startes CPR. Resuscitation initiated unless: - Signs of irreversible death - Injuries incompatible with life | No known DNR order. You always start unless there is a valid DNR order. |
| Poland |  | Prehospital healthcare professionals can refrain from resuscitation if obvious clinical signs of irreversible death are present |  |
| Portugal |  | The medical doctor on-site may decide not to star CPR. Situations like the head separated form the body, decomposition, etc. |  |
| Russia |  | The legislation states generally that CPR should not be commenced in cases of "signs of biological death" and "consequences of acute injury incompatible with life". |  |
| Serbia |  | Prehospital healthcare professionals can refrain from resuscitation if obvious clinical signs of irreversible death are present |  |
| Slovenia |  | If cardiac arrest is recognized by the EMS dispatcher during an emergency phone-call (without obvious signs of death) the witnesses are encouraged to start CPR. | Prehospital healthcare professionals are legally obliged to adhere to a DNACPR |
| Spain |  | Prehospital healthcare professionals can refrain from resuscitation if obvious clinical signs of irreversible death are present | When you have terminal illness or orders of non-resuscitation. |
| Sweden |  | Ground ambulance are according to their SOPs allowed to refrain from starting cpr according to a few criteria (for example rigor mortis, head away from body). | Some patients with DNR at nursing homes can be terminated according to specific guidelines. |
| Switzerland |  | Prehospital healthcare professionals can refrain from resuscitation if obvious clinical signs of irreversible death are present | Prehospital healthcare professionals are legally obliged to adhere to a DNACPR |
| United Kingdom |  | In the presence of features incompatible with life e.g., decapitation, hemicorporectomy, livor mortis. | If arrest is viable and no DNACPR/DNAR, resus must be attempted |
| **North America** |  |  |  |
| Canada |  | Commence CPR unless any of the following 1. obviously dead. | 2. the subject of a medical certificate of death, presented to the paramedic crew, in the form that is prescribed by the Vital Statistics Act (Ontario) and that appears on its face to be completed and signed in accordance with that Act; 3. without vital signs and the subject of an MOH Do Not Resuscitate (DNR) confirmation Form; 4. without vital signs and the subject of a Termination of Resuscitation (TOR) Order given by a Base Hospital Physician; or 5. without vital signs and the subject of a Withhold Resuscitation Order given by a Base Hospital Physician. |
| Mexico |  | Prehospital healthcare professionals can refrain from resuscitation if obvious clinical signs of irreversible death are present | In a few cases the patients' family members can choose not to begin resuscitation but show a legal document regarding the patient's will not to be resuscitated. |
| United states (California) |  | 1. Obvious Death: decapitation, incineration, decomposition, rigor mortis, obvious destruction of brain, heart or lungs. | 2. Physician Orders for Life Saving -Sustaining Treatment, or Do Not Resuscitate Order |
| United states (Colorado) |  | Death in the Field Indicationsa. An obvious death in the field is a standing order if the below criteria is met. b. Determination of death in the field (without initiation of resuscitation) should include the following instances;• Patient is unresponsive, pulseless, apneic, ANDi. Decomposition, orii. Rigor mortis or dependent lividity with warm air temperature | Any advanced directive orPhysician orders as specified on the Colorado Medical Orders for Scope of Treatment (MOST) form: "No CPR. Do Not Resuscitate/DNR/Allow Natural Death" |
| United states (Florida) |  | Cardiopulmonary resuscitation will be initiated on all patients who have suffered a  cardiopulmonary arrest, except for those with obvious signs of irreversible biological death. | Cardiopulmonary resuscitation will be initiated on all patients who have suffered a cardiopulmonary arrest, except for those with a valid Do Not Resuscitate Order (DNRO) |
| United states (Illinois) |  | Resuscitation should be withheld in the following circumstances: 1. Medical signs of long term death including: a. Rigor Mortis: Stiffening of the body muscles due to chemical changes in muscle  fibers, plus asystole on cardiac monitor in multiple leads. b. Widespread Lividity: Skin discoloration in dependent body parts, plus asystole on cardiac monitor in multiple leads. c. Decomposition or Putrefaction: The skin is bloated or ruptured, with or without  soft tissue sloughed off, plus asystole on cardiac monitor in multiple leads. 2. Traumatic injuries obviously incompatible with life including: a. Decapitation: The complete severing of the head from the patient’s body. b. Transection of the Torso: The body is completely cut across below the shoulders and above the hips through all major organs and vessels. The spinal column may or may not be severed. c. Incineration: 90% of the body surface area with full thickness burns as exhibited  by ash rather than clothing and complete absence of body hair with charred skin. | Unless a patient meets obvious death criteria or **has a valid DNR/POLST**, CPR should be initiated. |
| United states (Intermountain) |  | Prehospital healthcare professionals can refrain from resuscitation if obvious clinical signs of irreversible death are present | Termination of resuscitation can be initiated by documentation of a POLST or advanced directive in certain saturations. |
| United states (Massachusetts) |  | I. Exceptions to Initiation of ResuscitationOther than in overriding circumstances such as a large mass-casualty incident or a hazardous scene,the following are the only exceptions to initiating and maintaining resuscitative measures in the field:3. Trauma inconsistent with survivala. Decapitation: severing of the vital structures of the head from the remainder of thepatient’s bodyb. Transection of the torso: body is completely cut across below the shoulders andabove the hipsc. Evident complete destruction of brain or heartd. Incineration of the bodye. Cardiac arrest (i.e. pulselessness) documented at first EMS evaluation when suchcondition is the result of significant blunt or penetrating trauma and the arrest isobviously and unequivocally due to such trauma, EXCEPT in the specific case ofarrest due to penetrating chest trauma and short transport time to definitive care (inwhich circumstance, resuscitate and transport).4. Body condition clearly indicating biological death.a. Complete decomposition or putrefaction: the skin surface (not only in isolatedareas) is bloated or ruptured, with sloughing of soft tissue, and the odor of decayingflesh.b. Dependent lividity and/or rigor: when the patient’s body is appropriately examined,there is a clear demarcation of pooled blood within the body, and/or major joints(jaw, shoulders, elbows, hips, or knees) are immovable. | 1. Current, valid DNR, verified per this protocol.2. Health care agent who is named in a health care proxy document for the patientrequests no resuscitative efforts on behalf of the patient, but only if 1) he or she is onscene and 2) he or she has his/her health care proxy document in hand to show EMS. Ifthere is any doubt about the health care agent’s authority, and none of the otherexceptions to initiation of resuscitation are present, EMS is to resuscitate the patient. |
| United states (Michigan) |  | Initiate or continue CPR for patient found to be in cardiac arrest UNLESS one or more of the following conditions exists: A. Decomposition B. Rigor mortis (Caution: do not confuse with stiffness due to cold environment) C. Dependent lividity D. Decapitation E. Incinerated or frozen body | Patient has a valid "Do Not Resuscitate" identification bracelet or order. |
| United states (Midwest) |  | No obvious signs of death, no signs of unsurvivable injury | No DNR |
| United states (Mississippi) |  | Prehospital healthcare professionals can refrain from resuscitation if obvious clinical signs of irreversible death are present | Prehospital healthcare professionals are legally obliged to adhere to a DNACPR |
| United states (Missouri) |  | Injuries incompatible with life. Lividity. Rigor Mortis. Tissue decomposition. Mass Casualty Incident where resources are not available to attempt or  continue prolonged resuscitation. | DNR or Living Will |
| United states (Nebraska) |  | Prehospital healthcare professionals can refrain from resuscitation if obvious clinical signs of irreversible death are present | Prehospital healthcare professionals are legally obliged to adhere to a DNACPR |
| United states (New England) |  | Prehospital healthcare professionals can refrain from resuscitation if obvious clinical signs of irreversible death are present | Prehospital healthcare professionals are legally obliged to adhere to a DNACPR |
| United states (New Jersey) |  | BLS may elect not to resuscitate if injuries incompatible with life; or rigor, lividity or decomposition present. | BLS may elect not to resuscitate if there is a valid out of hospital DNR order. |
| United states (Ohio) |  | Basic and/or Advanced cardiac life support must be started on all patients who are found apneic and pulseless, UNLESS: B. There is an injury that is incompatible with life, (such as decapitation, hemicorporectomy, or burned beyond recognition). Isolated penetrating trauma should rarely be considered incompatible with life OR C. The victim shows signs of rigor mortis (in a warm environment), dependent lividity, or decomposition. | Basic and/or Advanced cardiac life support must be started on all patients who are found apneic and pulseless, UNLESS: A. A valid Do Not Resuscitate order is presented as defined in the Do Not Resuscitate Protocol |
| United states (Oklahoma) |  | Unless obvious signs of terminal death, resuscitation is initiated when a patient, regardless of age, is found to be apneic and pulseless. | Do Not Resuscitate orders and/or Advanced Directives in other formats are honored when presented to on-scene EMS professionals. |
| United states (Oregon) |  | Resuscitation efforts may be withheld if: B. The patient is pulseless and apneic in a mass casualty incident or multiple patient scene where the resources of the system are required for the stabilization of living patients. C. The patient is decapitated. D. The patient has rigor mortis in a warm environment. E. The patient is in the stages of decomposition. F. The patient has skin discoloration in dependent body parts (dependent lividity). | Resuscitation efforts may be withheld if: A. The patient has a valid, signed "DNR” order. POLST registry # 888-476-5787 |
| United states (Tennessee) |  | If no criteria of obvious death, resus is initiated. | Prehospital healthcare professionals are legally obliged to adhere to a DNACPR |
| United states (Texas) |  | Determination of obvious signs of death first, then determination of rhythm (for traumatic). EMS Providers are NOT required to initiate resuscitation measures if the patient is apneic and pulseless, AND a cardiac rhythm strip shows asystole AND one of the following: Rigor mortis Dependent lividity Decomposition Valid DNR Decapitation Incineration We begin resuscitation in the presence of cardiac arrest WITHOUT: Signs of obvious death (mortal injuries, lividity, rigor, cold temperature) | EMS Providers are NOT required to initiate resuscitation measures if the patient is apneic and pulseless, AND a cardiac rhythm strip shows asystole AND a Valid DNR. We begin resuscitation in the presence of cardiac arrest WITHOUT: Do Not Resuscitate Orders |
| United states (Utah-san Juan) |  | Automatic criteria for the initiation of resuscitation based on time down, physiologic parameters (rigor, lividity, asystole in trauma) | Prehospital healthcare professionals are legally obliged to adhere to a DNACPR |
| United states (Virginia) |  | Unless there are **obvious signs of death** or a "do not resuscitate" order immediately available, protocol is to initiate CPR. Resuscitation is to be started unless there are **obvious signs of death or injury incompatible with life (rigor, decapacitation, injury with brain matter showing**) or presence of a prehospital do not resuscitate order. | Unless there are obvious signs of death or a "**do not resuscitate" order** immediately available, protocol is to initiate CPR. Resuscitation is to be started unless there are obvious signs of death or injury incompatible with life (rigor, decapacitation, injury with brain matter showing) or **presence of a prehospital do not resuscitate order**. |
| United states (Washington) |  | No lividity or decompostion or injury not compatible with life. INITIATION AND TERMINATION OF RESUSCITATION1. If any of the following conditions are present, do not initiate CPR:Cardiac arrest in the field due to blunt force trauma.Decapitation, total incineration, or obviously nonsurvivable injuries (i.e., extruded brain material).Decomposition, patient is cold in a warm environment, dependent lividity, or rigor mortis.Underwater submersion for 2 or more hours (consider extending 2-hour time if water temperature near freezing).Mass casualty situation where triage principles preclude CPR from being initiated on every victim.Discuss with medical control whenever possible after 15 minutes of ALS resuscitation for advice on treatment, termination, or transport. | Pt does not have DNR or POLST. Patient has a terminal illness and next of kin requests not to perform CPR. Valid advanced directive indicating no resuscitation. |
| United states (Wisconsin) |  | Prehospital healthcare professionals can refrain from resuscitation if obvious clinical signs of irreversible death are present | Prehospital healthcare professionals are legally obliged to adhere to a DNACPR |
| **Oceania** |  |  |  |
| Australia |  | Prehospital healthcare professionals can refrain from resuscitation if obvious clinical signs of irreversible death are present | Prehospital healthcare professionals are legally obliged to adhere to a DNACPR |
| New Zealand |  | Resuscitation should start unless there is a clear reason not to. Clear reasons for not starting resuscitation include: Signs of rigor mortis or post-mortem lividity. | Resuscitation should start unless there is a clear reason not to. Clear reasons for not starting resuscitation include: A clear advance directive not to receive resuscitation for cardiac arrest. |
| **South America** |  |  |  |
| Brazil |  | The determination to stop efforts is difficult, and some situations can help in deciding not to initiate CPR or to determine the end of CPR efforts: • When there are signs of obvious death or an irreversible clinical condition | Metastatic cancer or other terminal pathology (with no possibility of treatment, described in a medical report or informed by a responsible family member). • Cardiac arrest in terminal stage patients with severe and incurable illnesses with family consensus not to perform resuscitation. |
| Chile |  | Once a patient looks without life signs and no rigor mortis. |  |
| Peru | No DNACPR or similar exist in your EMS system and prehospital healthcare professionals can not legally refrain from resuscitation if obvious clinical signs of irreversible death are present |  |  |

**Supplementary file 4 – Initiation of resuscitation table**

| **Country**  **(Region)** | **The instructions that determine the practice regarding**  **initiation of resuscitation** | **The practice regarding**  **initiation of resuscitation** | **The variations in the practice regarding initiation of resuscitation** |
| --- | --- | --- | --- |
| **Africa** |  |  |  |
| Kenya | Standardized protocols, Rules, Guidelines, Medical directives | All patients without obvious signs of irreversible death. All patients without confirmed advanced directives. | EMS-system organization, Response time, Differences in work-flow, Differences in treatment facilities, Infrastructure |
| Rwanda | Standardized protocols,  Rules,  Guidelines,  Medical directives,  Online medical control | All patients | EMS-system organization,  Geographical allocations |
| Sierra Leone | Standardized protocols,  Guidelines,  Decision-making without the use of  guidelines or protocols | All patients without obvious signs of irreversible death. | Response time,  Differences in work-flow,  Differences in treatment facilities |
| South Africa | Standardized protocols, Rules, Guidelines, Medical directives | All patients without obvious signs of irreversible death. | EMS-system organization, Response time, Differences in work-flow, Differences in treatment facilities, Level of training, Topographical challenges, Transport distances |
| Tunisia | Standardized protocols,  Rules,  Guidelines,  Medical directives,  Online medical control | All patients without obvious signs of irreversible death. | EMS-system organization,  Response time,  Differences in treatment facilities |
| Uganda | Standardized protocols, Decision-making without the use of guidelines or protocols,  On-site medical control | All patients without obvious signs of irreversible death.  All patients without confirmed advanced directives. | Response time,  Differences in treatment facilities,  Differences in first responders,  Rural vs. Urban |
| **Asia** |  |  |  |
| China | Standardized protocols, Rules, Guidelines | All patients without obvious signs of irreversible death. | EMS-system organization, Response time, Differences in work-flow, Differences in treatment facilities |
| India | Standardized protocols,  Decision-making without the use of guidelines or protocols | All patients | EMS-system organization, Response time, Differences in work-flow,  Rural vs. urban |
| Japan | Standardized protocols, Rules, Guidelines, Medical directives, On-site medical control | All patients without obvious signs of irreversible death. | No variation described |
| Jordan | Rules, Guidelines, Decision-making without the use of guidelines or protocols (the medical direction is still limited) | All patients without obvious signs of irreversible death. | No variation described |
| Malaysia | Standardized protocols, Guidelines,  Medical directives,  Online medical control,  On-site medical control | All patients without obvious signs of irreversible death.  All patients without confirmed advanced directives. | No variation described |
| Philippines | Standardized protocols, Guidelines, Medical directives, Online medical control | All patients without obvious signs of irreversible death. All patients without confirmed advanced directives. | EMS-system organization, Response time, Differences in work-flow, Differences in treatment facilities |
| Qatar | Standardized protocols,  Guidelines,  Medical directives | All patients without obvious signs of irreversible death.  All patients without confirmed advanced directives. | No variation described |
| Saudi Arabia | Standardized protocols, Rules, Guidelines, Medical directives, Online medical control, Decision-making without the use of guidelines or protocols, On-site medical control | All patients without obvious signs of irreversible death.  All patients without confirmed advanced directives. | Differences in work-flow |
| Singapore | Standardized protocols, Online medical control | All patients without obvious signs of irreversible death. All patients without confirmed advanced directives. | No variation described |
| South Korea | Standardized protocols, Guidelines | All patients without obvious signs of irreversible death.  All patients without confirmed advanced directives. | No variation described |
| Sri Lanka | Standardized protocols, Online medical control | All patients | No variation described |
| Taiwan | Standardized protocols, Rules, Guidelines | All patients without obvious signs of irreversible death. All patients without confirmed advanced directives. | EMS-system organization, Differences in treatment facilities |
| Thailand | Standardized protocols, Guidelines, Online medical control, On-site medical control | All patients without obvious signs of irreversible death. All patients without confirmed advanced directives. | EMS-system organization, Response time, Differences in work-flow, Differences in treatment facilities, Availability of advanced medical resources, Rural versus urban areas |
| Turkey | Standardized protocols,  Guidelines | All patients without obvious signs of irreversible death. | Response time,  Differences in work-flow |
| United Arab Emirates | Rules,  Decision-making without the use of guidelines or protocols,  Law | All patients without obvious signs of irreversible death. | No variation described |
| **Central America** |  |  |  |
| Costa Rica | Decision-making without the use of guidelines or protocols | All patients without obvious signs of irreversible death.  All patients without confirmed advanced directives. | No variation described |
| **Europa** |  |  |  |
| Austria | Standardized protocols, Rules, guidelines,  Medical directives | All patients without obvious signs of irreversible death. | EMS-system organization,  Response time, Differences in work-flow |
| Belgium | Rules,  guidelines | All patients without obvious signs of irreversible death.  All patients without confirmed advanced directives. | No variation described |
| Bosnia and Herzegovina | Standardized protocols | All patients without obvious signs of irreversible death. | EMS-system organization |
| Croatia | Guidelines | All patients without obvious signs of irreversible death. | No variation described |
| Czech Republic | Standardized protocols, Rules, Guidelines, Medical directives, Decision-making without the use of guidelines or protocols, On-site medical control | All patients without obvious signs of irreversible death. All patients without confirmed advanced directives. | No variation described |
| Denmark | Standardized protocols,  Rules,  Guidelines,  Medical directives,  Decision-making without the use of guidelines or protocols,  On-site medical control | All patients without obvious signs of irreversible death.  All patients without confirmed advanced directives. | No variation described |
| Finland | Standardized protocols, Rules, Guidelines, Medical directives, Online medical control, On-site medical control | All patients without obvious signs of irreversible death. All patients without confirmed advanced directives. | EMS-system organization,  Response time. |
| France | Standardized protocols  Decision-making without the use of guidelines or protocols | All patients without obvious signs of irreversible death.  All patients without confirmed advanced directives. | No variation described |
| Germany | Standardized protocols, Guidelines, Medical directives, On-site medical control | All patients without obvious signs of irreversible death. All patients without confirmed advanced directives. | EMS-system organization (first responders) |
| Greece | Rules, Guidelines, On-site medical control | All patients without obvious signs of irreversible death. | EMS-system organization, Response time, Differences in treatment facilities, Limited accessibility to training resources, Rural versus urban areas |
| Greenland | Rules, Guidelines | All patients without obvious signs of irreversible death. | EMS-system organization, Response time, Differences in treatment facilities, Rural versus urban areas |
| Hungary | Standardized protocols, Guidelines, Decision-making without the use of guidelines or protocols | All patients without obvious signs of irreversible death. All patients without confirmed advanced directives. | No variation described |
| Iceland | Standardized protocols, Guidelines, Medical directives, Online medical control | All patients without obvious signs of irreversible death. All patients without confirmed advanced directives. | No variation described |
| Ireland | Standardized protocols,  Guidelines,  Medical directives,  Online medical control | All patients without obvious signs of irreversible death.  All patients without confirmed advanced directives. | No variation described |
| Italy | Standardized protocols, Guidelines, Medical directives, On-site medical control | All patients without obvious signs of irreversible death. All patients without confirmed advanced directives. | No variation described |
| Kosovo | Standardized protocols,  Guidelines,  Medical directives | All patients | No variation described |
| Luxembourg | Standardized protocols, Guidelines | All patients without obvious signs of irreversible death. | No variation described |
| Malta | Standardized protocols,  guidelines,  On-site medical control | All patients without obvious signs of irreversible death. | No variation described |
| Nederland | Standardized protocols, Rules, Guidelines, Medical directives, Decision-making without the use of guidelines or protocols, On-site medical control | All patients without obvious signs of irreversible death. All patients without confirmed advanced directives. | No variation described |
| Norway | Standardized protocols, Rules, Guidelines, Medical directives, Online medical control, Decision-making without the use of guidelines or protocols, On-site medical control | All patients without obvious signs of irreversible death. All patients without confirmed advanced directives. | EMS-system organization, Differences in workflow, Local interpretations. |
| Poland | Standardized protocols, Guidelines | All patients without obvious signs of irreversible death. | No variation described |
| Portugal | Standardized protocols, Medical directives,Online medical control | All patients without obvious signs of irreversible death.  All patients without confirmed advanced directives. | No variation described |
| Russia | Standardized protocols | All patients without obvious signs of irreversible death. | No variation described |
| Serbia | Guidelines, Decision-making without the use of guidelines or protocols | All patients without obvious signs of irreversible death. | EMS-system organization |
| Slovenia | Guidelines, Decision-making without the use of guidelines or protocols | All patients without obvious signs of irreversible death. | EMS-system organization, Availability and distribution of EMS |
| Spain | Standardized protocols, Guidelines, Decision-making without the use of guidelines or protocols, On-site medical control | All patients without obvious signs of irreversible death. All patients without confirmed advanced directives. | EMS-system organization, Differences in workflow |
| Sweden | Standardized protocols, Guidelines, Online medical control, On-site medical control | All patients without obvious signs of irreversible death. All patients without confirmed advanced directives. | No variation described |
| Switzerland | Standardized protocols, Guidelines, Medical directives, Decision-making without the use of guidelines or protocols, On-site medical control | All patients without obvious signs of irreversible death. All patients without confirmed advanced directives. | EMS-system organization, Response time. |
| United Kingdom | Standardized protocols, Rules, Guidelines, Medical directives, Decision-making without the use of guidelines or protocols, On-site medical control | All patients without obvious signs of irreversible death. All patients without confirmed advanced directives. | No variation described |
| **North America** |  |  |  |
| Canada | Standardized protocols, Medical directives, Online medical control | All patients without obvious signs of irreversible death. All patients without confirmed advanced directives. | No variation described |
| Mexico | Guidelines,  Medical directives,  Online medical control | All patients without obvious signs of irreversible death.  All patients without confirmed advanced directives. | Response time,  Differences in treatment facilities, Urban vs Rural areas, Levels of training and first response |
| United States - Midwest | Standardized protocols, Guidelines, Medical directives, Online medical control | All patients without obvious signs of irreversible death. All patients without confirmed advanced directives. | EMS-system organization, Response time, Differences in treatment facilities |
| United States - Northeast | Standardized protocols, Online medical control | All patients without obvious signs of irreversible death. All patients without confirmed advanced directives. | No variation described |
| United States - South | Standardized protocols, Guidelines, Medical directives, Online medical control | All patients without obvious signs of irreversible death. All patients without confirmed advanced directives. | EMS-system organization,  Response time |
| United States - West | Standardized protocols, Guidelines, Online medical control, On-site medical control | All patients without obvious signs of irreversible death. All patients without confirmed advanced directives. | No variation described |
| **Oceania** |  |  |  |
| Australia | Standardized protocols, Rules, Guidelines, Medical directives | All patients without obvious signs of irreversible death. All patients without confirmed advanced directives. | No variation described |
| New Zealand | Guidelines, Medical directives, Decision-making without the use of guidelines or protocols | All patients without obvious signs of irreversible death. All patients without confirmed advanced directives. | No variation described |
| **South America** |  |  |  |
| Brazil | Standardized protocols | All patients without obvious signs of irreversible death.  All patients without confirmed advanced directives. | No variation described |
| Chile | Standardized protocols,  guidelines | All patients without obvious signs of irreversible death.  All patients without confirmed advanced directives. | Don't know |
| Peru | Medical directives | All patients | Don't know |

**Supplementary file 5 – Termination of resuscitation (matrix)**

|  | **Termination of resuscitation** | | |
| --- | --- | --- | --- |
|  | **No termination of resuscitation at scene** | **Specific criteria/guidelines (e.g. time specific, guideline, advanced directives)** | **Discretion of the provider** |
| **Africa** |  |  |  |
| Kenya |  | You terminate resuscitation after 30 minutes of no success to get pulse despite cpr. Termination is considered usually after 20 to 30 minutes of active resuscitation attempts with no ROSC or after 5 cycles of CPR or when the patient achieves ROSC. Sometimes it is called when the medics are exhausted and there is not enough manpower to continue. |  |
| Rwanda | No termination of resuscitation in the prehospital setting |  |  |
| Sierra Leone |  |  | We normally stop resuscitation after about an hour. Still, patient is not responsive, and also, when central pulses are not felt, pupils dilated and not reactive to light, etc, tend to stop. |
| South Africa |  | Termination of resuscitation measures occur once ALS resuscitation measures for a reasonable period of time has not resulted in any signs of life (set criteria) | Resuscitation measures may be terminated if an assessment if made to determine neurological function. This may be performed after reasobale period (approx 20 min) ALS measures have been performed. There are also instances (decapitation, mortal disfigurement) were resuscitation measures may be deferred, in addition to palliation Paramedic can decide to terminate once they decide the process is futile. Guidelines recommend asystole with no reversible causes |
| Tunisia |  |  | it depend of the patient ( comorbidities , age....) - ERC guidelines.  We don't have any specific resuscitation guideline of the termination. We generally use the ERC guidelines. |
| Uganda |  |  | Termination may be done if resuscitation is done for 30 minutes without return of pulse activity. Due to limitation in resources, there may be termination as the next steps either during or post resuscitation cannot be undertaken. Family decisions also play a big role as resuscitation is not well acknowledged and sometimes may be mistaken as an act killing the patient. - Failure to connect to hospital with services. Family decision influenced by cultural norms |
| **Asia** |  |  |  |
| China | Unless the conditions fit the criteria of obvoius death, our EMS will perform BLS to all cardiac arrest cases, until they reach public hospital AEDs (ground transport time from scene to AED around 30min or less), where the doctors will decide whether continuing ALS or certify death. There is currently no widely accepted TOR rule in prehospital or even in the AED settings in Hong Kong, Once resuscitation has been initiated in the prehospital setting, it will be continued until reaching hospital AED. |  |  |
| India |  |  | There is no documentation uniformly accepted regarding termination of resuscitation. The rescuer decision is usually based on: Rescuer exhaustion The patient was already cold when the CPR was started The scene becomes unsafe Has done CPR for a sufficient period of time without success |
| Japan | We don't terminate resuscitation in the prehospital setting. |  |  |
| Jordan | Many Paramedics do not terminate resuscitation till they deliver patients to the hospital. However, the effectiveness of the resuscitation for many patients is questionable. EMS providers have to start resuscitation to all cardiac arrested patients. They need to transport the patient to the nearset hospital where termination of resuscitation can be declared by the ER physician. |  |  |
| Malaysia |  | Termination of resuscitation criteria in adult OHCA: require all this condition to be present: a. no transport criteria are present. b. no bystander CPR performed upon arrival of PHCS responder. c. No Prehospital ROSC after three cycles of CPR (6 to 8 minutes of CPR) d. Persistent asystole for three round of Rhythm Analysis. Execution of TOR in adult OHCA: a. Present of all TOR criteria in adult OHCA and absence of any exclusion criteria b. Discussion with online Medical Control regarding decision of TOR c. Obtaining cosent from immediate family members or next of kin regarding implementation of TOR. |  |
| Philippines | Once resuscitation is started, termination is not done in the prehospital setting. Not all local government units/regions have a structured EMS system and not all even have physicians. The laws of the land states that only physicians can declare death and, thus, also can terminate resuscitation efforts. For declaration of death in these situations, the patient need to be brought to the hospital. |  |  |
| Qatar |  | 1. When the patient is not breathing and no pulse. 2. Asystole for more than 20 minutes. 3. Broad PEA 9agonal rhythm) with < 40 bpm. 4. reversible causes have been managed. 5. the patient's family / significant other give verbal consent to stop CPR (if present on scene). > Following certain conditions as stated in the existing CPG - 20 minutes of: continuous Asytole despite performing ACLS CPR or broad PEA (agonal rhythm < 40 bpm) - reversible causes have been treated - cardiac arrest not from drug overdose and/or hypothermia - verbal consent from the patient’s family or significant others - no breathing - no palpable pulses. |  |
| Saudi Arabia | Usually no termination at scene. Morally we don’t unless it was confirmed dead on arrival |  |  |
| Singapore |  | Patients need to fulfil certain criteria eg not witnessed arrest, no bystander CPR, non shockable rhythm, no ROSC after 6 cycles. All OHCA cases that meet a set of criteria (as assessed on site by paramedic) will be considered for TOR. TOR requires the OHCA to be (1) not witnessed by EMS, (2) no shockable rhythm (3) no ROSC after minimum 6 CPR cycles. |  |
| South Korea |  |  | We are not accepting any TOR. Instead of the protocol TOR, on-duty medical director can decide termination of resuscitation after hearing the conditions from EMS providers. All resuscitations by EMS providers are directed by medical directors at the same time through telecommunication system. |
| Sri Lanka |  |  | We follow the ALS ERC guideline.  Discuss with the Emergency physician over the phone or after reaching the nearest healthcare service/ provider. They decide the termination. |
| Taiwan | We don't terminate resuscitation in the prehospital setting. |  |  |
| Thailand | In our EMS system, once resuscitation is initiated, we generally continue efforts until the patient can be transferred to a hospital. Decisions regarding the continuation or termination of resuscitation efforts are then typically made in the hospital's emergency room. In case of continuing resuscitation, most EMS providers will continue the CPR during transport to the nearest hospital. The termination of resuscitation will depend on the decision of attending physician at ED. |  |  |
| Turkey |  |  | We use ERC CPR guideline If it is a team with medical doctor, the doctor decide to terminate the CPR If the team do not have MD, paramedic team. Paramedics cannot terminate the CPR because in Türkiye paramedics can not decide if the person is death or not. |
| United Arab Emirates | No termination of resuscitation in the prehospital setting |  |  |
| **Central America** |  |  |  |
| Costa Rica |  |  | The personnel who are performing basic or advanced CPR, due to time or clinical condition, without a standard or guide, call the contact doctor by phone and according to the patient's data, the doctor decides if they should perform any more maneuvers or interventions |
| **Europa** |  |  |  |
| Austria |  |  | Usually, a prehospital physician (specialist) is available to attend the CPR and can terminate it anytime based on their own decisions/beliefs. In the case of EMTs alone on the scene, they would only consider a termination in case of an extended time of running CPR and exhaustion if no physician can attend the scene. |
| Belgium |  |  | Team decision.  If resuscitation efforts prove futile, (asystolie, > 20 minutes,...) decision to terminate is made in team. Prehospital emergency doctors will follow ERC guidelines on termination of resuscitation. |
| Bosnia and Herzegovina |  |  |  |
| Croatia |  |  | In Croatia there are physicians on the scene (some of them EM specialist) and they deicide about termination of resuscitation based on ERC guidelines and education (National EMS courses, ERC courses). Terminal cancer patients, injuries incapable with life, patients with multiple comorbidity who are also paliative patients, certain signs of death (livor mortis, rigor mortis) and decision is made by physician on scene. |
| Czech Republic |  |  | Physician on scene can determine other factors when considering initiation of CPR (patient history, quality of life, ethical considerations). Physician that is called on scene can terminate CPR based on clinical decision with the support of ERC GL and the rule of exclusion of 4H&4T, 20 mins of CPR and/or signs of death. The principles of Clinical Ethics (e.g. nonmaleficence is also applied). Consideration of the futility of prolonged resuscitation. |
| Denmark |  |  | Thus, in daily clinical practize, the decision to refrain from initiation may also be taken based on decision-making without this knowlegde. There are no standardised protocols or rules. Physicians can (and do) initiate or abstain from at their own discretion. Futility. Decision made by on-sight medical doctor due to the information available of medicla history and duration of treatment without ROSC. Resuscitation is generally terminated based on on-site decision making comprising information on age, medical history, functioning level, next-of kins information on the patients wishes, no-flow time, low-flow time, signs of life during resuscitation, rhythm, etCO2 levels, and ultrasound. Standardized termination rules or protocols do not exist. |
| Finland |  |  | Resuscitation can be terminated earlier, if the patients quality of life is poor and the resuscitation effort is hopeless, or if it is clear, that the patient will not be admitted to intensive care after ROSC. Time limits for CPR attempts are defined but physicians can deviate from these for good reason. |
| France |  |  | Medical decision of the physician on site without using a protocol. ERC guidelines are pretty vague about termination of resuscitation but they are the guidelines used. |
| Germany |  |  | The decision to terminate resuscitation is made individually by the emergency physician in charge at the scene. The patient's age, previous illnesses, a possible no flow time, the quality of the lay resuscitation and the first heart rhythm as well as the previous resuscitation process play a role. Different factors are taken into account: duration of CPR; witness status, initial and current rhythm, etCO2. There is no protocol or score for termination of resuscitation for our EMS system. age, past medical histery, whole circumstances on scene. |
| Greece |  | Only the doctor has right to terminate resuscitation - pre-hospitaly mainly/only for verified end stage illness , geriatric population and incompatible with life injuries usually after a minimum time of resuscitation efforts . No legally allowed DNACPR and many legislative unclarities regarding futility, so CPR is initiated in futile cases for legal reasons/avoidance of accusation. All cases resuscitation was initiated out of hospital are forwarded to the morgue till the need/demand for a necropsy is clarified. |  |
| Greenland |  |  | The doctor must decide whether to stop resuscitation after considering all relevant factors and making all necessary attempts. |
| Hungary |  |  | Early temrimantion if: - the effort seems futile - the effort is inappropriate because of the underlying medical condition or the quality of life. Frailty score can be used - ROSC unlikely based on factors like: long down-time, unwitnessed arrest, no by-stander CPR, non-shockable rhythm, low etCO2, cardiac standstill on US. It depends on the patient's age, medical history, and circumstances of cardiac arrest, but it is based on individual judgement. |
| Iceland |  | 1 - Asystole - confirm for at least 60 sec: - asystole in different leads - apnea - no pulse  2 - Rigor/Livor mortis Decapitation or other obvious non-survivable injuries. |  |
| Ireland |  |  | Pre Covid only asystole could be terminated in the field. During Covid this was extended to PEA with online physician support, this has been retained. |
| Italy |  | The termination of resuscitation in our EMS system is based on international guidelines. After 20-30 minutes of absent ROSC the physician decides to stop resuscitation. We don't have a ECLS program ready and so the termination of rescuscitation is in the prehospital setting. |  |
| Kosovo |  |  | In cases where the patients have no signs of life, despite the treatment for 30-40 min. |
| Luxembourg |  |  | Varies from one doctor to another but in general: rigor mortis, history, very advanced age (no defined limit). It is the decision of the doctor on scene to decide to refrain from resuscitation according to the medical condition of the patient, sometimes age, medical directives, no flow times, arrest witnessed or not.. The anesthetist stops resuscitation when he decides to do so. (if started, it usually lasts at least 30 minutes). As on every cardiac arrest a physician is sent, he makes the decision to terminate the resuscitation. Unless obvious signs of death (rigidity, lesions incompatible with life,...) firefighters or EMT are not allowed to terminate. |
| Malta |  |  | We do not have a guideline on this - it depends on on-site-medical control and the decision is up to the prehospital emergency medicine consultant. Onsite medical control by prehospital emergency medical consultant, if not resuscitation is not terminated in a prehospital setting and the patient is brought to the resuscitation room of the emergency department. |
| Netherlands |  | The ambulance nurse decides within the framework of the medical manager ambulance care and the existing protocol. Ambulance nurses and resuscitation teams (without physician) can only decide to stop resuscitation when this is medical-technical not feasible. After 6 blocks of ALS in adults, and no signs of life, paramedics may terminate resuscitation.  In the Netherlands, termination of resuscitation is considered after 20 minutes of ALS resuscitation if no shockable rhythm persists and if there is no evidence of possible reversible causes. After these 20 minutes, a decision is made together with the team to stop if further action does not seem to be medically useful. | We have standard protocols, such as 20 minutes of ALS. But sometimes depending on patient conditions, or the will of the patient (not to be resuscitated ) or depending on the view of the HEMS physician if present, one can deviate to the protocol. In children the decision to stop resuscitation is made by the HEMS physician. |
| Norway |  |  | We tend to continue rescucitation as long as there is a shockable rythm or until other reversible causes are adressed. If the initial rythm is PEA ("true" PEA when a low-flow state is excluded) or asystoli we will tend to terminate earlier, taking into account down-time, if it was unwitnessed, age and comorbidities. The same is true if an initial shockable rythm converts to asystoli or PEA. If the pasient is old, multimorbid, and after a long time,the pasient does not get ROSC. i do and Ultrasound to see if the heart is still. A total evaluation by the doctor in charge. |
| Poland |  |  |  |
| Portugal |  |  | If an ALS unit with a physician on board is at the scene, the medical doctor can decide to end the resuscitation. If not, the patient is transported to an ED, under CRP. |
| Russia |  | Literal translation of the Clause 66 of the Federal law no. 323-FZ: Resuscitation measures should be terminated if they are considered absolutely unpromising, namely: 1) when a person is declared dead on the basis of brain death, including against the background of ineffective use of the full range of resuscitation measures aimed at maintaining life; 2) if resuscitation measures aimed at restoring vital functions are ineffective within thirty minutes; 3) if the newborn does not have a heartbeat after ten minutes from the start of resuscitation measures (artificial ventilation, chest compressions, administration of medications). |  |
| Serbia |  |  | Decision making procesa Is solely up to physician on site. The decision Is based on guidelines, medical directives and insight in patient 's medical hystory. |
| Slovenia |  |  | The decision to terminate the resuscitation is made by the on-site physician. His decision is made on the facts gathered from the bystanders, medical records (if available on-site), and the guidelines are included in that decision (for example, etCO2 less than 10 mmHg after 20 min of resuscitation). Most OHCA resuscitations without ROSC in our country are terminated on-site/in the field. Only a physician can make a decision about termination of resuscitation. Decision is based on guidelines, circumstances, available information about the patient on scene etc. The decision is made by the physician on scene individually for each patient. For example, in the case of palliative care or when the resuscitation attempt would be futile, the resuscitation attempts would be refrained. |
| Spain |  |  | Depends on attending physician following guidelines: non witnessed arrest, asystole since onset, ie no shockable rhythm, past illness and history, review of Hs and Ts etc. |
| Sweden |  | The EMS nurses can according to guidelines termianate rests. EMS physcisans can decide without use of guidelines decide to terminate resus. Ground EMS are avcording to SOPs allowed to terminate CPR if no ROSC after 20 minutes CPR. | SOPs based on guidelines in combination with clinical judgement by HEMS physician. The EMS nurses can according to guidelines termianate rests. EMS physcisans can decide without use of guidelines decide to terminate resus. Physician based HEMS ca terminate earlier at the descretion of the providimg physician (for example high age/comorbidities, unvittenessed carrdiac arrest, asystolia, low ETCO2 after long CPR and/or no cardiac movement on ultrasound etc). Physician manned units are allowed to refrain from cpr according to multifacoral deciscion making (for example unwitnwssed cardiac arrest on a patient with high age and comorbidities). |
| Switzerland |  |  | Decisions are made on a case-by-case basis by the prehospital physician handling the cardiac arrest case. Many factors are taken into account (no-flow time, low-flow time, comorbidities, presumed etiology, EtCO2, cardiac rhythm). In case of doubt, a senior specialist physician is on call 24/7 and can go onsite as a reinforcement. If resuscitation is deemed futile, physicians and specialist physicians can decide to refrain from initiating CPR. |
| United Kingdom |  |  | Clinicians can also use professional judgement if a patient is in advanced stages of irreversible disease process likely to lead to death. Guidance and advice are provided for patients who do not fit this criterion but supports a clinical decision more than provides rules. |
| **North America** |  |  |  |
| Canada |  | For medical cardiac arrest: If BLS crew at scene - Age ≥18 years - vital signs absent - Arrest not witnessed by EMS AND No ROSC AND No defibrillation delivered with BLS crew If ALS crew at scene - as above AND unwitnessed by bystander AND no bystander CPR |  |
| Mexico |  | Finishing algorithms on scene with no ROSC - After complete protocol (Based on AHA guidelines) is done without success then the Online Medical Control can approve termination of resuscitation. |  |
| United states (California) |  | 1. Cardiac arrest not witnessed by EMS 2. No return of spontaneous circulation (even transiently) 3. Asystole after approximately 20 minutes of resuscitation |  |
| United states (Colorado) |  | Termination of Resuscitation (TOR) Indications a. Provider discretion, the timeframe to Termination of Resuscitation (TOR) can be extended. • Decision to terminate/withhold resuscitation MUST meet the respective criteria. i. ADULTS:  o All Bokutoh criteria must be met to withhold resuscitation • Unwitnessed by anyone, age >73 years old, non-shockable rhythm o All BLS TOR criteria must be met to terminate at the 8-minute mark • No EMS witness, no ROSC, no defibrillation o Patients not meeting BLS TOR criteria should have a total of 37 minutes of resuscitation efforts. For BLS-only providers, contact Base for TOR when all of the following  criteria met:  i. No AED shock advised  ii. No ROSC  iii. 20 minutes of quality CPR with patent airway or: 1. Arrest unwitnessed by either EMS or bystanders  2. No bystander CPR before EMS arrival |  |
| United states (Florida) |  | Resuscitation efforts may be terminated if ALL of the following criteria are met: a) The patient was a non-witnessed cardiac arrest and, b) The patient maintained a non-shockable EKG reading throughout the resuscitation and, c) The patient has no Return of Spontaneous Circulation (ROSC) after 20 minutes of resuscitation and, d) The patient has an advanced airway (ET Tube) in place and an ETCO2 reading < 10 after 20 minutes of resuscitation. |  |
| United states (Illinois) |  | No return of spontaneous circulation has been achieved after at least 20 minutes of prehospital resuscitation as per Cardiac Arrest Management (ICCA) Protocol – BLS/ALS. B. Initial rhythm is asystole or pulseless electrical activity (PEA). 1. Confirmed in two different leads. 2. For patients in PEA, bradycardic rhythms with a wide QRS complex are more consistent with terminal cardiac rhythms. Faster, narrow QRS complex rhythms may indicate ROSC. C. IV or IO access is established. 1. Epinephrine 1 mg IV every 5 minutes. 2. 3 total doses of Epinephrine have been administered. D. Advanced airway established. 1. Supraglottic airway or endotracheal tube. E. End Tidal CO2 (ETCO2) capnography attached with number and waveform reading. 1. ETCO2 values persistently less than 10 mmHg or decreasing (downward trend) of more than 25% despite resuscitation indicate a poor prognosis. II. If all of the above criteria are met: A. Contact Medical Control B. Request termination of resuscitation from ECP or ECRN. Patients are worked for a minimum of 20min on scene unless determined to have had an obvious death criteria or have a valid DNR/POLST. Occasionally if family decides resuscitation not within goals of care, crews can call for orders to stop resuscitation sooner. The ToR decision should take into account if an arrest was witnessed, the initial and subsequent rhythms, the ETCO2, and any response to interventions. |  |
| United states (Intermountain) |  | Generally if resuscitation has begun by bystanders then EMS continues resuscitation attempts. Signs of life are evaluated by the team, and a team decision is made about beginning or ending resuscitation efforts. |  |
| United states (Massachusetts) |  | Termination of resuscitation is a medical director option. There is a standard protocol for it and requires online medical control real time approval if the medical director approves it for use at all in the system |  |
| United states (Michigan) |  | If the resuscitation has been unsuccessful after at least 30 minutes (ALS time without ROSC), the resuscitation may be terminated with the permission of medical control. If persistent Ventricular Fibrillation, prompt emergency transport will be initiated. Once resuscitation is initiated by ALS or LALS it may be terminated only at the direction of medical control. ROSC, i.e. return of a pulse resets the 30 minute clock and transport should be initiated. Medics follow protocol. If they have questions, they call online medical control. If there is an on-scene EMS physician/fellow that person serves as medical control. |  |
| United states (Midwest) |  | On scene EMS providers determine if CPR should be started and have specific termination criteria that can be implemented in consultation with a physician if indicated. |  |
| United states (Mississippi) |  |  |  |
| United states (Missouri) |  | 30 minutes of cardiac arrest care and checklist for termination. Criteria to terminate resuscitation: i. Initial rhythm is asystole or AED interpretation of No Shock Advised,  after 20 minutes of BLS or ALS care. ii. Any resuscitation after 30 minutes of BLS or ALS care. iii. Presentation of a valid DNR iv. Contact Medical Control for all termination of resuscitation.  1. If unable to contact medical control due to no communication  capability (radio out of range, no cellular service, etc), ensure  below criteria are met: a. 20-30 minutes of BLS or ALS care has been administered per  i or ii above  b. At least 5 minutes have elapsed since most recent  administration of any medication (besides oxygen and  normal saline), and c. asystole or PEA with a rate of <= 45 on the monitor. |  |
| United states (Nebraska) |  | Missing |  |
| United states (New England) |  | Massachusetts Ems protocols |  |
| United states (New Jersey) |  | Termination of resuscitation will only be initiated in the following situations and after assuring good quality CPR: 1. Return of Spontaneous Circulation (ROSC) occurs. 2. Advanced Life Support directs termination based on treatment and medical command order. 3. Patient hand-off to receiving facility. |  |
| United states (Ohio) |  | Medics may terminate resuscitative efforts and not transport patients under active CPR if all of the following exist: A. Good contact between the paramedic unit and the medical control physician. B. Successful airway management and medication administration consistent with other protocols in this document. C. At least 30 minutes of resuscitative efforts D. NO sustained return of spontaneous circulation at any time (palpable pulse greater than 60 beats per minute for at least one five-minute period). E. NO spontaneous respiration: eye opening, motor response, or other neurologic activity at the time stopping resuscitation is contemplated. F. The cardiac rhythm is NOT persistent or recurrent ventricular fibrillation or ventricular tachycardia. G. All paramedics and the medical control physician agree with termination of ACLS. H. The suspected cause of the cardiac arrest must be something other than hypothermia, electrocution, lightning strike. I. While patients who are pregnant may not themselves benefit from longer resuscitation, the unborn fetus may benefit from emergency c-caesarian section. Consequently, it is recommended to transport pregnant patients even if there has been no return of spontaneous circulation. |  |
| United states (Oklahoma) |  | Protocol specified, dysrhythmia and time specific variables in the decision matrix to terminate resuscitation, with some requiring on-line medical consult. |  |
| United states (Oregon) |  | In addition to the conditions listed above under Death in the Field, a medical patient should generally be declared dead if: 1. ECG shows asystole or agonal rhythm upon initial monitoring, and after at least two lead changes, the patient, in the paramedic's best judgment, would not benefit from resuscitation: a. The PIC should determine DIF and notify Law Enforcement;  - OR - b. Begin BLS procedures, and contact OLMC with available patient history,  current condition, and with a request to discontinue resuscitation. 2. If after the airway is established and the asystole protocol has been exhausted the patient persists in asystole, (confirm in 3 leads) consider termination of efforts. The PIC may declare the patient to be dead in the field. 3. The patient who has PEA/Asystole and has not responded to the initial cycle of  ACLS may be determined to be dead at the scene after appropriate consultation with OLMC. 4. All patients in VF/VT should be treated and transported unless a valid, signed DNR is present |  |
| United states (Pennsylvania) |  |  |  |
| United states (Tennessee) |  | We use physiologic parameters to determine when to cease efforts. |  |
| United states (Texas) |  | TOR after Unsuccessful resuscitative efforts following: CONFIRMED EFFECTIVE VENTILATION + HIGH QUALITY CPR + ACLS DRUGS. contraindication: rosc at any point, witnessed arrest, cpr induced consciousness at any point, EMS witnessed arrest, abrupt increase etco equal to or more than 10, hypothermia, family request for continuation, public place, or pregnancy. Contraindications for use of protocol  Any of the following are present,  → EMS witnessed arrest → Return of Spontaneous Circulation (ROSC) or presumed ROSC at any point in care → CPR induced consciousness at any point in care → Abrupt ↑ EtCO2 ≥ 10 mmHg ± “pulses” → Hypothermic patients → Family request for continued efforts → Resuscitation attempted in public view → Pregnancy |  |
| United states (Utah-san Juan) |  | Resuscitation may be terminated in the setting of futility, which can be measured using metrics of time, interventions, or physiologic parameters. |  |
| United states (Virginia) |  | If CPR has been initiated and circumstances arise where the pre-hospital provider believes further resuscitative efforts may not be indicated, cease resuscitation orders may be requested via on-line medical control. • Resuscitation efforts initiated prior to EMS arrival • No return of spontaneous circulation prior to transport • No shockable rhythm during resuscitation • The arrest was not witnessed by EMS personnel • The arrest was not witnessed by bystander • No bystander CPR • > 25 minutes of CPR • ETCO2 is < 10mmHg |  |
| United states (Washington) |  | Termination of Resuscitation - Resuscitation may be terminated in the field if any of the following criteria are met:  a. Unwitnessed arrest, no immediate CPR, non-shockable rhythm.  b. The electrical rhythm is asystole or pulseless electrical activity and has not responded to the treatment protocol for Asystole or PEA.  i. Asystole/PEA must be confirmed in two leads  ii. No respiratory effort is present  c. The patient is in a non-perfusing rhythm for an extended time (~30 min or more), and ETCO2 remains at 10 mm/hg or less.  d. No respiratory effort is present.  e. Hypothermia prior to arrest is not present.  f. DNR is presented after resuscitation is initiated.  g. Contact medical control for advice or concurrence.  h. Consider a respectful pause at the end of resuscitation. |  |
| United states (Wisconsin) |  | Standardized protocols |  |
| **Oceania** |  |  |  |
| Australia |  | Resuscitation may be stopped under the following circumstances (regardless of patient age): 1. if, during the course of the resuscitation, it becomes apparent that it should not have been commenced. OR 2. resuscitation has been ongoing for at least 20 minutes* (excluding bystander CPR prior to paramedic arrival) AND criteria for determining life extinct are met (see below) AND ECG shows asystole or PEA (particularly if markedly slow and wide) * In the traumatic arrest patient, if no sustained ROSC after 10 minutes of management, stopping resuscitation should be seriously considered, especially if in asystole. Termination is guided by clinical practice guidelines, supplemented in complex cases by the availability of an online physician. |  |
| New Zealand |  | If the arrest was unwitnessed and no shock is advised, the prognosis is very poor and it is appropriate to stop resuscitation if there are no signs of ROSC 20 minutes after the onset of resuscitation by ambulance personnel. For other circumstances it is appropriate to stop resuscitation if there are no signs of ROSC 40 minutes after the onset of resuscitation by ambulance personnel. | There is no absolute time at which it is possible to say that further resuscitation is futile. Stopping resuscitation requires clinical judgement on the likelihood of survival, considering the following: The cause of the cardiac arrest. Whether or not the cardiac arrest was witnessed. Whether or not there was bystander CPR. The response time. The initial rhythm. The total estimated time in cardiac arrest. Whether ROSC has occurred at any time. The patient's comorbidities. Deciding to stop resuscitation: Paramedics and ICPs It is appropriate to stop resuscitation 20 minutes after the onset of resuscitation by ambulance personnel in poor prognosis scenarios. It is appropriate to stop resuscitation 40 minutes after the onset of resuscitation by ambulance personnel in good prognosis scenarios. It is appropriate to stop resuscitation earlier than described above, if it becomes clear that it was inappropriate to have commenced resuscitation, or the rhythm has deteriorated into asystole for more than a few minutes despite resuscitation. |
| **South America** |  |  |  |
| Brazil |  |  | There is no clear recommendation on when to cease efforts during CPR. The determination to stop efforts is difficult, and some situations can help in deciding not to initiate CPR or to determine the end of CPR efforts:  • When there are signs of obvious death or an irreversible clinical condition; • Persistent asystole despite 20 minutes of advanced life support, in the absence of any reversible cause; • Unwitnessed cardiac arrest of cardiac origin with an initial non-shockable rhythm, where the risk of harm to the patient from ongoing CPR likely outweighs any benefit (absence of return of spontaneous circulation, severe chronic comorbidity, very poor quality of life prior to cardiac arrest).  Some criteria such as pupil size/response, previous comorbidities, probable cause of cardiac arrest, and time without receiving basic life support should not solely guide the decision-making process. |
| Chile |  |  |  |
| Peru |  |  | Nothing is defined, it depends on the medical decision. |

**Supplementary file 6 – Termination of resuscitation table**

| **Country**  **(Region)** | **The instructions that determine the practice regarding termination of resuscitation** | **The authority that can decide to terminate resuscitation** | **The practice regarding**  **termination of resuscitation** | **The variations in the practice regarding termination of resuscitation** |
| --- | --- | --- | --- | --- |
| **Africa** |  |  |  |  |
| Kenya | Standardized protocols, Guidelines, Medical directives, Online medical control | Physician (specialist) on scene, Physician (specialist) via phone, Physician on scene, Physician at a hospital, Paramedic, Emergency medical technician, Nurse | Specific criteria/guidelines (e.g. time specific, guideline, advanced directives) | EMS-system organization, Response time, Differences in work-flow, In patients eligible for organ donation, In patients eligible for extracorporeal, cardiopulmonary resuscitation (eCPR), In patients from nursing homes, For specific age groups |
| Rwanda | No termination of resuscitation in the prehospital setting | N/A | No termination of resuscitation at scene | Geographical allocations |
| Sierre Leone | Standardized protocols,  Guidelines,  Medical directives,  On-site medical control | Physician (specialist) on scene  Physician on scene  Physician at a hospital  Paramedic | Discretion of the care provider | EMS-system organization,  Response time |
| South Africa | Standardized protocols, Rules, Guidelines, Medical directives, Online medical control, Decision-making without the use of guidelines or protocols, On-site medical control | Physician (specialist) on scene, Physician (specialist) via phone, Physician on scene, Physician at a hospital, Paramedic, Emergency medical technician, Emergency Care Practitioner | Specific criteria/guidelines (e.g. time specific, guideline, advanced directives) - Emergency medical technician.  Discretion of the care provider - Paramedic,  Physician via phone. | EMS-system organization, Response time, Differences in work-flow, Differences in treatment facilities, Level of training, Rural versus urban areas, In patients eligible for organ donation, In patients eligible for extracorporeal cardiopulmonary resuscitation (eCPR), In patients from nursing homes, In patients from different geographical locations, For specific age groups |
| Tunisia | Guidelines, Medical directives,  Decision-making without the use of guidelines or protocols On-site medical control | Physician (specialist) on scene,  Physician on scene | Discretion of the care provider | Differences in treatment facilities,  In patients from nursing homes,  For specific age groups |
| Uganda | Guidelines,  Decision-making without the use of guidelines or protocols,  On-site medical control,  Socio-cultural influence | Paramedic,  Nurse,  Police- or firemen,  Laymen | Discretion of the care provider | Response time,  Differences in treatment facilities, Differences in first responders,  Rural vs. Urban,  Limitation of transportation |
| **Asia** |  |  |  |  |
| China | No termination of resuscitation in the prehospital setting. | Physician at a hospital, No termination of resuscitation in the prehospital setting. | No termination of resuscitation at scene | EMS-system organization, Response time, Differences in work-flow, Differences in treatment facilities, In patients eligible for extracorporeal cardiopulmonary resuscitation (eCPR), In patients from different geographical locations, For specific age groups. |
| India | On-site medical control | Physician (specialist) on scene, Emergency medical technician | Discretion of the care provider | Availability of physicians |
| Japan | Standardized protocols, Rules, No termination of resuscitation in the prehospital setting. | Physician at a hospital, No termination of resuscitation in the prehospital setting. | No termination of resuscitation at scene | EMS-system organization |
| Jordan | Rules, Guidelines, Decision-making without the use of guidelines or protocols (the medical direction is still limited), Internal regulations | Physician at a hospital, No termination of resuscitation in the prehospital setting. | No termination of resuscitation at scene | No variation described |
| Malaysia | Guidelines,  Medical directives,  Online medical control,  On-site medical control | Physician (specialist) on scene,  Physician (specialist) via phone,  Paramedic | Specific criteria/guidelines (e.g. time specific, guideline, advanced directives) | No variation described |
| Philippines | Standardized protocols, Online medical control, No termination of resuscitation in the prehospital setting. | Physician at a hospital, No termination of resuscitation in the prehospital setting. | No termination of resuscitation at scene | EMS-system organization, Response time, Differences in treatment facilities, In patients from different geographical locations, In patients eligible for organ donation, For specific age groups. |
| Qatar | Standardized protocols, Guidelines,  Medical directives | Paramedic,  Nurse | Specific criteria/guidelines (e.g. time specific, guideline, advanced directives) | No variation described |
| Saudi Arabia | No termination of resuscitation in the prehospital setting. | Physician at a hospital, No termination of resuscitation in the prehospital setting. | No termination of resuscitation at scene | Differences in work-flow, In patients from different geographical locations |
| Singapore | Standardized protocols, Rules, Guidelines, Online medical control | Physician (specialist) via phone, Paramedic | Specific criteria/guidelines (e.g. time specific, guideline, advanced directives) | No variation described |
| South Korea | Standardized protocols, Online medical control On-site medical control | Physician (specialist) on scene, Physician (specialist) via phone, Physician on scene, Physician at a hospital | Discretion of the care provider | No variation described |
| Sri Lanka | Medical directives,  Online medical control | Physician (specialist) via phone, Physician at a hospital | Discretion of the care provider | No variation described |
| Taiwan | Rules, Guidelines, No termination of resuscitation in the prehospital setting. | No termination of resuscitation in the prehospital setting. | No termination of resuscitation at scene | No variation described |
| Thailand | Standardized protocols, Guidelines, Online medical control, No termination of resuscitation in the prehospital setting. | Physician at a hospital, No termination of resuscitation in the prehospital setting. | No termination of resuscitation at scene | No variation described |
| Turkey | Standardized protocols,  guidelines,  On-site medical control | Physician (specialist) via phone, Physician on scene | Discretion of the care provider | Differences in work-flow,  Differences in treatment facilities,  In patients eligible for organ donation,  In patients eligible for extracorporeal cardiopulmonary resuscitation (eCPR) |
| United Arab Emirates | No termination of resuscitation in the prehospital setting | N/A | No termination of resuscitation at scene | No variation described |
| **Central America** |  |  |  |  |
| Costa Rica | Decision-making without the use of guidelines or protocols | Physician (specialist) via phone | Discretion of the care provider | No variation described |
| **Europa** |  |  |  |  |
| Austria | Rules, Guidelines, Medical directives,  On-site medical control,  Decision-making without the use of guidelines or protocols | Physician (specialist) on scene,  Physician (specialist) via phone,  Physician on scene,  Physician at a hospital | Discretion of the care provider | In patients eligible for organ donation, In patients eligible for extracorporeal cardiopulmonary resuscitation (eCPR), In patients from nursing homes, For specific age groups |
| Belgium | Standardized protocols,  Rules,  Guidelines | Physician (specialist) on scene,  Physician on scene | Discretion of the care provider | Response time, Differences in treatment facilities, In patients eligible for eCPR, In patients from nursing homes |
| Bosnia and Herzegovina | Standardized protocols,  Rules,  Guidelines | Physician (specialist) on scene | Missing | EMS-system organization, Response time, Differences in work-flow, Differences in treatment facilities, In patients from different geographical locations, For specific age groups |
| Croatia | Guidelines, on-site medical control | Physician (specialist) on scene, Physician on scene | Discretion of the care provider | No variation described |
| Czech Republic | Standardized protocols, Rules, Guidelines, Medical directives On-site medical control | Physician (specialist) on scene, Physician on scene, Physician at a hospital | Discretion of the care provider | In patients eligible for organ donation, In patients eligible for extracorporeal cardiopulmonary resuscitation (eCPR), In patients from nursing homes, In patients from different geographical locations, For specific age groups |
| Denmark | Standardized protocols,  Rules,  Guidelines,  Medical directives,  Decision-making without the use ofguidelines or protocols,  On-site medical control | Physician (specialist) on scene,  Physician (specialist) via phone,  Physician on scene, Physician at a hospital | Discretion of the care provider | EMS-system organization (Availability of physicians),  Response time,In patients eligible for extracorporeal cardiopulmonary resuscitation (eCPR),In patients from different geographicallocations,  For specific age groups. |
| Finland | Standardized protocols, Rules, Guidelines, Medical directives, Online medical control, On-site medical control | Physician (specialist) on scene, Physician (specialist) via phone, Physician on scene, Physician at a hospital, Paramedic (in some parts of the country) | Discretion of the care provider | EMS-system organization, Response time, In patients eligible for extracorporeal cardiopulmonary resuscitation (eCPR), In patients from nursing homes. |
| France | Standardized protocols  On-site medical control | Physician (specialist) on scene,  Physician (specialist) via phone | Discretion of the care provider | In patients eligible for organ donation,  In patients eligible for extracorporeal  cardiopulmonary resuscitation (eCPR),  In patients from nursing homes,  For specific age groups |
| Germany | Rules, Medical directives, Decision-making without the use of guidelines or protocols, On-site medical control | Physician (specialist) on scene, Physician on scene, Physician at a hospital | Discretion of the care provider | EMS-system organization, Response time, Differences in workflow, Rural versus urban areas In patients eligible for extracorporeal cardiopulmonary resuscitation (eCPR), For specific age groups. |
| Greece | Guidelines, On-site medical control | Physician (specialist) on scene, Physician (specialist) via phone, Physician on scene, Physician at a hospital | Specific criteria/guidelines (e.g. time specific, guideline, advanced directives) | In patients eligible for organ donation,  For specific age group. |
| Greenland | Rules, Guidelines, Online medical control, On-site medical control | Physician (specialist) via phone, Physician at a hospital | Discretion of the care provider | EMS-system organization, Response time, Differences in work-flow, Differences in treatment facilities, In patients from different geographical locations. |
| Hungary | Standardized protocols, Guidelines, Decision-making without the use of guidelines or protocols | Physician (specialist) on scene, Physician (specialist) via phone, Physician on scene, Physician at a hospital | Discretion of the care provider | No variation described |
| Iceland | Standardized protocol, Rules, Guidelines, Online medical control, On-site medical control | Physician (specialist) on scene, Physician (specialist) via phone, Physician on scene, Physician at a hospital, Paramedic, Emergency medical technician | Specific criteria/guidelines (e.g. time specific, guideline, advanced directives) | EMS-system organization, Rural versus urban areas, Differences in available personnel |
| Ireland | Standardized protocols,  Guideline,  Medical directives,  Online medical control | Physician (specialist) via phone,  Paramedic,  Emergency medical technician | Specific criteria/guidelines (e.g. time specific, guideline, advanced directives) | No variation described |
| Italy | Guidelines, Medical directives, On-site medical control | Physician (specialist) on scene, Physician on scene, Physician at a hospital | Specific criteria/guidelines (e.g. time specific, guideline, advanced directives) | No variation described |
| Kosovo | Standardized protocols,  Rules,  Guidelines | Physician (specialist) on scene  Physician on scene,  Physician at a hospital,  Nurse | Discretion of the care provider | No variation described |
| Luxembourg | Medical directives, Decision-making without the use of guidelines or protocols, On-site medical control | Physician (specialist) on scene, Physician on scene | Discretion of the care provider | In patients eligible for extracorporeal cardiopulmonary resuscitation (eCPR). |
| Malta | Standardized protocols, guidelines,  On-site medical control | Physician (specialist) on scene, Physician on scene, Physician at a hospital | Discretion of the care provider | No variation described |
| Nederland | Standardized protocols, Rules, Guidelines, Medical directives, Online medical control, Decision-making without the use of guidelines or protocols, On-site medical control | Physician (specialist) on scene, Physician (specialist) via phone, Physician on scene, Paramedic, Emergency medical technician, Nurse | Specific criteria/guidelines (e.g. time specific, guideline, advanced directives) - Paramedic, Emergency medical technician, Nurse.  Discretion of the care provider - Physician and HEMS doctor. | In patients eligible for organ donation, In patients eligible for extracorporeal cardiopulmonary resuscitation (eCPR), In patients from nursing homes, For specific age groups. |
| Norway | Standardized protocols, Rules, Guidelines, Medical directives, Online medical control, Decision-making without the use of guidelines or protocols, On-site medical control | Physician (specialist) on scene, Physician (specialist) via phone, Physician on scene, Physician at a hospital, Paramedic | Discretion of the care provider | In patients eligible for extracorporeal cardiopulmonary resuscitation (eCPR),  In patients from nursing homes, For specific age groups. |
| Poland | Standardized protocols, Guidelines | Physician (specialist) on scene, Physician on scene | Missing | No variation described |
| Portugal | Standardized protocols,On-site medical control | Physician on scene,  Physician at a hospital | Discretion of the care provider | In patients eligible for organ donation |
| Russia | The Federal law | Physician (specialist) on scene, Physician on scene,  Feldsher (mid-level EMS provider) | Specific criteria/guidelines (e.g. time specific, guideline, advanced directives) | For specific age groups (new-born) |
| Serbia | Guidelines, Medical directives | Physician (specialist) on scene,  Physician on scene | Discretion of the care provider | No variation described |
| Slovenia | Guidelines, Medical directives | Physician (specialist) on scene,  Physician on scene | Discretion of the care provider | No variation described |
| Spain | Guidelines, Decision-making without the use of guidelines or protocols, On-site medical control | Physician (specialist) on scene, Physician (specialist) via phone, Physician on scene, Physician at a hospital | Discretion of the care provider | Differences in workflow, In patients eligible for extracorporeal cardiopulmonary resuscitation (eCPR), |
| Sweden | Standardized protocols, Guidelines, Medical directives, Online medical control, On-site medical control | Physician (specialist) on scene, Physician (specialist) via phone, Physician on scene, Physician at a hospital,  Nurse | Discretion of the care provider | In patients eligible for organ donation, In patients eligible for extracorporeal cardiopulmonary resuscitation (eCPR), In patients from nursing homes |
| Switzerland | Guidelines, Medical directives, Decision-making without the use of guidelines or protocols, On-site medical control | Physician (specialist) on scene, Physician on scene, Paramedic | Specific criteria/guidelines (e.g. time specific, guideline, advanced directives) - EMS Nurse.  Discretion of the care provider - Physician and HEMS doctor. | EMS-system organization, In patients eligible for extracorporeal cardiopulmonary resuscitation (eCPR), In patients from nursing homes, For specific age groups. |
| United Kingdom | Standardized protocols, Medical directives, Decision-making without the use of guidelines or protocols, On-site medical control | Physician (specialist) on scene, Physician on scene, Paramedic | Discretion of the care provider | No variation described |
| **North America** |  |  |  |  |
| Canada | Standardized protocols, Medical directives, Online medical control | Physician (specialist) via phone,  Physician at a hospital | Specific criteria/guidelines (e.g. time specific, guideline, advanced directives) | Transport times to hospital |
| Mexico | Guidelines,  Medical directives,  Online medical control | Physician (specialist) via phone,  Paramedic | Specific criteria/guidelines (e.g. time specific, guideline, advanced directives) | EMS-system organization,  Response time,  Differences in treatment facilities,  Availability of online medical direction and paramedic |
| United States - Midwest | Standardized protocols,  Guidelines,  Medical directives,  Online medical control | Physician (specialist) on scene, Physician (specialist) via phone, Physician on scene, Physician at a hospital, Paramedic | Specific criteria/guidelines (e.g. time specific, guideline, advanced directives) | EMS-system organization, Response time, In patients eligible for extracorporeal cardiopulmonary resuscitation (eCPR), Transport time, For specific age groups |
| United States - Northeast | Standardized protocols, Guidelines, Online medical control | Physician (specialist) via phone, Physician on scene, Physician at a hospital, Paramedic | Specific criteria/guidelines (e.g. time specific, guideline, advanced directives) | EMS-system organization, Differences in work-flow |
| United States - South | Standardized protocols, Guidelines Medical directives, Online medical control | Physician (specialist) on scene, Physician (specialist) via phone, Physician on scene, Physician at a hospital, Paramedic (in some parts of the region), Emergency medical technician (in some parts of the region) | Specific criteria/guidelines (e.g. time specific, guideline, advanced directives)  Discretion of the care provider (in some parts of the region) | For specific age groups, Pregnant patients, In patients eligible for extracorporeal cardiopulmonary resuscitation (eCPR), Hypothermia,  Trauma patients |
| United States - West | Standardized protocols, Online medical control, On-site medical control | Physician (specialist) on the scene, Physician (specialist) via phone, Physician on scene, Physician at a hospital, Paramedic (in some parts of the region), Emergency medical technician (in some parts of the region) | Specific criteria/guidelines (e.g. time specific, guideline, advanced directives) | In patients eligible for organ donation, For specific age groups, Response time, The following patients found pulseless and apnoeic warrant resuscitation efforts beyond 20 minutes and SHOULD be transported: a) Hypothermia b) Drowning with hypothermia and submersion < 60 minutes c) Pregnant patient with estimated gestational age ≥ 23 weeks (obvious pregnancy) d) Lightning strike / significant electrocution |
| **Oceania** |  |  |  |  |
| Australia | Standardized protocols, Rules, Guidelines, Medical directives, Online medical control | Physician (specialist) on scene, Physician (specialist) via phone, Physician on scene, Paramedic, Nurse | Specific criteria/guidelines (e.g. time specific, guideline, advanced directives) | No variation described |
| New Zealand | Guidelines, Medical directives, Online medical control, Decision-making without the use of guidelines or protocols | Physician (specialist) via phone, Paramedic, Emergency medical technician | Specific criteria/guidelines (e.g. time specific, guideline, advanced directives) - Emergency medical technician.  Discretion of the care provider - Paramedic, Physician via phone. | No variation described |
| **South America** |  |  |  |  |
| Brazil | Standardized protocols | Physician (specialist) on scene,  Physician on scene | Discretion of the care provider | No variation described |
| Chile | Don't know | Physician (specialist) on scene,  Physician (specialist) via phone,  Physician on scene,  Physician at a hospital,  Paramedic | Missing | Don't know |
| Peru | Decision-making without the use of guidelines or protocols | Physician (specialist) on scene,  Physician on scene,  Paramedic,  Emergency medical technician,  Nurse,  Police- or firemen | Discretion of the care provider | No variation described |

If at least one participant from a country provided an answer, it is included in the table.

**Supplementary file 7 – Refraining from resuscitation (matrix)**

|  | **Refraining from resuscitation** | | | | |
| --- | --- | --- | --- | --- | --- |
|  | **Patients with obvious clinical signs of irreversible death** | **Patients with acceptable advanced directives** | **Performing CPR would endanger the lives or safety of the EMS personnel** | **Non-specific criteria/judgment** | **No refraining from resuscitation in the prehospital setting** |
| **Africa** |  |  |  |  |  |
| Kenya | You refrain in cases of cardiac arrest more than 12 hours ago and exhibiting all signs of death Resuscitation is recommended for all patients except those who show obvious signs of death such as: 1. Are already in the decomposition stage 2. Have rigor mortis 3. Are decapitated | No resuscitation attempts will be made if there is a valid DNR presented to the medics. | Termination is also considered when the scene is deemed unsafe to continue. |  |  |
| Rwanda |  |  |  |  | in Rwanda we do not prioritize and even refrain during resuscitation in EMS system. |
| Sierra Leone | Prehospital healthcare professionals can refrain from resuscitation if obvious clinical signs of irreversible death are present |  |  |  |  |
| South Africa | When obviously dead. Including disfigurement, lividity and rigor mortis Rigor mortus Mortal lividity decapitation Burnt beyond recongition to name a few |  |  |  |  |
| Tunisia | Prehospital healthcare professionals can refrain from resuscitation if obvious clinical signs of irreversible death are present | Prehospital healthcare professionals are legally obliged to adhere to a DNACPR | There have been occasional outbreaks of highly infectious diseases like ebola which may require isolation. Due to scarcity of PPE, patients who are suspected to have such disease are not usually resuscitated |  |  |
| Uganda | Prehospital healthcare professionals can refrain from resuscitation if obvious clinical signs of irreversible death are present | Prehospital healthcare professionals are legally obliged to adhere to a DNACPR |  |  |  |
| **Asia** |  |  |  |  |  |
| China | Unless the conditions fit the criteria of obvoius death, our EMS will perform BLS to all cardiac arrest cases, until they reach public hospital AEDs |  |  |  |  |
| India |  |  | The rescuer decision is usually based on: Rescuer exhaustion The scene becomes unsafe. Unsafe scenario, The scene becomes unsafe |  |  |
| Japan | Patients who are clearly dead (social death) or have been declared dead by a physician are not eligible for resuscitation or transport. 1 Patients who can be considered socially accepted as dead due to conditions such as amputation of the head or trunk 2 Patients who can be considered socially acceptable as dead due to conditions such as rigidity of the extremities and death spots. 3 Patients with cardiac arrest who are considered to be dead by socially accepted standards due to injuries sustained from flames at a fire scene, etc. 4 Patients with cardiac arrest who are considered to be dead by socially accepted standards due to traumatic brain extrusion outside the skull 5 Patients with cardiac arrest who cannot continue CPR due to traumatic injuries to the face or thorax, and who are considered to be dead by socially accepted standards. |  |  |  |  |
| Jordan | Prehospital healthcare professionals can legally refrain from resuscitation if obvious clinical signs of irreversible death are present |  |  |  |  |
| Malaysia | when there is obvious signs of death like: decomposition, rigor mortis, dependant lividity, decapitation, pre-term infant <20 weeks gestation, fetal maceration, evisceration of major organs, incineration | a valid DNR order |  |  |  |
| Philippines | State of decomposition. Decapitation. The EMTs who are confronted with an arrested patiens and relatives to do want to start resuscitative measures anymore or EMTs determine that there are signs of irreversible death example rigor mortis or dependent lividity. |  |  |  |  |
| Qatar | The legislation states generally that CPR should not be commenced in cases of "signs of biological death" and "consequences of acute injury incompatible with life".  When there is obvious signs of death like: > decomposition > rigor mortis > dependant lividity > decapitation > a valid DNR order > pre-term infant <20 weeks gestation > fetal maceration > evisceration of major organs > incineration | when there is….: >  > a valid DNR order > |  |  |  |
| Saudi Arabia | Prehospital healthcare professionals can legally refrain from resuscitation if obvious clinical signs of irreversible death are present | Prehospital healthcare professionals are legally obliged to adhere to a DNACPR |  |  |  |
| Singapore | Only the patients with decomposed body, decapitation etc with no signs of life will not be resuscitated. | Prehospital healthcare professionals are legally obliged to adhere to a DNACPR |  |  |  |
| South Korea | When there are definitive clinical signs of death - Post-mortem rigidities (check 2 or more joints) - Rigor mortis Decapitation (cutting off the head) - Trunk amputation (cutting of the torso) - Brain parenchymal herniation due to trauma - Corruption (smelling, discoloration, deformation). Obvious clinical signs of death are categorised by EMS protocol. If a case is indicated with this criteria, the provider can refrain the CPR and het conformation from medical director via telecommunication system. | A medical indication that you do not want to perform CPR or do not attempt resuscitation (DNAR: Do Not Attempt Resuscitation) When a legal representative expresses his/her intention to refuse resuscitation Patients for whom a decision has been made to discontinue life-sustaining treatment in accordance with the Life-Sustaining Treatment Decision Act. | Situations that pose a serious risk to paramedics performing CPR. Circumstances in which resuscitation can be stopped at the scene. - When paramedics are exposed to an environment in which it is difficult to continue resuscitation |  |  |
| Sri Lanka |  |  |  |  | In general we are not refraining for the resuscitation. |
| Taiwan |  | If the patient’s head is not connected to the body or obvious rigor mortis, etc. | If the patient’s is in DNR status |  |  |
| Thailand | After EMS arrive at the scene, the obvious sign of death was identify, the refraining of resuscitation was provided. | In case of palliative patient, which order of do not resuscitaiton, the EMS provider will refraining the resuscitation (Willing of death: the law of THAILAND) | Rescuer Safety: CPR should not be attempted in situations where performing resuscitation would expose the rescuer to serious injury or mortal danger. |  |  |
| Turkey | Prehospital healthcare professionals can refrain from resuscitation if obvious clinical signs of irreversible death are present |  |  |  |  |
| United Arab Emirates | All cases of non obvious death are resuscitated and transported |  |  |  |  |
| **Central America** |  |  |  |  |  |
| Costa Rica | presence of lividity in the body or signs of putrefaction | Prehospital healthcare professionals are legally obliged to adhere to a DNACPR |  |  |  |
| **Europa** |  |  |  |  |  |
| Austria | Injuries that are not compatible with survival, e.g. head separation or lividity, or rigor mortis. In the case of a priori-defined and taught signs of death (rigour mortis, decay, dead spots on the body or unsurvivable injuries), EMTs/paramedics can pronounce somebody dead even without resuscitative measures. The only person who can refrain from CPR initiation in case of absent clear signs of death would be a physician on screen (maybe even online with telemedicine in the future). |  |  |  |  |
| Belgium | If death is obvious (decapition, decomposition or extended charring), Ethical guidelines may inform doctors to refrain from resuscitation (in the absence of obvious dead or advance directives). | If there is known documented DNR paramedics can decide tot refrain from resuscitation |  |  |  |
| Bosnia and Herzegovina | Prehospital healthcare professionals can refrain from resuscitation if obvious clinical signs of irreversible death are present. |  |  |  |  |
| Croatia | Injuries incapable with life, certain signs of death (livor mortis, rigor mortis) |  |  |  |  |
| Czech Republic | Presence of certain signs of death. CPR can be refrained when signs of death, injuries incompatible with life are present. In the case of certain signs and symptoms of death. | A previously expressed wish, patient in paliative care. Terminal stage of incurable disease.  CPR can be refrained when advanced directive and/or terminal state (palliative care etc.) are present. | Danger to the life of the rescuer. Exhaustion of the rescuers, endangering the lives of the rescuers. It is not possible to ensure the safety of rescuers. |  |  |
| Denmark | EMTs / paramedics must initiate CPR unless the patient shows late signs of life extinct. Sure sighs of death. Special circumstances may dictate refraining from resuscitation, e.g., massive trauma, injuries incompatible with life etc. | The prehospital physician may decide to terminate resuscitation if additional information on e.g. time line, D.N.R documents etc are found. Terminal illness. Special circumstances may dictate refraining from resuscitation, e.g., terminal cancer, D.N.R documents discovered. |  |  |  |
| Finland | CPR is not initiated if (any of the following), applicated by all providers (BLS, ALS; etc.): - secondary sign of death | CPR is not initiated if (any of the following), applicated by all providers (BLS, ALS; etc.): - DNAR order exists - documented will by the patient is not to be resuscitated. In general, paramedics initiale resuscitation if there is no DNR. | CPR is not initiated if (any of the following), applicated by all providers (BLS, ALS; etc.): - no safe environment for personnel |  |  |
| France | Prehospital healthcare professionals can refrain from resuscitation if obvious clinical signs of irreversible death are present. | Prior written decision of the patient |  |  |  |
| Germany | Prehospital healthcare professionals can refrain from resuscitation if obvious clinical signs of irreversible death are present. | If a living will is available or if the underlying disease is hopeless. DNR order. |  |  |  |
| Greece | Resuscitation is terminated pre-hospitaly mainly/only for ... and incompatible with life injuries. The only cases EMS may refrain from resuscitation is when there is incompatible with life injuries, clinical evidence of prolonged death (unwitnessed & extremely cold/ cyanotic body/ livor mortis,/rigor mortis, etc). |  | The only cases EMS may refrain from resuscitation is when there is a clear risk/danger for the paramedic/doctor |  |  |
| Greenland | Prehospital healthcare professionals can refrain from resuscitation if obvious clinical signs of irreversible death are present |  |  |  |  |
| Hungary | Obvious signs of death present. Injuries incompatible with life. | End-stage of known terminal illness. Leagally correct DNAR order. |  |  |  |
| Iceland | Rigor/Livor mortis Decapitation or other obvious non-survivable injuries. | Prehospital healthcare professionals are legally obliged to adhere to a DNACPR |  |  |  |
| Ireland | As before, where signs of death are present | As before,... where there is DNACPR documentation available |  |  |  |
| Italy | In this case the practice is based on international guidelines and presence of evident signs of death. | In this case the practice is based on international guidelines and presence of terminal illness. |  |  |  |
| Kosovo |  |  |  |  | There is no prehospital refraining! |
| Luxembourg | All attending EMS members are advised to start CPR (unless obvious signs of death, like rigor, lesions incompatible with life,...) |  |  |  |  |
| Malta | Refraining from resuscitation only occurs when obvious irreversible signs of futility such as decapacitation, rigor mortis etc. This is determined by the consultant on site. This is determined by the consultant on site. Nurses start resuscitation on everyone. |  |  |  |  |
| Netherlands | When no doubt of death (wounds not compatible with life, rigor mortis, livor mortis etc.) If patient is obviously dead. | But sometimes depending on patient conditions, or the will of the patient (not to be resuscitated ). When there is a valid non-treatment statement. If patient has a DNR code |  |  |  |
| Norway | Obvious signs of death. The main "rule" is that rescucitation is initiated. Exceptions will be if there is clear signs of irreversible death (not witnessed, rigor mortis, cold without chance of accidential hypothermia and long time since patient witnessed alive). Unless the patient is obviously dead, there is no refraining from resuscitation without consulting a doctor. | DNR orders. The main "rule" is that rescucitation is initiated. Exceptions will be if there is at DNR order (could be in nursing homes). Unless a DNR order is known, there is no refraining from resuscitation without consulting a doctor. |  |  |  |
| Poland | Prehospital healthcare professionals can refrain from resuscitation if obvious clinical signs of irreversible death are present |  |  |  |  |
| Portugal | The medical doctor on-site may decide not to star CPR. Situations like the head separated form the body, decomposition, etc. | Prehospital healthcare professionals are legally obliged to adhere to a DNACPR |  |  |  |
| Russia | The legislation states generally that CPR should not be commenced in cases of "signs of biological death" and "consequences of acute injury incompatible with life". |  |  |  |  |
| Serbia | Prehospital healthcare professionals can refrain from resuscitation if obvious clinical signs of irreversible death are present |  |  |  |  |
| Slovenia | If cardiac arrest is recognized by the EMS dispatcher during an emergency phone-call (without obvious signs of death) the witnesses are encouraged to start CPR. | Prehospital healthcare professionals are legally obliged to adhere to a DNACPR |  |  |  |
| Spain | Prehospital healthcare professionals can refrain from resuscitation if obvious clinical signs of irreversible death are present | They are not initiated when the patient has orders not to resuscitate, when the patient is terminally ill. |  |  |  |
| Sweden | Ground ambulance are according to their SOPs allowed to refrain from starting cpr according to a few criteria (for example rigor mortis, head away from body). | Some patients with DNR at nursing homes can be terminated according to specific guidelines. |  |  |  |
| Switzerland | Prehospital healthcare professionals can refrain from resuscitation if obvious clinical signs of irreversible death are present | Prehospital healthcare professionals are legally obliged to adhere to a DNACPR |  |  |  |
| United Kingdom | Injuries incompatible with life Rigor mortis. In general, UK practice for refraining from commencing ALS is close to absent. The far most common approach is to start resuscitation and then decision-make, unless irrefutable signs of death e.g., livor mortis or clearly fatal injuries. Even then, ALS has known to be started. | Present DNACPR (do not resuscitate) order in place and available to view ADRT (Advanced directive) |  |  |  |
| **North America** |  |  |  |  |  |
| Canada | Commence CPR unless any of the following 1. obviously dead. | 2. the subject of a medical certificate of death, presented to the paramedic crew, in the form that is prescribed by the Vital Statistics Act (Ontario) and that appears on its face to be completed and signed in accordance with that Act; 3. without vital signs and the subject of an MOH Do Not Resuscitate (DNR) confirmation Form; 4. without vital signs and the subject of a Termination of Resuscitation (TOR) Order given by a Base Hospital Physician; or 5. without vital signs and the subject of a Withhold Resuscitation Order given by a Base Hospital Physician. |  |  |  |
| Mexico | Uncompatible with life injuries | In a few cases the patients' family members can choose not to begin resuscitation but show a legal document regarding the patient's will not to be resuscitated. | safety of the emergency personnel |  |  |
| United states (California) | Obvious death | POLST/DNR End-of-Life Options Act |  |  |  |
| United states (Colorado) | 1) An obvious death in the field is a standing order as long as the below criteria is met.  2) Determination of death in the field (without initiation of resuscitation) SHOULD include the following instances: a) Patient is unresponsive, pulseless, apneic, AND i) Decapitation, or ii) Decomposition, or iii) Rigor mortis or dependent lividity with warm air temperature, or iv) Any advanced directive or (1) Physician orders as specified on the Colorado Medical Orders for Scope of Treatment (MOST) form: “No  CPR. Do Not Resuscitate / DNR / Allow Natural Death”, present with the patient. v) Open cranium with exposed brain matter, or vi) Trauma incompatible with life, or vii) 3rd degree burns > 90% BSA (incineration) or viii) Multiple casualty situations where system resources are required for stabilization of viable patients. | If a patient has advanced directives they are followed with allowance for the EMS clinician to use clinical judgement in refraining from resuscitation. |  |  |  |
| United states (Florida) | At least 3 presumptive signs of death with 1 definitive sign of death | Cardiopulmonary resuscitation will be initiated on all patients who have suffered a cardiopulmonary arrest, except for those with a valid Do Not Resuscitate Order (DNRO) |  |  |  |
| United states (Illinois) | Obvious signs of death. Rigor, frozen..etc | Prehospital healthcare professionals are legally obliged to adhere to a DNACPR |  |  |  |
| United states (Intermountain) |  |  |  | Refraining from resuscitation can happen when there are signs of rigidity, evidence of other morbid conditions, trauma, co-morbid conditions. |  |
| United states (Massachusetts) | I. Exceptions to Initiation of Resuscitation Other than in overriding circumstances such as a large mass-casualty incident or a hazardous scene, the following are the only exceptions to initiating and maintaining resuscitative measures in the field: 3. Trauma inconsistent with survival a. Decapitation: severing of the vital structures of the head from the remainder of the patient’s body b. Transection of the torso: body is completely cut across below the shoulders and above the hips c. Evident complete destruction of brain or heart d. Incineration of the body e. Cardiac arrest (i.e. pulselessness) documented at first EMS evaluation when such condition is the result of significant blunt or penetrating trauma and the arrest is obviously and unequivocally due to such trauma, EXCEPT in the specific case of arrest due to penetrating chest trauma and short transport time to definitive care (in which circumstance, resuscitate and transport). 4. Body condition clearly indicating biological death. a. Complete decomposition or putrefaction: the skin surface (not only in isolated areas) is bloated or ruptured, with sloughing of soft tissue, and the odor of decaying flesh. b. Dependent lividity and/or rigor: when the patient’s body is appropriately examined, there is a clear demarcation of pooled blood within the body, and/or major joints (jaw, shoulders, elbows, hips, or knees) are immovable. | 1. Current, valid DNR, verified per this protocol. 2. Health care agent who is named in a health care proxy document for the patient requests no resuscitative efforts on behalf of the patient, but only if 1) he or she is on scene and 2) he or she has his/her health care proxy document in hand to show EMS. If there is any doubt about the health care agent’s authority, and none of the other exceptions to initiation of resuscitation are present, EMS is to resuscitate the patient. |  |  |  |
| United states (Michigan) | Dead on Scene inclusion criteria: Initiate or continue CPR for patient found to be in cardiac arrest UNLESS one or more of the following conditions exists: **A. Decomposition B. Rigor mortis (Caution: do not confuse with stiffness due to cold environment) C. Dependent lividity D. Decapitation E. Incinerated or frozen body F. Submersion greater than 1 hour documented by the licensed health care professional after arrival on scene. G. Gross dismemberment or obvious mortal wounds/conditions (injuries inconsistent with life - i.e., crushing injuries of the head and/or chest)** H. Unwitnessed arrest of traumatic origin, without organized electrical activity (must be asystolic or other rhythm with rate less than 40/min). J. In cases of mass casualty incidents, where the number of patients exceeds the providers and resources to care for them, any patient who is pulseless and apneic may be triaged as deceased. | I. Patient has a valid "Do Not Resuscitate" identification bracelet or order. |  |  |  |
| United states (Midwest) | No obvious signs of death, no signs of unsurvivable injury | No DNR |  |  |  |
| United states (Mississippi) | Prehospital healthcare professionals can refrain from resuscitation if obvious clinical signs of irreversible death are present | Prehospital healthcare professionals are legally obliged to adhere to a DNACPR |  |  |  |
| United states (Missouri) | Criteria to withhold resuscitation: Injuries incompatible with life. Lividity. Rigor Mortis. Tissue decomposition. Mass Casualty Incident where resources are not available to attempt or  continue prolonged resuscitation. | Valid Do Not Resuscitate Order is presented prior to initiation of  resuscitation | Criteria to withhold resuscitation: Resuscitation efforts pose a significant danger to the safety of providers. |  |  |
| United states (Nebraska) | Prehospital healthcare professionals can refrain from resuscitation if obvious clinical signs of irreversible death are present | Prehospital healthcare professionals are legally obliged to adhere to a DNACPR |  |  |  |
| United states (New England) | Prehospital healthcare professionals can refrain from resuscitation if obvious clinical signs of irreversible death are present | Prehospital healthcare professionals are legally obliged to adhere to a DNACPR |  |  |  |
| United states (New Jersey) | BLS may elect not to resuscitate if injuries incompatible with life; or rigor, lividity or decomposition present. | BLS may elect not to resuscitate if there is a valid out of hospital DNR order. |  |  |  |
| United states (Ohio) | no resuscitation if **obvious death (rigor)** or DNR status | no resuscitation if obvious death (rigor) or **DNR status** |  |  |  |
| United states (Oklahoma) | In the setting of obvious death - decapitation, decomposition, lividity, injury incompatible with life as specified in treatment protocols. | Do Not Resuscitate orders and/or Advanced Directives in other formats are honored when presented to on-scene EMS professionals. |  |  |  |
| United states (Oregon) | Resuscitation efforts may be withheld if: B. The patient is pulseless and apneic in a mass casualty incident or multiple patient scene where the resources of the system are required for the stabilization of living patients. C. The patient is decapitated. D. The patient has rigor mortis in a warm environment. E. The patient is in the stages of decomposition. F. The patient has skin discoloration in dependent body parts (dependent lividity). | Resuscitation efforts may be withheld if: A. The patient has a valid, signed "DNR” order. POLST registry # 888-476-5787 |  |  |  |
| United states (Tennessee) | Prehospital healthcare professionals can refrain from resuscitation if obvious clinical signs of irreversible death are present | Prehospital healthcare professionals are legally obliged to adhere to a DNACPR |  |  |  |
| United states (Texas) | Withholding resuscitation is done by protocl for obvious signs of death. | EMS Providers are NOT required to initiate resuscitation measures if the patient is apneic and pulseless, AND a cardiac rhythm strip shows asystole AND a Valid DNR. We begin resuscitation in the presence of cardiac arrest WITHOUT: Do Not Resuscitate Orders |  |  |  |
| United states (Utah-san Juan) | Automatic criteria for the initiation of resuscitation based on time down, physiologic parameters (rigor, lividity, asystole in trauma) | Prehospital healthcare professionals are legally obliged to adhere to a DNACPR |  |  |  |
| United states (Virginia) | If **obvious signs of death** or DNR present, no resuscitation will be attempted. Definitive" **signs of death (rigor, lividity, injuries incompatible with life)** or a valid "Do Not Resuscitate" order. | If obvious signs of death or **DNR present**, no resuscitation will be attempted. Definitive" signs of death (rigor, lividity, injuries incompatible with life) or **a valid "Do Not Resuscitate" order.** |  |  |  |
| United states (Washington) | No lividity or decompostion or injury not compatible with life.  INITIATION AND TERMINATION OF RESUSCITATION 1. If any of the following conditions are present, do not initiate CPR:  Cardiac arrest in the field due to blunt force trauma. Decapitation, total incineration, or obviously nonsurvivable injuries (i.e., extruded brain material). Decomposition, patient is cold in a warm environment, dependent lividity, or rigor mortis. Underwater submersion for 2 or more hours (consider extending 2-hour time if water temperature near freezing). Mass casualty situation where triage principles preclude CPR from being initiated on every victim. Discuss with medical control whenever possible after 15 minutes of ALS resuscitation for advice on treatment, termination, or transport. | Pt does not have DNR or POLST.  Patient has a terminal illness and next of kin requests not to perform CPR.  Valid advanced directive indicating no resuscitation. |  |  |  |
| United states (Wisconsin) | Prehospital healthcare professionals can refrain from resuscitation if obvious clinical signs of irreversible death are present | Prehospital healthcare professionals are legally obliged to adhere to a DNACPR |  |  |  |
| **Oceania** |  |  |  |  |  |
| Australia | In all cases of apparent cardiac arrest, CPR should be commenced unless:  2. There are obvious signs of death (e.g. decomposition / putrefaction / hypostasis / dependent lividity / rigor mortis) 3. Injuries are observed that are incompatible with life (e.g. decapitation / cranial & cerebral destruction / massive injury / incineration / foetal maceration) 4. Circumstances suggest that resuscitation may be futile (unless there has been cold water immersion): a. unwitnessed cardiac arrest with no bystander CPR, for greater than 15 minutes b. evidence of terminal illness or medical condition c. traumatic cardiac arrest with presenting ECG of asystole or slow, wide complex PEA 5. Multiple patients in accordance with triage principles | 6. A lawful direction to withhold resuscitation has been provided (or in a good faith assessment, highly likely to exist) unless inconsistent with good medical practice A lawful direction may include one of the following: a. a valid advanced health directive expressing the patient's wish for resuscitation to be withheld or withdrawn b. a verbal decision conveyed by the patient's health attorney c. verbal information from a medical practitioner present | 1. Performing CPR would endanger the lives or safety of the paramedics, bystanders or other first responders. |  |  |
| New Zealand | Resuscitation should start unless there is a clear reason not to. Clear reasons for not starting resuscitation include: Signs of rigor mortis or post-mortem lividity. | This relates to advanced directives, advanced care plans, DNAR requests. Resuscitation should start unless there is a clear reason not to. Clear reasons for not starting resuscitation include: A clear advance directive not to receive resuscitation for cardiac arrest. |  |  |  |
| **South America** |  |  |  |  |  |
| Brazil | In cases where the caller reports obvious signs of death such as a charred body, a body in an advanced state of decomposition, or the presence of cadaveric fauna, the primary regulatory physician may instruct contacting the military police and medical examiner's office. Signs of obvious death (rigor mortis, livor mortis, decapitation, crushed skull with loss of brain mass and absence of central pulse, charring, body segmentation, or clear signs of decomposition). | Metastatic cancer or other terminal pathology (with no possibility of treatment, described in a medical report or informed by a responsible family member). • Cardiac arrest in terminal stage patients with severe and incurable illnesses with family consensus not to perform resuscitation. |  | Signs of obvious death (rigor mortis, livor mortis, decapitation, crushed skull with loss of brain mass and absence of central pulse, charring, body segmentation, or clear signs of decomposition). • Metastatic cancer or other terminal pathology (with no possibility of treatment, described in a medical report or informed by a responsible family member). • Clinical immobility syndrome (dementia and/or severe malnutrition and/or bedridden and/or entirely dependent on a caregiver for daily activities). • Cardiac arrest in terminal stage patients with severe and incurable illnesses with family consensus not to perform resuscitation. • In cases where the caller reports obvious signs of death such as a charred body, a body in an advanced state of decomposition, or the presence of cadaveric fauna, the primary regulatory physician may instruct contacting the military police and medical examiner's office. It is important to determine the reliability of the information provided and the clarity of the data reported by the caller. |  |
| Chile | Prehospital healthcare professionals can legally refrain from resuscitation if obvious clinical signs of irreversible death are present | Prehospital healthcare professionals are legally obliged to adhere to a DNACPR |  |  |  |
| Peru |  |  |  | It is the discretion of the person assisting the case. |  |

**Supplementary file 8 – Refraining from resuscitation table**

| **Country** | **The instructions that determine the practice regarding refraining from resuscitation** | **The authority that can decide to refrain resuscitation** | **The practice regarding**  **refraining from resuscitation** | **DNACPR** | **Obvious signs**  **of death** |
| --- | --- | --- | --- | --- | --- |
| **Africa** |  |  |  |  |  |
| Kenya | Standardized protocols, Guidelines, Medical directives | Physician (specialist) on scene, Physician (specialist) via phone, Physician at a hospital, Paramedic, Emergency medical technician, Nurse | Patients with obvious signs of irreversible death,  Patients with confirmed advanced directives, Performing CPR would endanger the lives or safety of the EMS personnel | Yes | Livor Mortis, Rigor Mortis, Decomposition, Injuries incompatible with life,  Burned beyond recognition |
| Rwanda | None | N/A | No refraining from resuscitation in the prehospital setting – all patients are uniformly resuscitated without exception. | No | No |
| Sierra Leone | Standardized protocols  Rules  Guidelines | Physician (specialist) on scene  Physician on scene | Patients with obvious signs of irreversible death | No | Injuries incompatible with life |
| South Africa | Standardized protocols, Rules, Guidelines, Medical directives, Decision-making without the use of guidelines or protocols, On-site medical control | Physician (specialist) on scene, Physician (specialist) via phone, Physician on scene, Physician at a hospital, Paramedic, Emergency medical technician, Emergency Care Practitioner | Patients with obvious signs of irreversible death. | No | Livor Mortis, Rigor Mortis, Decomposition, Injuries incompatible with life,  Burned beyond recognition |
| Tunisia | Guidelines,  Medical directives,  On-site medical control | Physician (specialist) on scene,  Physician (specialist) via phone,  Physician on scene | Patients with obvious signs of irreversible death | No | Livor Mortis, Rigor Mortis, Decomposition, Injuries incompatible with life,  Burned beyond recognition |
| Uganda | Standardized protocols,  Guidelines,  On-site medical control | Paramedic,  Nurse,  Laymen | Patients with obvious signs of irreversible death,  Patients with confirmed advanced directives,  Performing CPR would endanger the lives or safety of the EMS personnel. | Yes | Livor Mortis, Rigor Mortis, Decomposition, Injuries incompatible with life,  Burned beyond recognition |
| **Asia** |  |  |  |  |  |
| China | Standardized protocols,  On-site medical control | Physician (specialist) on scene,  Physician on scene | Patients with obvious signs of irreversible death. | No | Livor Mortis, Rigor Mortis, Decomposition, Injuries incompatible with life,  Burned beyond recognition |
| India | None | Physician on-scene (in some parts of the country),  Emergency medical technician (in some parts of the region) | Performing CPR would endanger the lives or safety of the EMS personnel. | No | No |
| Japan | Standardized protocols, Medical directives, Online medical control | Physician (specialist) via phone, Physician at a hospital, Paramedic, Emergency medical technician | Patients with obvious signs of irreversible death. | No | Livor Mortis, Rigor Mortis, Decomposition, Injuries incompatible with life,  Burned beyond recognition |
| Jordan | Decision-making without the use of guidelines or protocols (the medical direction is still limited) | Paramedic,  Police- or firemen | Patients with obvious signs of irreversible death. | No | Livor Mortis, Rigor Mortis, Decomposition, Injuries incompatible with life,  Burned beyond recognition |
| Malaysia | Guidelines,  Medical directives,  Online medical control,  On-site medical control | Physician (specialist) on scene,  Physician (specialist) via phone,  Paramedic | Patients with obvious signs of irreversible death,  Patients with confirmed advanced directives | Yes | Rigor Mortis, Decomposition, Injuries incompatible with life,  Burned beyond recognition |
| Philippines | Standardized protocols, Rules, Guidelines, Medical directives, Online medical control, On-site medical control | Physician (specialist) on scene,  Physician (specialist) via phone,  Physician on scene,  Paramedic,  Emergency medical technician,  Police- or firemen,  Laymen | Patients with obvious signs of irreversible death,  Patients with confirmed advanced directives | Yes, but not legally obliged to adhere | Livor Mortis, Rigor Mortis, Decomposition, Injuries incompatible with life,  Burned beyond recognition |
| Qatar | Standardized protocols,  Guidelines | Paramedic,  Nurse | Patients with obvious signs of irreversible death,  Patients with confirmed advanced directives. | Yes | Livor Mortis, Rigor Mortis, Decomposition, Injuries incompatible with life,  Fetal maceration, Evisceration of major organs, Incineration |
| Saudi Arabia | Standardized protocols, Rules, Guidelines, Medical directives, Online medical control, Decision-making without the use of guidelines or protocols, On-site medical control | Physician (specialist) on scene,  Physician (specialist) via phone, Physician at a hospital, Paramedic, Emergency medical technician | Patients with obvious signs of irreversible death,  Patients with confirmed advanced directives | Yes | Livor Mortis, Rigor Mortis, Decomposition, Injuries incompatible with life,  Burned beyond recognition |
| Singapore | Standardized protocols, Guidelines, Online medical control | Physician (specialist) via phone,  Paramedic | Patients with obvious signs of irreversible death,  Patients with confirmed advanced directives | Yes | Livor Mortis, Rigor Mortis, Decomposition, Injuries incompatible with life,  Burned beyond recognition |
| South Korea | Guidelines | Physician (specialist) on scene,  Physician (specialist) via phone,  Physician on scene | Patients with obvious signs of irreversible death,  Patients with confirmed advanced directives,  Performing CPR would endanger the lives or safety of the EMS personnel. | Yes but not legally obliged to adhere | Rigor Mortis, Decomposition, Injuries incompatible with life |
| Sri Lanka | Standardized protocols,  Medical directives,  Online medical control,  Decision-making without the use of  guidelines or protocols | Physician at a hospital | No refraining from resuscitation in the prehospital setting– all patients are uniformly resuscitated without exception | No | No |
| Taiwan | Standardized protocols, Rules, Guidelines | Paramedic, Emergency medical technician | Patients with obvious signs of irreversible death,  Patients with confirmed advanced directives | Yes | Livor Mortis, Rigor Mortis, Decomposition, Injuries incompatible with life,  Burned beyond recognition |
| Thailand | Standardized protocols, Guidelines, Online medical control | Physician (specialist) on scene, Physician (specialist) via phone, Physician on scene, Physician at a hospital | Patients with obvious signs of irreversible death,  Patients with confirmed advanced directives, Performing CPR would endanger the lives or safety of the EMS personnel | Yes | Livor Mortis, Rigor Mortis, Decomposition, Injuries incompatible with life,  Burned beyond recognition |
| Turkey | Standardized protocols,  Guidelines | Physician on scene | Patients with obvious signs of irreversible death | No | Livor Mortis, Rigor Mortis, Decomposition, Injuries incompatible with life |
| United Arab Emirates | Rules | Paramedic | Patients with obvious signs of irreversible death | No | Rigor Mortis, Decomposition, Injuries incompatible with life,  Burned beyond recognition |
| **Central America** |  |  |  |  |  |
| Costa Rica | Decision-making without the use of guidelines or protocols | Physician (specialist) via phone | Patients with obvious signs of irreversible death,  Patients with confirmed advanced directives | Yes, but not legally obliged to adhere | Livor Mortis, Decomposition, Injuries incompatible with life,  Burned beyond recognition |
| **Europa** |  |  |  |  |  |
| Austria | Standardized protocols,  Rules,  Guidelines,  Medical directives,  On-site medical control, Decision-making without the use of guidelines or protocols | Physician (specialist) on scene,  Physician on scene,  Physician at a hospital,  Paramedic,  Emergency medical technician | Patients with obvious signs of irreversible death | No | Livor Mortis, Rigor Mortis, Decomposition, Injuries incompatible with life,  Burned beyond recognition |
| Belgium | Rules,  Guidelines, Decision-making without the use of guidelines or protocols | Physician (specialist) on scene,  Physician (specialist) via phone,  Physician on scene,  Physician at a hospital,  Paramedic,  Nurse | Patients with obvious signs of irreversible death,  Patients with confirmed advanced directives. | Yes | Livor Mortis, Rigor Mortis, Decomposition, Injuries incompatible with life,  Burned beyond recognition |
| Bosnia and Herzegovina | Standardized protocols,  rules,  Guidelines | Physician (specialist) on scene | Patients with obvious signs of irreversible death | No | Decomposition,  Injuries incompatible with life, Burned beyond recognition |
| Croatia | Guidelines,  On-site medical control | Physician (specialist) on scene,  Physician on scene | Patients with obvious signs of irreversible death | No | Livor Mortis, Rigor Mortis, Decomposition, Injuries incompatible with life,  Burned beyond recognition |
| Czech Republic | Standardized protocols, Rules, Guidelines, Medical directives, Online medical control, Decision-making without the use of guidelines or protocols, On-site medical control | Physician (specialist) on scene,  Physician (specialist) via phone,  Physician on scene, Physician at a hospital,  Paramedic,  Nurse | Patients with obvious signs of irreversible death,  Patients with confirmed advanced directives, Performing CPR would endanger the lives or safety of the EMS personnel | Yes | Livor Mortis, Rigor Mortis, Decomposition, Injuries incompatible with life,  Burned beyond recognition, Tonnelli sign |
| Denmark | Standardized protocols, Rules, Guidelines, Medical directives, Decision-making without the use of guidelines or protocols, On-site medical control | Physician (specialist) on scene, Physician (specialist) via phone,  Physician at a hospital, Paramedic | Patients with obvious signs of irreversible death,  Patients with confirmed advanced directives | Yes | Livor Mortis, Rigor Mortis, Decomposition, Injuries incompatible with life,  Burned beyond recognition |
| Finland | Standardized protocols, Rules, Guidelines, Medical directives, Online medical control On-site medical control | Physician (specialist) on scene, Physician (specialist) via phone, Physician on scene, Physician at a hospital, Paramedic | Patients with obvious signs of irreversible death,  Patients with confirmed advanced directives, Performing CPR would endanger the lives or safety of the EMS personnel | Yes | Livor Mortis, Rigor Mortis, Decomposition, Injuries incompatible with life,  Burned beyond recognition |
| France | On-site medical control | Physician (specialist) on scene, Physician (specialist) via phone, Physician on scene | Patients with obvious signs of irreversible death,  Patients with confirmed advanced directives | Yes | Livor Mortis, Rigor Mortis, Decomposition, Injuries incompatible with life,  Burned beyond recognition |
| Germany | Guidelines, Medical directives, Decision-making without the use of guidelines or protocols, On-site medical control | Physician (specialist) on scene, Physician on scene | Patients with obvious signs of irreversible death,  Patients with confirmed advanced directives | Yes | Livor Mortis, Rigor Mortis, Decomposition, Injuries incompatible with life,  Burned beyond recognition |
| Greece | Rules, Guidelines, On-site medical control | Physician (specialist) on scene, Physician (specialist) via phone, Physician on scene, Physician at a hospital, Paramedic | Patients with obvious signs of irreversible death,  Performing CPR would endanger the lives or safety of the EMS personnel | No | Livor Mortis, Rigor Mortis, Decomposition, Injuries incompatible with life,  Burned beyond recognition |
| Greenland | Online medical control | Physician (specialist) via phone, Physician at a hospital,  Nurse | Patients with obvious signs of irreversible death. | No | Livor Mortis, Rigor Mortis, Decomposition, Injuries incompatible with life,  Burned beyond recognition |
| Hungary | Rules, Guidelines, Decision-making without the use of guidelines or protocols | Physician (specialist) on scene, Physician (specialist) via phone, Physician on scene, Physician at a hospital, Paramedic | Patients with obvious signs of irreversible death,  Patients with confirmed advanced directives | Yes | Livor Mortis, Rigor Mortis, Decomposition, Injuries incompatible with life,  Burned beyond recognition |
| Iceland | Standardized protocols, Rules, Guidelines, Medical directives, Online medical control, On-site medical control | Physician (specialist) on scene, Physician (specialist) via phone, Physician on scene, Physician at a hospital, Paramedic, Emergency medical technician | Patients with obvious signs of irreversible death,  Patients with confirmed advanced directives | Yes | Livor Mortis, Rigor Mortis, Decomposition, Injuries incompatible with life,  Burned beyond recognition |
| Ireland | Standardized protocols,  Guideline, Online medical control | Physician (specialist) on scene,  Physician (specialist) via phone,  Paramedic,  Emergency medical technician,  Nurse,  Police- or firemen,  Laymen | Patients with obvious signs of irreversible death,  Patients with confirmed advanced directives | Yes but not legally obliged to adhere | Livor Mortis, Rigor Mortis, Decomposition, Injuries incompatible with life,  Burned beyond recognition |
| Italy | Standardized protocols, Guidelines | Physician (specialist) on scene, Physician on scene, Physician at a hospital, Nurse | Patients with obvious signs of irreversible death,  Patients with confirmed advanced directives | Yes | Livor Mortis, Rigor Mortis, Decomposition, Injuries incompatible with life,  Burned beyond recognition |
| Kosovo | None | Physician on scene | No refraining from resuscitation in the prehospital setting – all patients are uniformly resuscitated without exception. | No | No |
| Luxembourg | Medical directives, On-site medical control | Physician (specialist) on scene, Physician on scene | Patients with obvious signs of irreversible death. | No | Livor Mortis, Rigor Mortis, Decomposition, Injuries incompatible with life,  Burned beyond recognition |
| Malta | Decision-making without the use of guidelines or protocols | Physician (specialist) on scene, Physician on scene, Physician at a hospital | Patients with obvious signs of irreversible death | No | Livor Mortis, Decomposition, Injuries incompatible with life |
| Nederland | Standardized protocols, Rules, Guidelines, Medical directives, Online medical control, Decision-making without the use of guidelines or protocols, On-site medical control | Physician (specialist) on scene, Physician (specialist) via phone, Physician on scene, Physician at a hospital, Paramedic, Emergency medical technician, Nurse,  Police- or firemen,  Laymen | Patients with obvious signs of irreversible death,  Patients with confirmed advanced directives | Yes | Livor Mortis, Rigor Mortis, Decomposition, Injuries incompatible with life,  Burned beyond recognition |
| Norway | Standardized protocols, Rules, Guidelines, Medical directives, Online medical control, Decision-making without the use of guidelines or protocols, On-site medical control | Physician (specialist) on scene, Physician (specialist) via phone, Physician on scene, Physician at a hospital, Paramedic | Patients with obvious signs of irreversible death,  Patients with confirmed advanced directives | Yes | Livor Mortis, Rigor Mortis, Decomposition, Injuries incompatible with life,  Burned beyond recognition |
| Poland | Standardized protocols, Guidelines | Physician (specialist) on scene, Physician on scene | Patients with obvious signs of irreversible death. | No | Livor Mortis, Rigor Mortis, Decomposition, Injuries incompatible with life |
| Portugal | On-site medical control | Physician on scene | Patients with obvious signs of irreversible death,  Patients with confirmed advanced directives. | Yes, but not legally obliged to adhere | Livor Mortis, Rigor Mortis, Decomposition, Injuries incompatible with life |
| Russia | The Federal law | Physician (specialist) on scene,  Physician on scene, Feldsher (mid-level EMS provider) | Patients with obvious signs of irreversible death | No | Livor Mortis, Rigor Mortis, Decomposition, Injuries incompatible with life, Burned beyond recognition,  Signs of biological death |
| Serbia | Guidelines, Medical directives, Decision-making without the use of guidelines or protocols | Physician (specialist) on scene, Physician on scene | Patients with obvious signs of irreversible death | No | Livor Mortis, Rigor Mortis, Decomposition, Injuries incompatible with life,  Burned beyond recognition |
| Slovenia | Guidelines, Medical directives, Decision-making without the use of guidelines or protocols | Physician (specialist) on scene, Physician on scene | Patients with obvious signs of irreversible death | No | Livor Mortis, Rigor Mortis, Decomposition, Injuries incompatible with life,  Burned beyond recognition |
| Spain | Guidelines, Decision-making without the use of guidelines or protocols, On-site medical control | Physician (specialist) on scene, Physician (specialist) via phone, Physician on scene,  Physician at a hospital | Patients with obvious signs of irreversible death,  Patients with confirmed advanced directives | Yes | Livor Mortis, Rigor Mortis, Decomposition, Injuries incompatible with life,  Burned beyond recognition |
| Sweden | Standardized protocols, Guidelines, Medical directives, Online medical control, Decision-making without the use of guidelines or protocols, On-site medical control | Physician (specialist) on scene, Physician (specialist) via phone, Physician on scene, Physician at a hospital,  Nurse, Police- or firemen | Patients with obvious signs of irreversible death,  Patients with confirmed advanced directives | Yes | Livor Mortis, Rigor Mortis, Decomposition, Injuries incompatible with life,  Burned beyond recognition |
| Switzerland | Standardized protocols, Guidelines, Decision-making without the use of guidelines or protocols, On-site medical control | Physician (specialist) on scene, Physician on scene, Paramedic | Patients with obvious signs of irreversible death,  Patients with confirmed advanced directives | Yes, but not legally obliged to adhere | Livor Mortis, Rigor Mortis, Decomposition, Injuries incompatible with life,  Burned beyond recognition |
| United Kingdom | Standardized protocols, Medical directives, Online medical control, Decision-making without the use of guidelines or protocols, On-site medical control | Physician (specialist) on scene,  Physician (specialist) via phone, Physician on scene, Paramedic,  Nurse, Police- or firemen | Patients with obvious signs of irreversible death,  Patients with confirmed advanced directives | Yes | Livor Mortis, Rigor Mortis, Decomposition, Injuries incompatible with life,  Burned beyond recognition |
| **North America** |  |  |  |  |  |
| Canada | Standardized protocols, Medical directives, Online medical control | Physician (specialist) via phone, Physician at a hospital,  Paramedic | Patients with obvious signs of irreversible death,  Patients with confirmed advanced directives | Yes | Livor Mortis, Rigor Mortis, Decomposition, Injuries incompatible with life,  Burned beyond recognition |
| Mexico | Rules,  Guidelines | Physician (specialist) via phone,  Paramedic,  Emergency medical technician,  Police- or firemen,  Laymen | Patients with obvious signs of irreversible death,  Patients with confirmed advanced directives | Yes | Livor Mortis, Rigor Mortis, Decomposition, Injuries incompatible with life,  Burned beyond recognition |
| United States - Midwest | Standardized protocols, Guidelines, Online medical control | Physician (specialist) on the scene, Physician (specialist) via phone, Physician on scene, Physician at a hospital (in some parts of the region), Paramedic, Emergency medical technician (in some parts of the region) | Patients with obvious signs of irreversible death,  Patients with confirmed advanced directives | Yes | Livor Mortis, Rigor Mortis, Decomposition, Injuries incompatible with life,  Burned beyond recognition |
| United States - Northeast | Standardized protocols, Rule, Online medical control | Physician (specialist) on the scene, Physician (specialist) via phone, Physician on scene, Physician at a hospital, Paramedic, Emergency medical technician | Patients with obvious signs of irreversible death,  Patients with confirmed advanced directives | Yes | Livor Mortis, Rigor Mortis, Decomposition, Injuries incompatible with life,  Burned beyond recognition |
| United States - South | Standardized protocols, Guidelines, Medical directives, Online medical directives, Decision-making without the use of guidelines or protocols (in some parts of the region) | Physician (specialist) on scene, Physician (specialist) via phone, Physician on scene, Physician at a hospital, Paramedic, Emergency medical technician (in some parts of the region), Nurse (in some parts of the region), Police- or firemen (in some parts of the region) | Patients with obvious signs of irreversible death,  Patients with confirmed advanced directives | Yes | Livor Mortis, Rigor Mortis, Decomposition, Injuries incompatible with life, Burned beyond recognition, Frozen body,  Submersion greater than 1 hour documented by the licensed health care professional after arrival on scene |
| United States - West | Standardized protocols | Physician (specialist) on the scene, Physician (specialist) via phone, Physician on scene, Physician at a hospital, Paramedic, Emergency medical technician (in some parts of the region), Nurse (in some parts of the region) | Patients with obvious signs of irreversible death,  Patients with confirmed advanced directives | Yes | Livor Mortis (in some parts of the region), Rigor Mortis, Decomposition, Injuries incompatible with life,  Burned beyond recognition |
| **Oceania** |  |  |  |  |  |
| Australia | Standardized protocols, Rules, Guidelines, Medical directives | Physician (specialist) on scene, Physician (specialist) via phone, Physician on scene, Paramedic, Nurse | Patients with obvious signs of irreversible death,  Patients with confirmed advanced directives, Performing CPR would endanger the lives or safety of the EMS personnel | Yes | Livor Mortis, Rigor Mortis, Decomposition, Injuries incompatible with life,  Burned beyond recognition |
| New Zealand | Guidelines, Medical directives | Physician (specialist) via phone, Paramedic, Emergency medical technician | Patients with obvious signs of irreversible death,  Patients with confirmed advanced directives | Yes | Livor Mortis, Rigor Mortis, Decomposition, Injuries incompatible with life,  Burned beyond recognition |
| **South America** |  |  |  |  |  |
| Brazil | Standardized protocols | Physician (specialist) on scene,  Physician (specialist) via phone,  Physician on scene | Discretion of the care provider, Patients with obvious signs of irreversible death, Patients with confirmed advanced directives | Yes | Livor Mortis, Rigor Mortis, Decomposition, Injuries incompatible with life |
| Chile | Online medical control,  Decision-making without the use of guidelines or protocols,  On-site medical control | Physician (specialist) on scene,  Physician (specialist) via phone,  Physician on scene,  Physician at a hospital | Patients with obvious signs of irreversible death,  Patients with confirmed advanced directives | Yes | Livor Mortis, Rigor Mortis, Decomposition, Injuries incompatible with life,  Burned beyond recognition |
| Peru | None | The one with the highest rank at the time of assistance. | Discretion of the care provider | No | No |

If at least one participant from a country provided an answer, it is included in the table.
