# Supplementary material for: Prehospital cardiac arrest resuscitation practices differ around the globe
Source: Resusc Plus. 2025 Jun 24;25:101017. doi: 10.1016/j.resplu.2025.101017 (PMC12329125; doi:10.1016/j.resplu.2025.101017)
Supplement: Supplementary Data 2 [file mmc2.docx]

# E-mail complete

*Figure filtered on "Nyt figurfilter 2"*

| **Email** | **Please provide full name, email, and affiliation.**  **(Your answers will be anonymized in the article).** |
| --- | --- |
| sabine.lemoine@pompiersparis.fr | Sabine Lemoine, MD  Fire Brigade of Paris  Emergency department  Paris France |
| d.jaeger@chru-nancy.fr | Dr Déborah JAEGER, MCU-PH  Cheffe de Service adjointe  SAMU-SMUR-Urgences- CHRU de Nancy  UMR_S 1116 : Défaillance Cardiovasculaire Aiguë et Chronique  Tel Bureau : 03.83.85.17.63  Tel Secrétariat (Mme Laine) : 03.83.85.14.96 |
| dilrukir84@gmail.com | Rathnayake Mudiyanselage Dilruk Indika Rathnayake  dilrukir84@gmail.com  Emergency Physician (Act)  Sri Lanka Army |
| lampkrex@hku.hk | Dr. Rex Pui Kin Lam  Clinical Associate Professor of Practice  Department of Emergency Medicine, School of Clinical Medicine, Li Ka Shing Faculty of Medicine, The University of Hong Kong |
| agim.krasniqi@swisstph.ch | Agim Krasniqi  email: agim.krasniqi@swisstph.ch  Kosova Albanian |
| turhans112@gmail.com | Dr. Turhan SOFUOGLU  Emergency Disaster Ambulance Physicians Association (AAHD) |
| dusabimanasimon1994@gmail.com | Dusabimana Simon |
| unisakanu1@gmail.com | Unisa Kanu, a surgical community health officer working with partners in health PIH in Sierra Leone as emergency surgical officer in maternity ward. |
| russellbonnici@hotmail.co.uk | Russell Bonnici Farrugia, MD (Melit.), MRCEM (UK)  russellbonnici@hotmail.co.uk  Emergency Department,  Mater Dei Hospital,  Malta |
| dzsupiar@gmail.com | Mihaela Budimski Soldat  dzsupiar@gmail.com  Emergency medicine specialist |
| gemi@peqqik.gl | George Michagin  e-mail: gemi@peqqik.gl  affiliation: Droning Ingrids hospital, Nuuk, Greenland |
| dam.morais@gmail.com | DANIELA APARECIDA MORAIS  dam.morais@gmail.com  Serviço de Atendimento Móvel de Urgência de Belo Horizonte (SAMU-BH) - Minas Gerais, Brazil (Mobile Emergency Care Service in Belo Horizonte - Minas Gerais, Brazil) |
| Naseef.abdullah@westerncape.gov.za | Naseef Abdullah  naseef.abdullah@westerncape.gov.za  Western Cape Department of Health Emergency Medical Service |
| meyerjo@cput.ac.za | John Thomas Meyer  meyerjo@cput.ac.za  Cape Peninsula University of Technology: Department of Emergency Medical Sciences  Cape Town South Africa |
| galinier@hamad.qa | Prof Guillaume Alinier  Director of Research  Hamad Medical Corporation Ambulance Service  Doha, Qatar  GAlinier@hamad.qa  Visiting Professor, University of Hertfordshire, Hatfield, and Northumbria University, Newcastle upon Tyne, UK  Adjunct Professor, Weill Cornell Medicine-Qatar, Doha, Qatar |
| arendt75@hanyang.ac.kr | Ki-Ok Ahn, MD, PhD  Department of Emergency Medicine, Myongji Hospital  Hanyang University College of Medicine |
| drbaljitsingh@gmail.com | Baljit Singh  drbaljitsingh@gmail.com  Professor Emeritus, Tantia University, Sri Ganganagar (Rajasthan), India. |
| tajnica.hdhm@hotmail.com | Anđela Simić, tajnica.hdhm@hotmail.com, Institute of Emergency Medicine Varazdin County, Croatia |
| strnad.matej78@gmail.com | Matej Strnad, MD, PhD  University medical centre Maribor, Emergency department, Ljubljanska ul. 5, 2000 Maribor, Slovenia  Community heathcare centre, Emergency medical services, Prehospital unit, C. proletarskih brigad 21, 2000 Maribor, Slovenia  University of Maribor, Medical faculty, taborska ul. 8, 2000 Maribor, Slovenia  strnad.matej78@gmail.com  matej.strnad@um.si |
| marko.noc@mf.uni-lj.si | Marko Noc  Center for intensive internal medicine  University medical center  Ljubljana-Slovenia |
| vesnick@gmail.com | Vesna Borovnik Lesjak  vesnick@gmail.com  Emergency medical services, Prehospital unit, Community Health Center Maribor, Slovenia  Emergency medical dispatch services University Medical Center Ljubljana, Slovenia |
| ppariw@kku.ac.th | pariwat phungoen  ppariw@kku.ac.th  Department of Emergency Medicine, Faculty of Medicine, Khon Kaen University, Khon Kaen, Thailand |
| andrej.markota@ukc-mb.si | Andrej Markota  andrej.markota@ukc-mb.si  Medical Intensive Care Unit  University Medical Centre Maribor  Ljubljanska ulica 5  2000 Maribor  Slovenia |
| siobhan.masterson@hse.ie | Siobhan Masterson  General Manager, HSE National Ambulance Service  Adjunct Senior Lecturer, University College Cork  Adjunct Lecturer, University of Galway |
| arthurck@hku.hk | Prof. Arthur Chi-Kin Cheung  arthurck@hku.hk  Department of Emergency Medicine, School of Clinical Medicine, LKS Faculty of Medicine, The University of Hong Kong, Hong Kong, China |
| korakot@kku.ac.th | Assoc. Prof. Korakot Apiratwarakul  E-mail: korakot@kku.ac.th  Department of Emergency Medicine, Faculty of Medicine, Khon Kaen University, Thailand |
| Salwajeeh2021@gmail.com | Sultan Ali Alwajeeh.  Email: salwajeeh2021@gmail.com  Ministry of Health |
| mtalwidyan@just.edu.jo | Mahmoud T. Alwidyan, PhD  Associate professor of paramedicine  Department of Allied Medical Sciences  Jordan University of Science and Technology, Jordan |
| jmfraga@emergencias.com.mx | Juan-Manuel Fraga-Sastrias MD, DHlthSc  Director  Asesores en Emergencias  jmfraga@emergencias.com.mx |
| convocar.pauline@gmail.com | Pauline Convocar  convocar.pauline@gmail.com  Asian Society for Emergency Medicine  Manila Doctors Hospital |
| papousekr@zzsjck.cz | MUDr René Papoušek  Zdravotnická záchranná služba Jihočeského kraje p.o. |
| peter.hilbert-Carius@etern.drf-luftrettung.de | Peter Hilbert-Carius, PhD  DRF Station Halle  Lilienthalstraße 1  06188 Landsberg  Germany |
| jossewanyana@gmail.com | Josephine Ssewanyana  jossewanyana@gmail.com |
| marcus.rudolph@drf-luftrettung.de | Dr. med. Marcus Rudolph EDIC FFTACC  DRF Stiftung Luftrettung gAG  Rita Maiburg Str. 2  70794 Filderstadt |
| erwin.snijders@uza.be | Erwin Snijders  erwin.snijders@uza.be  MD, emergency physician, University hospital of Antwerp |
| Emsqualitymhsz@gmail.com | Muhammad sultan zaher  Paramedic and quality designee  Working at Kau university hospital  Emsqualitymhsz@gmail.com |
| Lkalsulimani@kau.edu.sa | Loui K Alsulimani  Lkalsulimani@kau.edu.sa  Disaster medicine section, department of emergency medicine, King Abdualaziz University, Jeddah, Saudi Arabia |
| hajriz.a@hotmail.com | Hajriz Alihodžić.Emergency Medical Service, Public Institution Health Centre ‘Dr. Mustafa Šehović’ and Faculty of Medicine, University of Tuzla, 75000 Tuzla, Bosnia and Herzegovina |
| neuron.shook_0h@icloud.com | Tem |
| popelas@zzsjmk.cz | +- |
| jacqueline.eleonora@gmail.com | Jacqueline Eleonora Ek, M.D, MRCEM  Emergency Department, Mater Dei Hospital, Malta |
| popelas@zzsjmk.cz | Stanislav Popela, MD, PhD.  stanislav.popela@seznam.cz  EMS South Moravian region - Czech Republic  Emergency physician  https://orcid.org/0000-0003-2577-9244 |
| drcairolb@gmail.com | Andrés Cairol, acairol@unibe.ac.cr, Costa Rica Pre-hospital Research Team |
| jana.vidunova@zzspk.cz | Jana Kruba Vidunová  jana.vidunova@zzspk.cz  EMS of Pilsen Region, Czech Republic |
| david.peran@zzskvk.cz | Dr. David Peran, Ph.D., FERC  david.peran@zzskvk.cz  Emergency Medical Services of Karlovy Vary Region, Czech Republic  Department of Anaesthesia and Intensive Care Medicine, Charles University, Third Faculty of Medicine and FNKV University Hospital in Prague, Czech Republic |
| vicentes@landspitali.is | Vicente Sánchez-Brunete Ingelmo, MD  EMS Medical Director - Yfirlæknir bráðaþjónustu utan sjúkrahúsa  Department of Emergency Medicine  Landspitali University Hospital  108 Reykjavík, Iceland  vicentes@landspitali.is  tel:+354 5431000, mobile:+354 6596681 |
| hjaltimb@landspitali.is | Hjalti Már Björnsson MD FACEP FRCEM  Training program director  Department of Emergency Medicine  Landspitali - The National University Hospital of Iceland  Associate Professor of Emergency Medicine  University of Iceland |
| andjela.simic.005@gmail.com | Anđela Simić  andjela.simic.005@gmail.com  Educational Institute of Emergency Medicine of Varazdin County |
| pascal.stammet@ext.uni.lu | Pascal STAMMET  pascal.stammet@ext.uni.lu; stammet.pascal@chl.lu  Centre Hospitalier de Luxembourg, Luxembourg  University of Luxembourg, Esch-sur-Alzette, Luxembourg |
| raffo.escalante@gmail.com | Raffo Escalante-Kanashiro  raffo.escalante@gmail.com  rescalante@insn.gob.pe  Instituto Nacional de Salud del Niño  Universidad Peruana de Ciencias Aplicadas |
| stvncraw@gmail.com | Steven John Crawford  University of Doha for Science and Technology |
| olynmed@yahoo.gr | Olympia Nikolaidou  olynmed@yahoo.gr  National Center for Emergency Care (EMS-Thessaloniki, Greece) |
| Bernd.wallner@i-med.ac.at | Bernd Wallner |
| bkarageorgos@hotmail.com | Vlasios Karageorgos, bkarageorgos@hotmail.com, Cardiopulmonary Resuscitation Laboratory, School of Medicine, University of Crete, Heraklion, Crete, Greece & Department of Anesthesiology, Onassis Cardiac Surgery Center, Athens, Greece. |
| thaslan@hotmail.com | Theodoros Aslanidis  Email: thaslan@hotmail.com  Consultant-researcher  Intensive Care Unit & Anesthesiology Department  Agios Pavlos General Hospital  Thessaloniki  Greece |
| dr.chakraraossc@gnail.com | Siddha SC Chakra Rao  dr.chakraraossc@gmail.com  Chairman Indian Resuscitation Council Federation |
| birkunalexei@gmail.com | Alexei A. Birkun, birkunalexei@gmail.com, Department of General Surgery, Anaesthesiology, Resuscitation and Emergency Medicine, Medical Institute named after S.I. Georgievsky of V.I. Vernadsky Crimean Federal University, Simferopol, Russian Federation |
| paguileraf@uc.cl | Pablo Aguilera  Associate Professor  Escuela de Medicina  Pontificia Universidad Catolica de Chile |
| tseizan@gmail.com | Seizan Tanabe, M.D., PhD.  tseizan @gmail.com  Foundation for Ambulance Service Development,  Emergency Life-Saving Technique Academy of Tokyo |
| jmfraga@emergencias.com.mx | Juan-Manuel Fraga-Sastrias  jmfraga@emergencias.com.mx  Asesores en Emergencias, Mexico |
|  | June Eva Kittivo  june.evak@gmail.com  Paramedic - various organizations |
| Kephasachiro@gmail.com | Kephas Ochieng Achiro  Email: kephasachiro@gmail.com  Trauma and Emergency Nurse |
| caroclare@yahoo.com | Caroline ndinda |
| roman.gregor@zzsmsk.cz | Roman Gregor, MD., MBA  EMS of Moravian-Silesian Region  Director |
| koen.monsieurs@uza.be | Koen Monsieurs  koen.monsieurs@uza.be  Emergency Department  Antwerp University Hospital and University of Antwerp |
| Naseef.abdullah@westerncape.gov.za | Naseef Abdullah  naseef.abdullah@westerncape.gov.za  Western Cape Government Emergency Medical Services |
| loghmaridorra@gmail.com | Dorra loghmari ,Emergency médical service 03 Sahloul university hospital,Sousse ,Tunisia |
| marcallen86@gmail.com | Marc Allen  marcallen86@gmail.com  Paramedic |
| david.stanton@netcare.co.za | David Stanton  Netcare. and  Resuscitation Council of Southern Africa  David.stanton@netcare.co.za |
| nilm314@gmail.com | Dr Nilmini Wijesuriya  nilm314@gmail.com  National coordinator Resuscitation council Sri Lanka |
| jossewanyana@gmail.com | Josephine Ssewanyana  jossewanyana@gmail.com  Queen's University Belfast |
| drfjmgaerlan@gmail.com | FAITH JOAN MESA-GAERLAN, MD |
| iwami.taku.8w@kyoto-u.ac.jp | Taku Iwami  Kyoto University |
| aarbakhsh@kau.edu.sa | Abdullah Bakhsh, MBBS  Associate Professor  Faculty of Medicine, Emergency Medicine  King Abdulaziz University  Jeddah, Saudi Arabia |
| aaalrawashdeh@just.edu.jo | Ahmad Alrawashdeh  aaalrawashdeh@just.edu.jo  Department of Allied Medical Sciences, Faculty of Applied Medical Sciences, Jordan University of ‎Science and Technology, Irbid, Jordan‎ |
| drivanchua@gmail.com | Chua Si Yong Ivan  drivanchua@gmail.com  Department of Emergency Medicine, Singapore General Hospital |
| miguel.soares.oliveira@gmail.com | Miguel Soares-Oliveira  miguel.soares.oliveira@gmail.com  Instituto Nacional Emergência Médica (INEM) |
| yaopengjy@163.com | Yu Cao, yuyuer@126.com, Department of Emergency Medicine, West China Hospital, Sichuan University, China  Peng Yao, yaopengjy@163.com, Department of Emergency Medicine, West China Hospital, Sichuan University, China  Yu Cao is the director of our department and research group |
| bogarben215@gmail.com | Bence Bogár, bogarben215@gmail.com, University of Pécs |
| loghmaridorra@gmail.com | dorra loghmari/associate professor in emergency medicine / loghmaridorra@gmail.com/ emergency medical service (EMS03) university sahloul hospital , sousse , tunisia |
| jlclint@gmail.com | Clint Jean Louis MD EMBA  Director Prehospital Emergency Services  Navarra Health Services  Ambulance Physician.  Founding Partner ABC Saves Lives |
| drpelinsesli@hotmail.com | Pelin Karaaslan, Associate Professor in İstanbul Medipol University Anesthesiology and Reanimation Department |
| s2dj003m@kokushikan.ac.jp | Shunsuke SAITO  Kokushikan University Graduate School of Emergency Medical System |
| liviuciocan7@yahoo.com | Liviu Ciocan  Neonatologist  CH Maubeuge / France |
| chihweisung@ntu.edu.tw | Chih-Wei Sung M.D., Ph.D.  Clinical Assistant Professor  Department of Emergency Medicine, College of Medicine, National Taiwan University, Taipei, Taiwan |
| b101100023@tmu.edu.tw | Chi-Hsin Chen  b101100023@tmu.edu.tw  Department of emergency medicine, National Taiwan University Hospital Hsinchu Branch, Hsinchu, Taiwan |
| Eciolino@uic.edu | Elizabeth Froelich  University of Illinois Chicago |
| sabine.lemoine@pompiersparis.fr | Sabine Lemoine, MD  sabine.lemoine@pompiersparis.fr  Paris Fire Brigade Medical Emergency Department, Paris, France. |
| rahmadhokkar@gmail.com | RAHMA DHOKKAR  REGIONAL HOSPITAL OF BEN AROUS  TUNISIA |
| mendyalfonse@gmail.com | Alfonse Mendy |
| musababamusbah10@gmail.com | Musa baba |
| gemi@peqqik.gl | George Michagin  e-mail: gemi@peqqik.gl  affiliation: ICU; Droning Ingrid Hospital, Nuuk, Greenland |
| jan.bakker@uc.cl | Jan Bakker  jan.bakker@uc.cl  Adjunct Professor  Pontificia Universidad Católica de Chile, Department of Intensive Care  Diagonal Paraguay 362  8330024 Santiago, Chile |
| adhish.gautam@gmail.com | Name: Dr. Adhish Gautam  Email: adhish.gautam@gmail..com  Affiliation: Doctor of Medicine |
| uto@krcmk.ru | Frolova Lesya, Crimean Republican Center of Disaster Medicine and Emergency Medical Services, Simferopol, Russian Federation |
| simon.schmdibauer@med.lu.se | Department of Anaesthesiology and Intensive Care, Skåne University Hospital, Malmö, Sweden  Center for Cardiac Arrest at Lund University, Lund, Sweden |
| birkunalexei@gmail.com | kjhg |
| rmromero3@up.edu.ph | Ricardo M. Romero  Philippine Society of Emergency Medical Technicians (PSEMT) |
| takoutsingberjo@gmail.com | Name: Berjo Takoutsing  Email:takoutsingberjo@gmail.com  Affiliations:  1. Research Department, Association of Future African Neurosurgeons, Yaounde, Cameroon  2. Research Division, Winners Foundation, Yaounde, Cameroon |
| papousekr@zzsjck.cz | MUDr. René Papoušek  Zdravotnická záchranná služba Jihočeského kraje p.o.  Czech Republic |
| peter.hilbert-carius@extern.drf-luftrettung.de | Peter Hilbert-Carius, PhD  DRF Luftrettung Station Halle  Lilienthalstraße 1  06188 Landsberg  Germany |
| marcus.rudolph@drf-luftrettung.de | T |
| tmasri@kau.edu.sa | Taha Masri, MD, MS, FIBODM  Tmasri@kau.edu.sa  Disaster Medicine Section, Department of Emergency Medicine, King Abdulaziz University Faculty of Medicine, Jeddah, Kingdom of Saudi Arabia, 80215 |
| allysein@gmail.com | HUSSEIN ALI  CGTRH |
| popelas@zzsjmk.cz | Stanislav Popela, MD, PhD  Physician :EMS South Moravian Region Czech Rep., Emergency dpt. -Faculty hospital Olomouc CZ  stanislav.popela@seznam.cz  Stanislav Popela https://orcid.org/000-0003-2577-9244 |
|  | ¨mm |
| jacqueline.eleonora@gmail.com | Emergency Department, Mater Dei hospital, Malta |
| popelas@zzsjmk.cz | Stanislav Popela, MD, PhD  popelas@zzsjmk.cz,  stanislav.popela@seznam.cz (http://orcid.org/0000-0003-2577-9244),  Emergency medicine physician |
| leo.bossaert@erc.edu | Leo Bossaert  leo.bossaert@erc.edu  University of Antwerp, Belgium |
| papousekr@zzsjck.cz | MUDr René Papoušek  Zdravotnická záchranná služba Jihočeského kraje  papousekr@zzsjck.cz |
| ondrej.franek@zzshmp.cz | Ondrej Franek, MD, EMS Prague, Czech republic |
| v.raffay@euc.ac.cy | 1. School of Medicine, European University Cyprus, Nicosia, Cyprus  2. Serbian Resuscitation Council, Novi Sad, Serbia |
| adhish.gautam@gmail.com | Name: Dr. Adhish Gautam  Email: adhish.gautam@gmail.com  Affiliation: Doctor of Medicine |
| Jacqueline.eleonora@gmail.com | Jacqueline Eleonora Ek, M.D, MRCEM.  Emergency Medicine, Mater Dei Hospital, Malta |
|  | I have already responded, so please cancel this input. |
| darren@darrenmedic.com | Darren Van Zyl  Darren@darrenmedic.com  Executive Director, Resuscitation Council of Southern Africa |
| Aldus.smith@nwu.ac.za | Aldus Smith  Aldus.smith@nwu.ac.za  North West university  POTCHEFSTROOM  South Africa |
| emstar@naver.com | Sungphil Chung, MD PhD  Professor, Emergency Medicine  Gangnam Severance Hospital, Yonsei University  Seoul, Korea |
| Mihegwacate@gmail.com | Mihegwa Catherine |
| jacklineakinyi87@gmail.com | Jackline Akinyi, Emergency Medicine Kenya Foundation |
| Michaelgriffinsomurwa3@gmail.com | Michael |
| omanid28@gmail.com | Doris Omani |
| janewangui98@yahoo.com | jane gitau |
| anthonynjeru98@gmail.com | Anthony |
| nakobaesther@gmail.com | Esther Nakoba |
| nginakyome@gmail.com | Angeline Wanza Nzyoka |
| cyprianestojr@gmail.com | Cyprian Esekon Esto  cyprianestojr@gmail.com  EMS membership |
| synnetteogolla@gmail.com | Synnette ogolla |
| anastasis@kidssavelives.gr | Anastasios Stefanakis  President \| Founder  KIDS SAVE LIVES - TA PAIDIA SOZOUN ZOES  Hellenic Humanitarian Organization |
| teresa.magalhaes@ensp.unl.pt | Teresa Magalhães  teresa.magalhaes@ensp.unl.pt  NOVA National School of Public Health, Public Health Research Centre, Comprehensive Health Research Center, CHRC, NOVA University of Lisbon,  Lisbon, Portugal |
| k-sasaki@hirokoku-u.ac.jp | Koichi SASAKI |
| ozbilginsule@gmail.com | Assoc. Prof. Dr. Sule Ozbilgin, MD.  Dokuz Eylul University Medical Faculty Department of Anaesthesiology and Intensive Care, Izmir, Turkey  PhD Student in Neuroscience in Dokuz Eylul University Medical Faculty  ozbilginsule@gmail.com  sozbilginmd@gmail.com  GSM: (+90) 5055252901 |
| jonathanlu@ntu.edu.tw | Lu, Tsung-Chien, MD, MS, PhD  Attending Physician & Clinical Assistant Professor  Department of Emergency Medicine  National Taiwan University Hospital  PhD, Biomedical and Health Informatics  University of Washington, Seattle |
| tomoyoshitamura@keio.jp | Tomoyoshi Tamura, MD, PhD, FAHA; tomoyoshitamura@keio.jp; Emergency and Critical Care Medicine, Keio University School of Medicine |
| C.a.veldhuis@umcg.nl | Carla Veldhuis  Senior nurse ICU |
| audthorl@landspitali.is | Nurse at Emergency Department at the Landspitali University Hospital Iceland |
| dsci0008@gmail.com | Dorothy Scicluna. Primary health care. Malta |
|  | Eui yeub Jung  ey950809@naver.com  Seoul national university hospital |
| anastasis@kidssavelives.gr | Anastasios Stefanakis  President \| Founder  KIDS SAVE LIVES - TA PAIDIA SOZOUN ZOES  Hellenic Humanitarian Organization |
| teresa.magalhaes@ensp.unl.pt | Teresa Magalhães  teresa.magalhaes@ensp.unl.pt  NOVA National School of Public Health, Public Health Research Centre, Comprehensive Health Research Center, CHRC, NOVA University of Lisbon,  Lisbon, Portugal |
| k-sasaki@hirokoku-u.ac.jp | Koichi SASAKI |
| ozbilginsule@gmail.com | Assoc. Prof. Dr. Sule Ozbilgin, MD.  Dokuz Eylul University Medical Faculty Department of Anaesthesiology and Intensive Care, Izmir, Turkey  PhD Student in Neuroscience in Dokuz Eylul University Medical Faculty  ozbilginsule@gmail.com  sozbilginmd@gmail.com  GSM: (+90) 5055252901 |
| loghmaridorra@gmail.com | Dorra loghmari ,Emergency médical service 03 Sahloul university hospital,Sousse ,Tunisia |
| jonathanlu@ntu.edu.tw | Lu, Tsung-Chien, MD, MS, PhD  Attending Physician & Clinical Assistant Professor  Department of Emergency Medicine  National Taiwan University Hospital  PhD, Biomedical and Health Informatics  University of Washington, Seattle |
| tomoyoshitamura@keio.jp | Tomoyoshi Tamura, MD, PhD, FAHA; tomoyoshitamura@keio.jp; Emergency and Critical Care Medicine, Keio University School of Medicine |
| takyu@kokushikan.ac.jp | Prof. Takyu Hiroshi |
| C.a.veldhuis@umcg.nl | Carla Veldhuis  Senior nurse ICU |
| audthorl@landspitali.is | Nurse at Emergency Department at the Landspitali University Hospital Iceland |
| dsci0008@gmail.com | Dorothy Scicluna. Primary health care. Malta |
|  | Eui yeub Jung  ey950809@naver.com  Seoul national university hospital |
|  |  |
| Mikael.gellerfors@ki.se | Mikael Gellerfors, MD, PhD Mikael.gellerfors@ki.se Department of Physiology and Pharmacology, Karolinska Institutet, Stockholm, Sweden Department of Perioperative Medicine and Intensive Care, Karolinska University Hospital, Stockholm, Sweden Swedish Air Ambulance (SLA), Mora, Sweden Rapid Response Car, Capio, Stockholm, Sweden |
| fergus.gardiner@rfds.org.au | Dr Fergus Gardiner Royal Flying Doctor Service of Australia Level 2, 10 12 Brisbane Avenue Barton ACT 2600 |
| stian.mohrsen@hhft.nhs.uk | Stian Mohrsen stian.mohrsen@hhft.nhs.uk Hampshire Hospitals NHS Foundation Trust |
| falmed@libero.it | Stefano Falcetta falmed@libero.it A.O.U.della Marche Ancona |
| marius.rehn@norskluftambulanse.no | Marius Rehn marius.rehn@norskluftambulanse.no Oslo University Hospital, Oslo, Norway Norwegian Air Ambulance, Oslo, Norway University of Oslo, Oslo, Norway |
| jokra@medisin.uio.no | Jo Kramer-Johansen jokra@medisin.uio.no Norwegian Cardiac Arrest Registry Division of Prehospital Services Oslo University Hospital and University of Oslo |
|  | Per P. Bredmose bredmose@hotmail.com Oslo University Hospital Air Ambulance department |
| m.kowalski@lpr.com.pl | Marcin Kowalski, MD PhD, m.kowalski@lpr.com.pl Polish Medical Air Rescue |
| oddvar.uleberg@stolav.no | Oddvar Uleberg oddvar.uleberg@stolav.no St. Olavs University Hospital Department of Emergency Medicine and Pre-hospital services N-7006 Trondheim Norway |
| m.menarini@118er.it | Maurizio Menarini EMS service Director AUSL della Romagna Ravenna - Italy |
| peter.martin.hansen@rsyd.dk | Peter Martin Hansen, M.D. Danish Air Ambulance, Aarhus, Denmark Mobile Emergency Care Unit, Dept. of anesthesiology and intensive care medicine, Odense University Hospital Svendborg |
| alasdair.corfield@ggc.scot.nhs.uk | Prof Alasdair R Corfield ScotSTAR / Univeristy of Glasgow alasdair.corfield@ggc.scot.nhs.uk |
| alasdair.corfield@ggc.scot.nhs.uk | Prof Alasdair R Corfield ScotSTAR / University of Glasgow alasdair.corfield@ggc.scot.nhs.uk |
| pierre-nicolas.carron@chuv.ch | Prof Pierre-Nicolas Carron Emergency Department Lausanne University Hospital and Lausanne University Bugnon 44 CH 1011 Lausanne Switzerland pierre-nicolas.carron@chuv.ch |
| thomas.wilson@finnmarkssykehuset.no | Thomas Wilson, MD Medical director, ambulance helicopter Kirkenes, Finnmark health trust, Skytterhusveien 4, 9901 Kirkenes, Norway Consultant anaesthesiologist Operations- and intensive care department, University hospital of northern Norway, P.O. box 100, 9038 Tromsø, Norway |
| petertemesv@gmail.com | dr. Peter Temesvari Pre-Hospital Emergency Medicine Consultant Hungarian Air Ambulance |
| johannes.bjorkman@hus.fi | Johannes Björkman, MD, PhD johannes.bjorkman@hus.fi Emergency Medicine Services, Helsinki University Hospital, and Department of Emergency Medicine, University of Helsinki, Helsinki, Finland |
| troehans@rm.dk | Troels Martin Hansen Medical Director Danish Air Ambulance troehans@rm.dk |
| timo.iirola@varha.fi | Timo Iirola, MD, PhD timo.iirola@varha.fi Emergency Medical Services, Turku University Hospital and University of Turku, Turku, Finland |
| florian.reifferscheid@drf-luftrettung.de | Dr. med. Florian Reifferscheid Florian.Reifferscheid@drf-luftrettung.de DRF Stiftung Luftrettung gAG |
| moboet@rm.dk | Morten Thingemann Bøtker, Prehospital Emergency Medical Services, Central Denmark Region |
| derkow@poczta.onet.pl | Tomasz Derkowski MD,PhD, Szpital Czerniakowski Anaesthetic Department |
| thomas.werner.lindner@sus.no | Thomas W. Lindner RAKOS Stavnger Health Trust Stavanger Norway |
| robert.gebei@airambulance.hu | Róbert Gebei MD. Hungarian Air Ambulance Ltd. |
| laurent.suppan@hcuge.ch | Laurent Suppan, Division of Emergency Medicine , Department of Anesthesiology, Clinical Pharmacology, Intensive Care and Emergency Medicine , University of Geneva Hospitals and Faculty of Medicine , Geneva , CH |
| twt2@u.washington.edu | Timothy Thornton twt2@u.washington.edu Providence St. Joseph Medical Center |
| lars.jacobsen@sshf.no | Lars Jacobsen lars.jacobsen@sshf.no Sorlandet Sykehus HF/ University of Oslo |
| Martin.Nichols@health.nsw.gov.au | Martin Nichols Martin.Nichols@health.nsw.gov.au NSW Ambulance |
| helena.jantti@pshyvinvointialue.fi | PhD Helena Jäntti Prehospital Emergency Care Kuopio University Hospital Finland |
| dbarten@viecuri.nl | Dennis G. Barten (dbarten@viecuri.nl), Department of Emergency Medicine, VieCuri Medical Center, Venlo, the Netherlands |
| andy.swain@wfa.org.nz | Andrew H Swain andy.swain@wfa.org.nz Wellington Free Ambulance, New Zealand |
| leifrogn@rm.dk | A Prof Leif Rognås PhD Base Clinical Lead, Danish Air Ambulance leifrogn@rm.dk |
| Thomas.bech.lunen@regionh.dk | Thomas Bech Lunen Thomas.bech.lunen@regionh.dk Ledende overlæge Ambulance Akutberedskabet |
| martinek@med.umich.edu | Christine Brent, MD martinek@med.umich.edu University of Michigan |
| Geert-Jan.vanGeffen@RAdboudumc.nl | Geert-Jan van Geffen Geert-Jan.vanGeffen@RAdboudumc.nl Radboud University Medical Centre Nijmegen The Netherlands |
| sophronesis@gmail.com | First name: Andrew Fu Wah Last name: Ho email: andrew.ho@duke-nus.edu.sg Affiliations: 1. Department of Emergency Medicine, Singapore General Hospital, Singapore 2. Pre-hospital & Emergency Research Centre, Duke-National University of Singapore Medical School, Singapore 3. Centre for Population Health Research and Implementation, SingHealth Regional Health System, Singapore 4. Saw Swee Hock School of Public Health, National University of Singapore, Singapore |
| t.m.olasveengen@medisin.uio.no | Theresa Mariero Olasveengen t.m.olasveengen@medisin.uio.no Oslo University Hospital and University of Oslo |
| jacob.steinmetz@regionh.dk | Jacob Steinmetz jacob.steinmetz@regionh.dk Danish Air Ambulance |
| edward.tan@radboudumc.nl | Edward C.T.H. Tan, MD, PhD Associate Professor Traumasurgeon HEMS Physician, Lifeliner 3 Lieutenant-Colonel (R) Royal Netherlands Army edward.tan@radboudumc.nl T (+31 24) 36 138 71 / +31 24 36 104 92 Radboud university medical center PO Box 9101 (618), 6500 HB Nijmegen Geert Grooteplein 10, 6525 GA Nijmegen (route 618) The Netherlands www.radboudumc.nl |
|  | Andrew Herbert Swain andy.swain@wfa.org.nz Wellington Free Ambulance and AUT University, Auckland |
| jvopelius@ornge.ca | Johannes von Vopelius-Feldt jvopelius@ornge.ca Emergency Department, St. Michael's Hospital Toronto, Ontario Ornge, Ontario |
| david.reid@stjohn.wa.com.au | Assoc. Professor David Reid david.reid@stjohnwa.com.au Edith Cowan University St John Ambulance WA |
| amund.formo@sykehuset-innlandet.no | Amund Formo, amund.formo@sykehuset-innlandet.no HEMS physician, Sykehuset Innlandet, prehospital divisjon |
| trondelden@gmail.com | Trond Elden Anaesthesiologist Nordland County General Hospital |
| berg.ingvar@gmail.com | Ingvar Thore Benno Berg (I.T.B.Berg) Emergency Physician Email: i.berg@haaglandenmc.nl Affiliation: Department of Emergency Medicine Haaglanden Medical Centre The Hague, The Netherlands |
| Geert-Jan.vanGeffen@Radboudumc.nl | Geert-Jan.vanGeffen@Radboudumc.nl Radboud University Medical Centre Nijmegen The Netherlands |
| na.anderson@auckland.ac.nz | Dr Natalie Anderson na.anderson@auckland.ac.nz University of Auckland / Auckland Emergency Department |
| jouni.nurmi@hus.fi | Jouni Nurmi jouni.nurmi@hus.fi Helsinki University Hospital and University of Helsinki |
| jouni.kurola@pshyvinvointialue.fi | Jouni Kurola, MD, PhD jouni.kurola@pshyvinvointialue.fi University of Eastern Finland, Kuopio, Finland Centre for prehospital emergency care, Kuopio University Hospital, Kuopio, Finland |
| chemanp@gmail.com | Jose Mª Navalpotro Pascual. Physician of SUMMA 112 Madrid. chemanp@gmail.com |
| j.seesink@erasmusmc.nl | Jeroen Seesink, MD, MSc j.seesink@erasmusmc.nl Department of Anaesthesiology, Erasmus MC University Medical Centre, Rotterdam, The Netherlands |
| mari.stokstad.olsen@sshf.no | Mari Stokstad Olsen Arendal Hospital. mari.stokstad.olsen@sshf.no |
| Holwem@rm.dk | Holger Wemmelund holwem@rm.dk Operation og Intensiv Regionshospitalet Gødstrup |
| robb.devries@cantonmi-gov | Robb Anthony DeVries Canton Township Fire Department robb.devries@cantonmi.gov |
| gvdploeg@vrnhn.nl | - Gert-Jan van der Ploeg - gvdploeg@vrnhn.nl - MD, Medical Manager Ambulance Service Noord-Holland Noord - Alkmaar, The Netherlands |
| samuel.perillo@act.gov.au | Sam Perillo samuel.perillo@act.gov.au Australian Capital Territory Ambulance Service |
| mwayne@whatcomcounty.us | Marvin Wayne, MD, FACEP, FAAEM, FAHA EMS Medical Director Whatcom Couty WA Associate Clinical Professor Dept of Emergency Medicine, University of Washington |
| Gerard.Job@miamidade.gov | Gerard Job MD, FACEP Medical Director Miami Dade Fire Rescue Core EM Faculty Jackson Memorial Hospital Emergency Care Center Affiliated Faculty of Surgery University of Miami Miller School of Medicine Disaster Liaison/Clinical Instructor Disaster and Emergency Preparedness |
| conrad.bjorshol@sus.no | Conrad Arnfinn Bjørshol, senior researcher The Regional Centre for Emergency Medical Research and Development (RAKOS), Anaesthesiologist Stavanger University Hospital |
| jeffrey.ferguson@vcuhealth.org | Jeffrey D Ferguson, MD Jeffrey.ferguson@vcuhealth.org Virginia Commonwealth University Henrico County Division of Fire |
| Kenneth.Miller@ems.sccgov.org | Ken Miller MD PhD Medical Director Santa Clara County (CA) Emergency Medical Services Agency Kenneth.Miller@ems.sccgov.org |
|  | Christie Fritz, MD cfritz@bidmc.harvard.edu Beth Israel Deaconess Medical Center Boston, MA |
| jeremy.dewall@uchealth.org | Jeremy DeWall, MD, FAEMS, NRP Office of the Medical Director Pikes Peak, Southern, & Southeastern Colorado Regions Jeremy.dewall@uchealth.org |
| stilleyjd@health.missouri.edu | Joshua Stilley, MD stilleyjd@health.missouri.edu University of Missouri |
| Bernhardsonems@gmail.com | Noah Bernhardson, MD EMSOA/Lincoln Fire Medical Director Bernhardsonems@gmail.com |
|  | Angela P Cornelius MD MA angiepmd03@gmail.com Office of the Medical Director MAEMSA Medstar Mobile Mobile Healthcare Fort Worth EM Residency John Peter Smith Hospital |
| eric.wu.md@gmail.com | Eric Wu eric.wu.md@gmail.com Colorado Springs FD |
| woltmanMD@gmail.com | Nathan Woltman MD Abu Dhabi Department of Health |
|  | Randy Katz, DO, FACEP rkatz@mhs.net City of Hollywood, Florida, EMS and Beach Safety |
| sanjuanmpd@gmail.com | Joshua G. Corsa, MD San Juan County EMS sanjuanmpd@gmail.com |
| jjarvis@medstar911.org | Jeffrey L. Jarvis, MD, MS, EMT-P jjarvis@medstar911.org Chief Medical Officer & System Medical Director Metropolitan Area EMS Authority, dab MedStar Fort Worth, Texas USA |
| mwayne@whatcomcounty.us | Marvin A Wayne, MD, FACEP, FAAEM, FAHA |
|  | DOUGLAS C. GRUZD, MD,FACEP gruzddc@aol.com Medical Director Crook County Rire & Rescue, Medical Director for 4 more volunteer ambulance agencies in wilderness Central Oregon |
| Jessicawentling@gmail.com | Jessica Wentling, DO FAAEM FACEP jessicawentling@gmail.com Texas Regional Medical Director Air Evac Lifeteam |
| dgp3a@uvahealth.org | Debra Perina, MD Professor Emeritus, University of Virginia dgp3a@uvahealth.org |
| newflane00@gmail.com | Charles J. Lane, MD Newflane00@gmail.com Assistant Professor. Virginia Tech-Carilion School of Medicine, EMS Fellowship |
| jeffrey-goodloe@ouhsc.edu | Jeffrey M. Goodloe, MD, NRP, FACEP, FAEMS jeffrey-goodloe@ouhsc.edu Professor and EMS Section Chief, Department of Emergency Medicine, University of Oklahoma School of Community Medicine, Tulsa, OK; Chief Medical Officer, EMS System for Metropolitan Oklahoma City and Tulsa, Oklahoma |
| alex.zozula@gmail.com | Alexander Zozula, MD, FAEMS alex.zozula@gmail.com EMS Medical Director Alton Memorial EMS System Alton, IL, USA |
| joeholley@gmail.com | Joseph E Holley, MD FACEP FAEMS University of Tennessee Health science Center at Memphis Emergency Medicine Memphis Fire Department joeholley@gmail.com |
| sayrem@uw.edu | Michael R. Sayre sayrem@uw.edu University of Washington, USA |
|  | Dr. Russell D. MacDonald, MD MPH FCFP FRCPC DRCPSC; Medical Director, Toronto Paramedic Services and Toronto Central Ambulance Communication Centre; Professor, Faculty of Medicine, University of Toronto russell.macdonald@toronto.ca |
| sjvdo@hotmail.com | Stephen J Vetrano DO FACOEP FACEP EMT sjvdo@hotmail.com Medical Director Township of Neptune NJ |
| Matthew.loconte@gmail.com | Matthew LoConte, MD Matthew.loconte@gmail.com UMass Memorial Healthcare |
| ddarsey@darseyservicesgroup.com | Damon Darsey, MD, FAEMS Medical Director Mississippi Department or Public Safety Ddarsey@darseyservicesgroup.com |
| cathal.odonnell@hse.ie | Prof Cathal O'Donnell Clinical Director National Ambulance Service Ireland cathal.odonnell@hse.ie |
| vaba@peqqik.gl | Vagn Bach Chief medical Offier, MPA Department af Operations and Intensiv Care Queen Ingrids Hospital Nuuk, Greenland vaba@peqqik.gl |
| emsmd@emsmedicaldirector.net | Glenn Burket III, DO EMSMD@EMSMedicalDirector.net Mesa County EMS |
| lybeck@mep.health | Aurora Lybeck lybeck@mep.health Madison Emergency Physicians |
| tchassee@gmail.com | Todd Chassee MD FACEP, Associate Professor Emergency Medicine Michigan State University College of Human Medicine, Medical Director KCEMS |
| dettpvelasco@yahoo.com | Bernadett Pua Velasco, MD, FPCEM Department of Health dettpvelasco@yahoo.com |
| jahlelawatiz13@gmail.com | Jahlelawati Zul jahlelawatiz13@gmail.com Emergency Physician Fellow in Prehospital and Disaster Medicine Head of Prehospital Unit, Emergency and Trauma Department, Hospital Raja Permaisuri Bainun, Ipoh Malaysia |
| rmromero3@up.edu.ph | Ricardo M. Romero, DR&DM Philippine Society of Emergency Medical Technicians (PSEMT) |
| rebecka.rubenson.wahlin@ki.se | Rebecka Rubenson Wahlin \| MD, Ph.D Research group leader \| Division of Anesthesia and Intensive care Department of Clinical Science and Education \| Karolinska Institutet Sjukhusbacken 10 \| 118 83 Stockholm, Sweden + 46 (0)708- 166994 rebecka.rubenson.wahlin@ki.se \| ki.se |
| hajriz.a@hotmail.com | Hajriz Alihodžić, hajriz.a@hotmail.com e Emergency Medical Service, Public Institution Health Centre "Dr. Mustafa Šehović" and Faculty of Medicine, University of Tuzla, Tuzla, Bosnia and Herzegovina |
| Martin.rief@medunigraz.at | Martin Rief, MD PhD, Division of Anaesthesiology and Intensive Care Medicine, Medical University of Graz, Graz, Austria |
| maurolopesmota@gmail.com | Mauro Mota Health School of the Polytechnic Institute of Viseu, Portugal Health Sciences Research Unit: Nursing (UICISA: E), Nursing School of Coimbra (ESEnfC), Portugal. |
| michael.eichinger@medunigraz.at | Michael Eichinger MD MSc DESAIC michael.eichinger@medunigraz.at Department of Anaesthesiology and Intensive Care Medicine, Medical University of Graz |
| stephan.katzenschlager@med.uni-heidelberg.de | Stephan Katzenschlager stephan.katzenschlager@med.uni-heidelberg.de Medical Faculty Heidelberg, Department of Anesthesiology, Heidelberg University |
| helabenturkia@gmail.com | Hela ben Turkia helabenturkia@gmail.com Emergency department of regional hospital of Ben Arous Tunisia Faculty of medecine of Tunis Tunis El Manar university |
| wiem.barbaria@gmail.com | wiem Barbaria wiem.barbaria@gmail.com service de pédiatrie et de néonatologie Hopital Habib Bougatfa de Bizerte- Tunisie |
